# Identification of FAM111A as an SV40 Host Range Restriction and Adenovirus Helper Factor

**DOI:** 10.1371/journal.ppat.1002949

**Published:** 2012-10-18

**Authors:** Debrah A. Fine, Orit Rozenblatt-Rosen, Megha Padi, Anna Korkhin, Robert L. James, Guillaume Adelmant, Rosa Yoon, Luxuan Guo, Christian Berrios, Ying Zhang, Michael A. Calderwood, Soundarapandian Velmurgan, Jingwei Cheng, Jarrod A. Marto, David E. Hill, Michael E. Cusick, Marc Vidal, Laurence Florens, Michael P. Washburn, Larisa Litovchick, James A. DeCaprio

**Affiliations:** 1 Department of Medical Oncology, Dana-Farber Cancer Institute, Boston, Massachusetts, United States of America; 2 Program in Virology, Harvard University Graduate School of Arts and Sciences, Division of Medical Sciences, Boston, Massachusetts, United States of America; 3 Genomic Analysis of Network Perturbations Center of Excellence in Genomic Science, Center for Cancer Systems Biology, Dana-Farber Cancer Institute, Boston, Massachusetts, United States of America; 4 Center for Cancer Computational Biology, Department of Biostatistics and Computational Biology and Department of Cancer Biology, Dana-Farber Cancer Institute, Boston, Massachusetts, United States of America; 5 Department of Biostatistics, Harvard School of Public Health, Boston, Massachusetts, United States of America; 6 Center for Cancer Systems Biology and Department of Cancer Biology, Dana-Farber Cancer Institute, Boston, Massachusetts, United States of America; 7 Blais Proteomics Center and Department of Cancer Biology, Dana-Farber Cancer Institute, Boston, Massachusetts, United States of America; 8 Department of Biological Chemistry and Molecular Pharmacology, Harvard Medical School, Boston, Massachusetts, United States of America; 9 Department of Medicine, Brigham and Women's Hospital, Harvard Medical School, Boston, Massachusetts, United States of America; 10 Stowers Institute for Medical Research, Kansas City, Missouri, United States of America; 11 Department of Genetics, Harvard Medical School, Boston, Massachusetts, United States of America; 12 Department of Cancer Biology, Dana-Farber Cancer Institute, Boston, Massachusetts, United States of America; 13 Department of Pathology and Laboratory Medicine, The University of Kansas Medical Center, Kansas City, Kansas, United States of America; University of Michigan, United States of America

## Abstract

The small genome of polyomaviruses encodes a limited number of proteins that are highly dependent on interactions with host cell proteins for efficient viral replication. The SV40 large T antigen (LT) contains several discrete functional domains including the LXCXE or RB-binding motif, the DNA binding and helicase domains that contribute to the viral life cycle. In addition, the LT C-terminal region contains the host range and adenovirus helper functions required for lytic infection in certain restrictive cell types. To understand how LT affects the host cell to facilitate viral replication, we expressed full-length or functional domains of LT in cells, identified interacting host proteins and carried out expression profiling. LT perturbed the expression of p53 target genes and subsets of cell-cycle dependent genes regulated by the DREAM and the B-Myb-MuvB complexes. Affinity purification of LT followed by mass spectrometry revealed a specific interaction between the LT C-terminal region and FAM111A, a previously uncharacterized protein. Depletion of FAM111A recapitulated the effects of heterologous expression of the LT C-terminal region, including increased viral gene expression and lytic infection of SV40 host range mutants and adenovirus replication in restrictive cells. FAM111A functions as a host range restriction factor that is specifically targeted by SV40 LT.

## Author Summary

Viruses have evolved numerous mechanisms to counteract host cell defenses to facilitate productive infection. Simian Virus 40 (SV40) replication depends on specific interactions between large T antigen (LT) and a wide variety of host cell proteins. Although the LT C-terminal region has no evident enzymatic activity, mutations or deletions of this region significantly reduce the ability of the virus to replicate in restrictive cell types. Here, we identified host proteins that bind to LT and determined that the LT C-terminal region binds specifically to FAM111A. This physical interaction was required for efficient viral replication and sustained viral gene expression in restrictive cell types. In addition, RNAi-mediated knockdown of FAM111A levels in restrictive cells restored lytic infection of SV40 host range mutants and human adenovirus. These results indicate that FAM111A plays an important role in viral host range restriction. Our study provides insights into the viral-host perturbations caused by SV40 LT and the interaction of viruses with host restriction factors.

## Introduction

SV40 large T antigen (LT) is a multifunctional viral protein that plays a central role in orchestrating productive viral infection as well as cellular transformation. Discrete regions of LT are required for binding to specific host proteins and provide specific functions. The LXCXE motif (residues 103–107) binds to the retinoblastoma family of proteins RB (RB1), p107 (RBL1) and p130 (RBL2) to promote cell cycle entry. The N-terminal J domain (residues 1–82) binds specifically to heat shock protein chaperone HSC70 (HSPA4) and contributes to efficient viral replication as well as inactivation of p107 and p130 growth suppressing activities [1], [2]. The LT DNA binding domain (DBD; residues 131–251) binds specifically to the SV40 DNA origin of replication. The central domain (residues 260 to 627) contributes to LT hexamer formation, contains intrinsic ATPase and helicase activity, and binds p53 [3]–[5]. The C-terminal region (residues 627–708) contains no known structural domains but does undergo specific post-translational modifications, including acetylation of lysine residue 697 (K697) and phosphorylation of threonine 701 (T701), the latter required for LT binding to FBXW7 [6], [7]. In addition, an intact LT C-terminal region is required for the host range and adenovirus helper functions of SV40 [8], [9].

Viral host range is defined as the set of cells, tissues and species that a virus can productively infect. There are a wide variety of cellular host range restriction factors as well as counter strategies employed by viruses to overcome them. Sometimes virally encoded proteins bind directly to specific host proteins to overcome host range restriction. SV40 host range mutant viruses, all of which contain deletions or truncations in the C-terminal region of LT, express lower levels of mRNA and protein for early (LT) and late (VP1) genes compared to wild type virus and fail to support lytic infection in restrictive cell types [10], [11]. Heterologous expression of the C-terminal region of LT in trans leads to increased early and late gene expression of host range mutant virus and rescues the ability of these mutant viruses to induce lytic infection in restrictive cells [10], [12]. In addition, the C-terminal region of LT is required for the adenovirus helper effect; human adenoviruses are unable to replicate in certain monkey cell lines unless SV40 is also present [13]. The LT C-terminal region contributes a discrete activity that supports replication of SV40 and adenovirus in restrictive cell lines although it is uncertain whether these activities reflect the same function.

Here, we examined host interactome and transcriptome perturbations induced by full-length and discrete functional domains of LT. The resulting data provides a global view of LT-host cell interactions and highlights cellular pathways perturbed by the presence of LT. Notably, we identified FAM111A, a previously uncharacterized cellular protein that binds specifically to the C-terminal region of LT. We provide evidence that this interaction contributes to SV40 host range and adenovirus helper functions.

## Results

### SV40 LT C-terminal region increases viral gene expression in monkey and human cells

**Figure 1:**
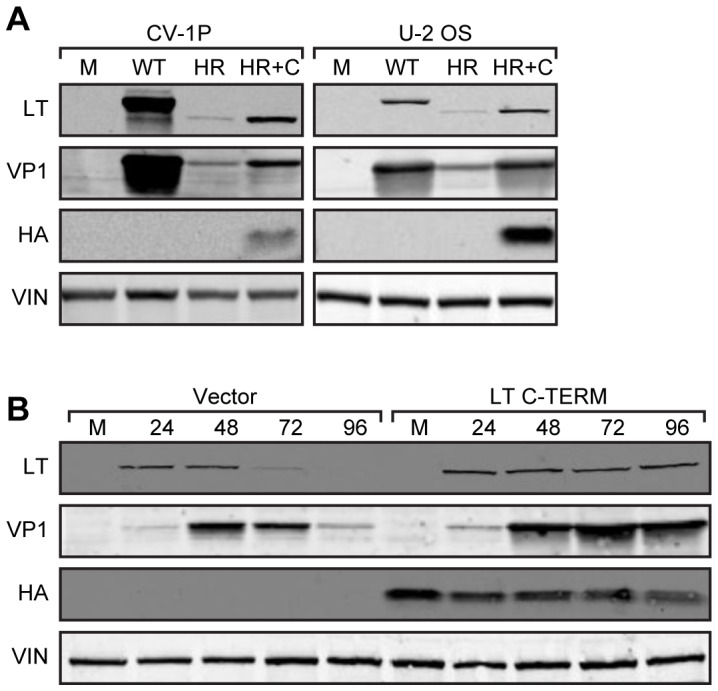
Expression of SV40 LT C-terminus increases early and late viral gene levels. (A) CV-1P or U-2 OS cells were transfected with vector control (M), viral DNA encoding wild type SV40 (WT), host range mutant HR684 (HR), or HR plus HA-tagged LT C-TERM (HR+C). Ninety-six hours post-transfection, cell lysates were western blotted for LT, VP1, LT C-TERM (HA) and vinculin (VIN). (B) U-2 OS cells stably expressing vector only or LT C-TERM were transfected with HR684 viral DNA. Lysates were prepared at the indicated time points (hours) and blotted as in (A).

The C-terminal region of LT is required for efficient viral gene expression and replication in the African green monkey kidney (AGMK) CV-1P cell line [8], [9]. The SV40 host range mutant virus HR684 lacks the C-terminal 24 residues of LT and has significantly reduced expression of early (LT) and late viral (VP1) genes compared to wild type virus in CV-1P cells (Figure 1A; [10]). Heterologous expression of the LT C-terminal 82 residues (C-TERM; residues 627 to 708) markedly increased levels of HR684 LT and VP1 in these cells [10]. Since LT C-TERM could support increased viral gene expression in trans, we suspected that this LT fragment could bind to a specific host cell factor and thereby increase viral gene expression.

Proteomic analysis was not possible in AGMK cells because whole genome and proteome sequences were not available. Instead, we tested several human cell lines for the ability of the LT C-terminal region to increase levels of host range mutant viral genes. Increased levels of HR684 LT and VP1 were observed in U-2 OS but not in HeLa or T98G cells when co-expressed with LT C-TERM (Figure 1A and Figure S1 in Text S1). Given the ability of C-TERM to increase HR684 gene expression in U-2 OS and CV-1P cells, we selected the U-2 OS cell line to further analyze of the host range phenotype.

To examine the effect of the LT C-terminal region on LT and VP1 levels, HR684 viral DNA was transfected into U-2 OS cells that stably expressed the C-TERM construct or empty vector (Figure 1B). LT could be detected at 24 hours and VP1 at 48 hours after transfection in both cell lines. While levels of LT and VP1 decreased at 72 and 96 hours respectively in the vector control cells, both LT and VP1 show persistent expression at 72 and 96 hours after transfection in the LT C-TERM containing cell line (Figure 1B). This result indicates that the LT C-terminus functions at least in part to sustain viral gene expression in U-2 OS cells.

### LT-host transcriptome perturbations

**Figure 2:**
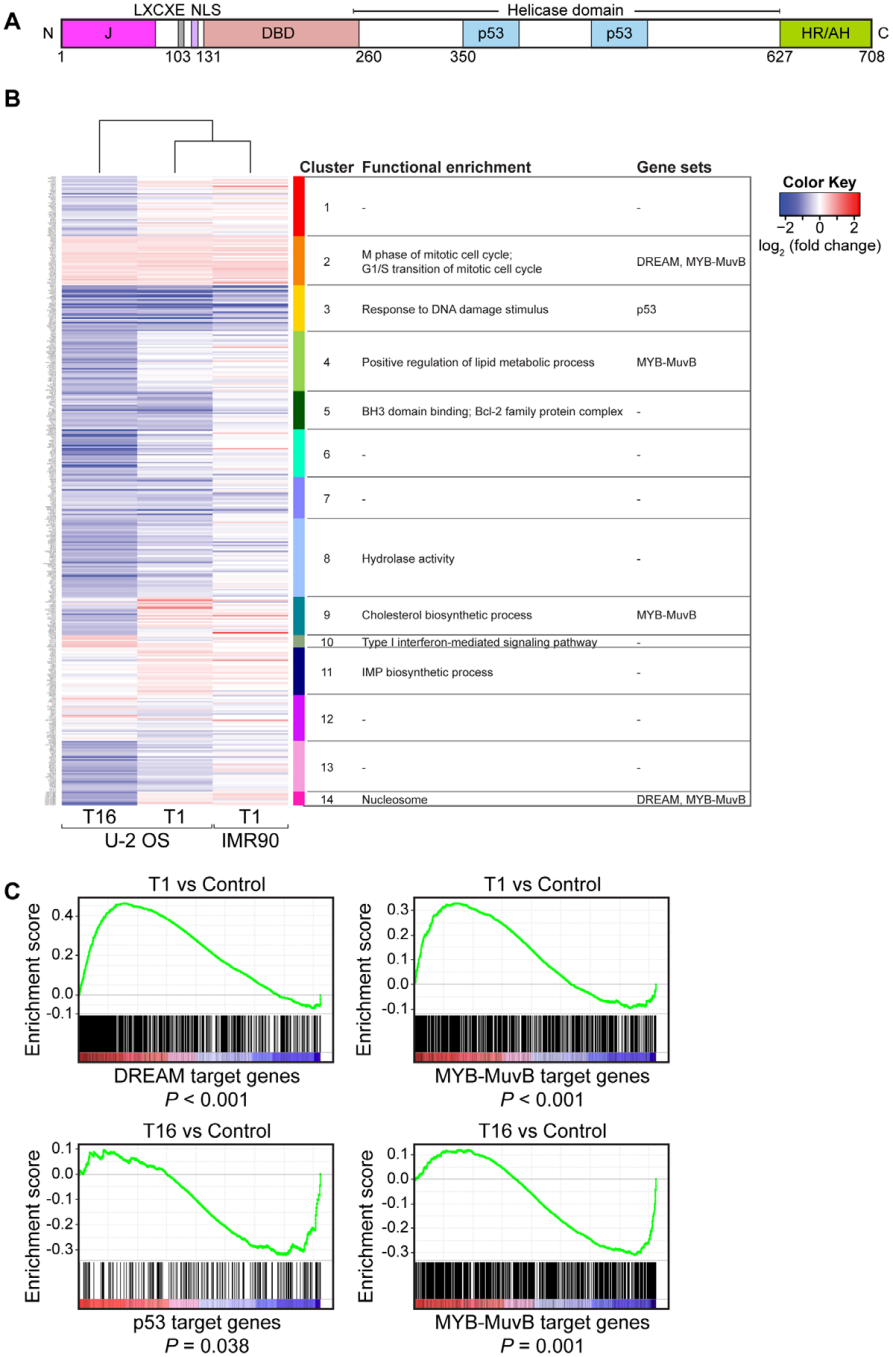
Transcriptome perturbations induced by LT. (A) Schematic representation of the SV40 LT protein. Functional domains including the J domain, the LXCXE or RB binding motif, the nuclear localization signal (NLS), the DNA binding domain (DBD), the bipartite p53 binding domain contained within the helicase domain, and the C-terminal host range (HR)/adeno-helper (AH) domain are depicted. Residue numbers indicate limits for LT functional domains (B) Gene clusters that showed functional enrichment upon expression of full-length LT (T1) or LT fragments. The heatmap shows the expression of these genes in U-2 OS cells expressing T1 or T16 and IMR-90 cells expressing T1 relative to vector or GFP controls, respectively. Replicates were collapsed and genes hierarchically clustered (rows, genes; columns, experiments; red, induced from baseline; blue, repressed from baseline; white, unchanged from baseline). Enriched GO terms are listed adjacent to the numbered expression clusters and next to them are listed enriched gene sets in the cluster. In cluster C14 all transcripts are histones (C) GSEA plots determining whether the expression of the defined gene sets (DREAM, B-Myb-MuvB, or p53) show statistically significant, concordant differences between two biological states (T1 or T16 and vector control).

Discrete functional domains within the SV40 LT protein bind to diverse host cell proteins (Figure 2A). We generated LT expression constructs encoding epitope-tagged fusions of full-length LT as well as fragments corresponding to computationally- and functionally-defined domains. Full-length LT (T1), the LT N-terminal region encoded by residues 1 to 135 (T6 fragment) or residues 1 to 350 (T8 fragment), and the LT C-terminal region between residues 260 and 708 (T16 fragment) were stably expressed in U-2 OS cells (Figure S2 in Text S1).

We determined the effects of full-length LT and various fragments on global gene expression. Cells expressing T1 and T16 constructs showed significant differential expression changes of multiple target genes compared to control. In contrast, cells expressing the N-terminal T6 and T8 constructs showed minimal changes in gene expression compared to control. To identify patterns of host transcriptional perturbation common across all comparisons between the set of LT constructs and controls, we applied model-based clustering to construct clusters from the 430 most frequently perturbed host genes (Table S1 in Text S1). Of the 14 identified clusters, 9 exhibited significantly enriched GO terms (Figure 2B). Heterologous expression of T1 or T16 led to increased expression of genes involved in the cell cycle (cluster C2), regulated by the DREAM and MYB-MuvB complexes [14], [15], and decreased expression of genes in cluster 3 (Figure 2B and Table S2 in Text S1), enriched for p53 target genes. We compared the transcriptional perturbations induced by T1 in U-2 OS cells with an earlier study performed of T1 in normal human diploid IMR90 fibroblasts [16]. We found that the p53- and the DREAM-regulated pathways were similarly perturbed in both cell types (Figure 2B) [16].

To assess the biological significance of the expression profiles we applied gene set enrichment analysis (GSEA) [17]. A significant enrichment for increased expression of DREAM and MYB-MuvB gene sets was observed in T1-expressing cells (Figure 2C). In contrast, there was significant enrichment for decreased expression of p53 genes and MYB-MuvB genes in the T16-expressing cells. These results suggest that full-length LT (T1) and the C-terminal T16 portion exert distinct transcriptional perturbations in U-2 OS cells.

### Mapping the LT interactome

**Figure 3:**
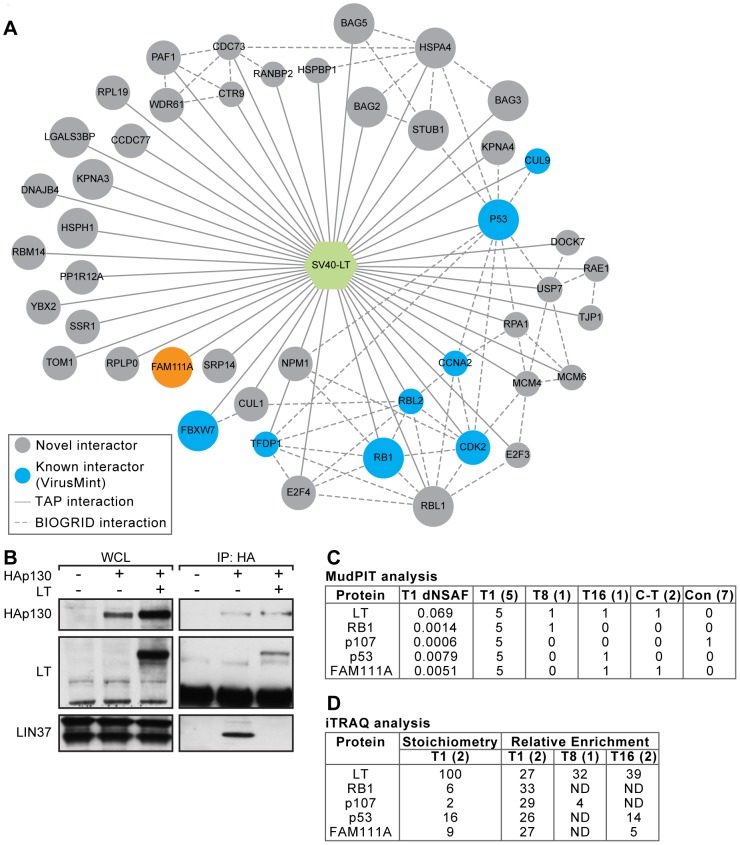
Associations between T1 and host proteins. (A) Network of associations of full-length SV40 LT (hexagon) with host proteins (circles) detected in at least three of five replica affinity purification (AP)-MudPIT experiments. Host proteins reported to associate with LT in VirusMint are colored (Blue). Circle size is proportional to the number of times the association was observed. Solid lines (links) represent viral-host protein associations and dashed lines represent host-host associations reported in the BioGRID database. (B) Extracts from T98G cells transfected with HA-tagged p130 and LT were immunoprecipitated with anti-HA antibodies and the indicated proteins were detected by western blot. (C) Summary of AP-MudPIT analysis from full-length LT or LT fragments for the indicated host proteins. Relative abundance values (dNSAF as defined in the supplemental data) were averaged across 5 biological replicate analyses of T1 (full-length LT) affinity purification experiments. The number of times each host protein was identified in the biological replicates is shown. CT indicates C-TERM. (D) Summary of iTRAQ analysis. Estimates of protein stoichiometry, relative to LT were based on reconstructed ion chromatogram (RIC) intensities of the most abundant peptides assigned to each protein. Number of biological replicates for each affinity purification experiment is indicated in parentheses in header. ND indicates not detected.

Given the effects of LT on cellular and viral gene expression, we sought to identify host proteins that bind to LT. We used multidimensional protein identification technology (MudPIT) to analyze preparative scale immunoprecipitations by mass spectrometry [18]. We pooled the results of five independent experiments from cells expressing full-length T1. In total, we identified 89 proteins that co-purified with T1 and were detected in at least three out of five independent affinity purification experiments (Figure 3A and Table S3 in Text S1). We detected several previously reported interactors of LT including FBXW7 and p53 [19]. In addition, we identified co-complexes with RB family proteins and E2F/DP1 transcription factors, but not any of the five proteins contained within the MuvB core complex (LIN9, LIN37, LIN52, LIN54 and RBBP4) that bind to p130 (RBL2) in the DREAM complex [14]. Immunoprecipitation for p130 co-precipitated the MuvB subunit LIN37 in the absence of LT but not when LT was present (Figure 3B), further supporting the conclusion that LT disrupts the DREAM complex.

Consistent with previous reports that the N-terminal J domain binds HSC70 [1], [2], [20], [21], we observed specific association with HSPA4. We also identified HSPH1 (Hsp105), STUB1 (CHIP) and members of the BAG protein family, including BAG2, BAG3 and BAG5. BAG proteins bind to HSC70 to inhibit ubiquitination of misfolded proteins by STUB1 [22], [23]. We also detected LT association with members of the PAF1 transcription elongation complex PAF1C, including CDC73, PAF1, CTR9 and WDR61 [24]–[26].

We detected a previously unreported association of LT with the uncharacterized protein FAM111A (family with sequence similarity 111, member A; LOC63901; Gene ID: 63901). FAM111A was reproducibly detected in all five full-length LT (T1) replicates but not in the corresponding negative controls (Figure 3C and Table S3 in Text S1). The T16 and C-TERM LT fragments also showed association with FAM111A (Figure 3C), indicating that the C-terminal region of LT was sufficient for association with FAM111A.

We also identified LT-associated proteins using iTRAQ stable isotope labeling (see Supplementary experimental procedures and Table S4 in Text S1). Full-length LT (T1) showed associations with RB, p107, p53, and FAM111A (Figure 3D). In addition, the T1 and T8 constructs bound p107 while T1 and T16 fragments bound to p53 and FAM111A. The extensive sequence coverage of FAM111A (62.4%; Figure S3 in Text S1) and high normalized spectral abundance factor (dNSAF) values observed in the MudPIT analyses of the T1 affinity purification (Figure 3C), as well as the strong enrichment relative to the negative control by iTRAQ analysis, suggest that LT binds FAM111A efficiently.

### Mapping the LT-FAM111A interaction domains

**Figure 4:**
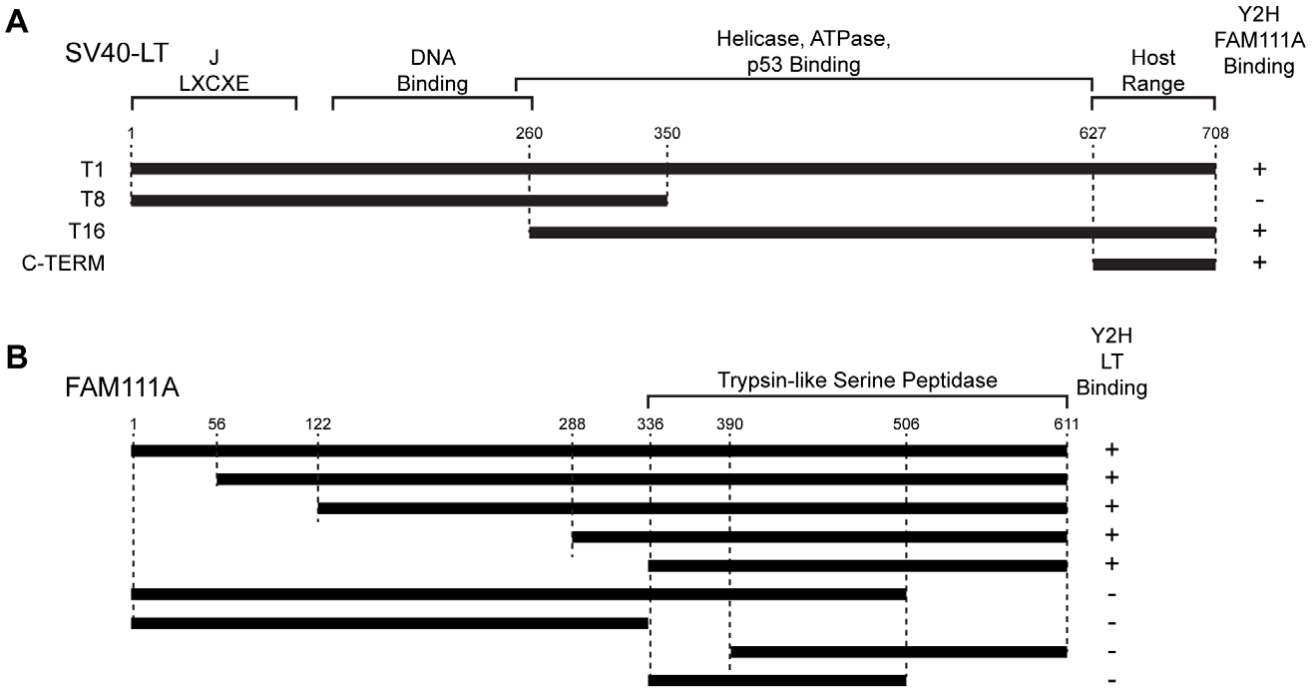
Mapping the LT and FAM111A interacting domains. (A) LT fragments were tested for binding to full-length FAM111A by yeast-two-hybrid (Y2H) in pairwise fashion. (B) Fragments of FAM111A were tested in pair wise Y2H analysis with full-length LT (T1). Numbers indicate residue position in human FAM111A.

We tested FAM111A binding to LT in a yeast two-hybrid (Y2H) assay. The LT constructs T1, T16 and C-TERM bound to FAM111A either as bait or prey in Y2H, while T8 could not (Figure 4A) consistent with the mass spectrometry analyses. To determine where LT bound to FAM111A, we generated fifty N- and C-terminal deletion constructs of FAM111A and tested them as bait or prey by Y2H against full-length LT (Figure S4 in Text S1). The C-terminal half of FAM111A (residues 336 to 611) was necessary and sufficient for interaction with LT (Figure 4B). This region of FAM111A contains a trypsin-like serine peptidase domain including the conserved catalytic triad of histidine, aspartate, and serine residues [27].

### LT specifically associates with FAM111A

**Figure 5:**
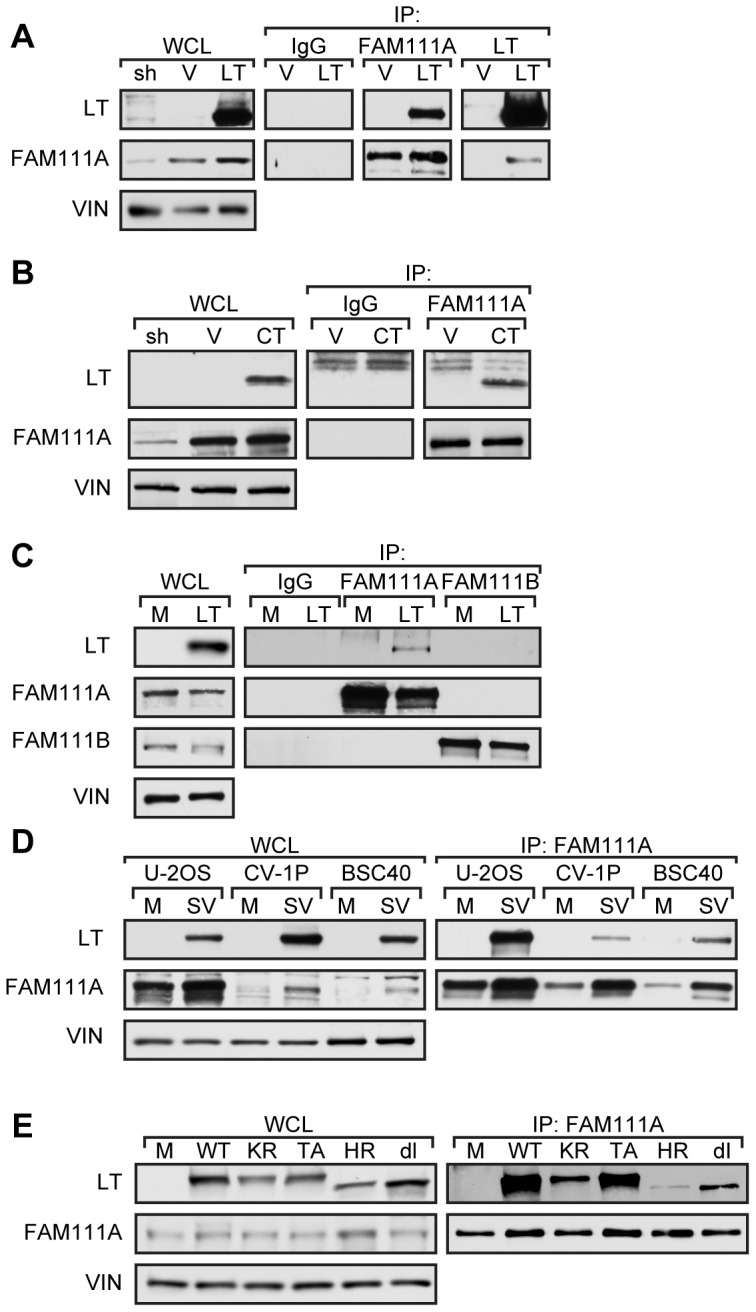
FAM111A is an SV40 LT binding protein. (A) Immunoprecipitations for FAM111A and LT with lysates from U-2 OS cells stably expressing full-length LT (LT) or vector control (V). Whole cell lysate of the U-2 OS cell line stably expressing shRNA-2 (sh) against FAM111A was used as a control for FAM111A antibody specificity and normal rabbit serum (IgG) as an immunoprecipitation control. The indicated proteins were detected by western blot analysis. (B) Immunoprecipitations for FAM111A with lysates from U-2 OS cells stably expressing the LT C-terminus (CT) or vector control (V). (C) Immunoprecipitations of FAM111A and FAM111B in U-2 OS cells expressing LT or mock (M). Levels of FAM111A, FAM111B, LT, and vinculin (VIN) were determined by western blot. (D) Immunoprecipitations of FAM111A on U-2OS, BSC40, and CV-1P cells 48 hours post-infection with wild type SV40 (SV) or mock infected (M). (E) Immunoprecipitation of FAM111A in U-2 OS cells transfected with viral DNA encoding wild type SV40 (WT), K697R acetylation mutant (KR), T701A phosphorylation mutant (TA) and host range mutants HR684 (HR) and dl1066 (dl).

Homologs of FAM111A exist in several mammalian species including mouse, rat, and rhesus monkey. FAM111A is also highly similar to its paralog human FAM111B (Gene ID: 374393) with 43% identity in the C-terminal 330 residues encompassing the peptidase domain and trypsin-like catalytic triad. To confirm that LT could bind to FAM111A in human U-2 OS cells, we performed immunoprecipitations with antibodies specific for FAM111A or FAM111B. An antibody for FAM111A detected a 70 kDa band that was reduced upon shRNA-mediated knockdown of FAM111A (Figure 5A). An immunoprecipitation for LT co-precipitated FAM111A and the reciprocal immunoprecipitation for FAM111A co-precipitated LT (Figure 5A). FAM111A also co-precipitated the LT fragment C-TERM (Figure 5B). Given the similarity between FAM111A and FAM111B we tested if LT could bind to FAM111B. However, we were unable to detect co-precipitation of FAM111B by LT in U-2 OS cells (Figure 5C). This result is consistent with the MudPIT and iTRAQ analyses that only detected FAM111A and not FAM111B in association with LT.

To extend our observations to AGMK cells, we immunoprecipitated LT and FAM111A from lysates prepared from CV-1P, BSC40 and U-2 OS cells infected with wild type SV40 virus (Figure 5D). LT co-precipitated FAM111A from CV-1P and BSC40 cells as well as U-2 OS cells. Notably, expression of FAM111A was similar in CV-1P and BSC40 cells and LT was able bind to FAM111A in both cell types.

We next examined how LT C-terminal mutations affect binding to FAM111A. The SV40 point substitution mutants T701A and K697R show wild-type host range activity, while the SV40 host range mutants HR684 and dl1066 cannot produce plaques in CV-1P cells [9], [10]. FAM111A co-precipitated wild-type LT as well as T701A and K697R mutants from CV-1P cells, but binding to the host range mutant LT HR684 and dl1066 was substantially reduced (Figure 5E).

### FAM111A expression is cell cycle dependent

**Figure 6:**
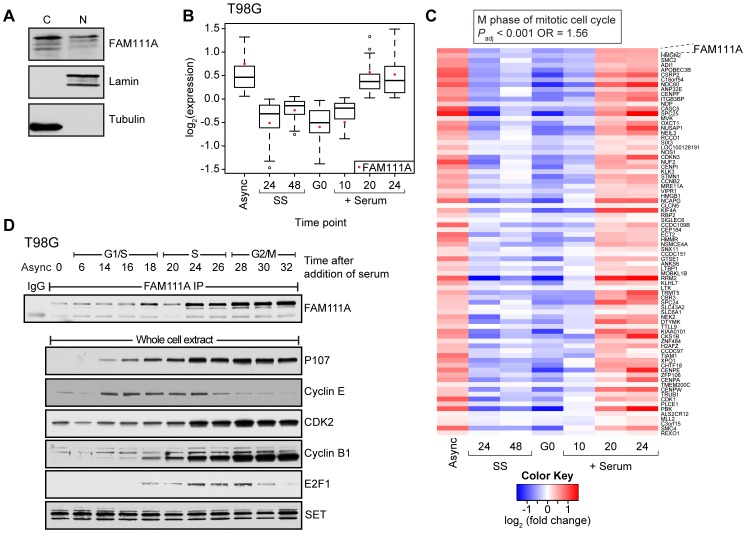
FAM111A expression is cell cycle dependent. (A) U-2 OS cells were fractionated and equal amounts of cytoplasmic (C) and nuclear (N) lysates were blotted with FAM111A antibodies. Tubulin and lamin serve as cytoplasmic and nuclear markers, respectively. (B) Box plot depicting the average expression of 79 genes in T98G cells with profiles similar to that of *FAM111A* (Pearson correlation coefficient R>0.9). FAM111A expression is denoted by the red dot. (C) RNA was extracted from asynchronously (Async) growing T98G cells or that were serum starved (SS) for 24, 48 or 72 (G0) hours then stimulated to enter the cell cycle by addition of serum for indicated hours. Heatmap depicting the expression of genes with expression pattern was similar to FAM111A. (D) Whole cell lysates prepared from T98G cells at the indicated hours after serum starvation and release were immunoprecipitated with FAM111A antibodies and immunoblotted with FAM111A antibodies. Negative control included immunoprecipitation with normal rabbit serum (IgG) from asynchronous T98G cells. The whole cell lysates that were used for immunoprecipitations were also probed with the indicated antibodies to mark cell cycle progression. Time points after release form G0 and cell cycle stage are depicted.

We sought to characterize FAM111A expression. Differential cellular extraction revealed that FAM111A was present in the nuclear and cytoplasmic fractions of U-2 OS cells (Figure 6A). Prior work revealed that the *FAM111A* promoter was bound by the DREAM complex in G0 or quiescent T98G cells [14]. Given that expression of DREAM target genes are regulated in a cell cycle-dependent manner, we examined mRNA expression profiles of cell cycle synchronized T98G cells. FAM111A levels were reduced in serum-starved G0 cells and increased 20 hr after serum addition when cells were enriched for S phase (Figure 6B and Figure S5 in Text S1). We identified 79 genes that exhibited cell cycle expression profiles similar to that of *FAM111A* (Pearson correlation coefficient R>0.9). This FAM111A gene set was significantly enriched for the GO term “M phase of mitotic cell cycle” (Figure 6C).

Similar to the mRNA levels, FAM111A protein levels were lowest in serum starved T98G cells and increased as cells progressed towards the G2/M phase of the cell cycle (Figure 6D). The pattern of FAM111A protein expression more closely resembles the expression patterns of late cell cycle genes such as CDK2 and Cyclin B1 than early cell cycle genes such as E2F1, Cyclin E and p107 (Figure 6D).

### FAM111A knockdown rescues the host range restrictive function

**Figure 7:**
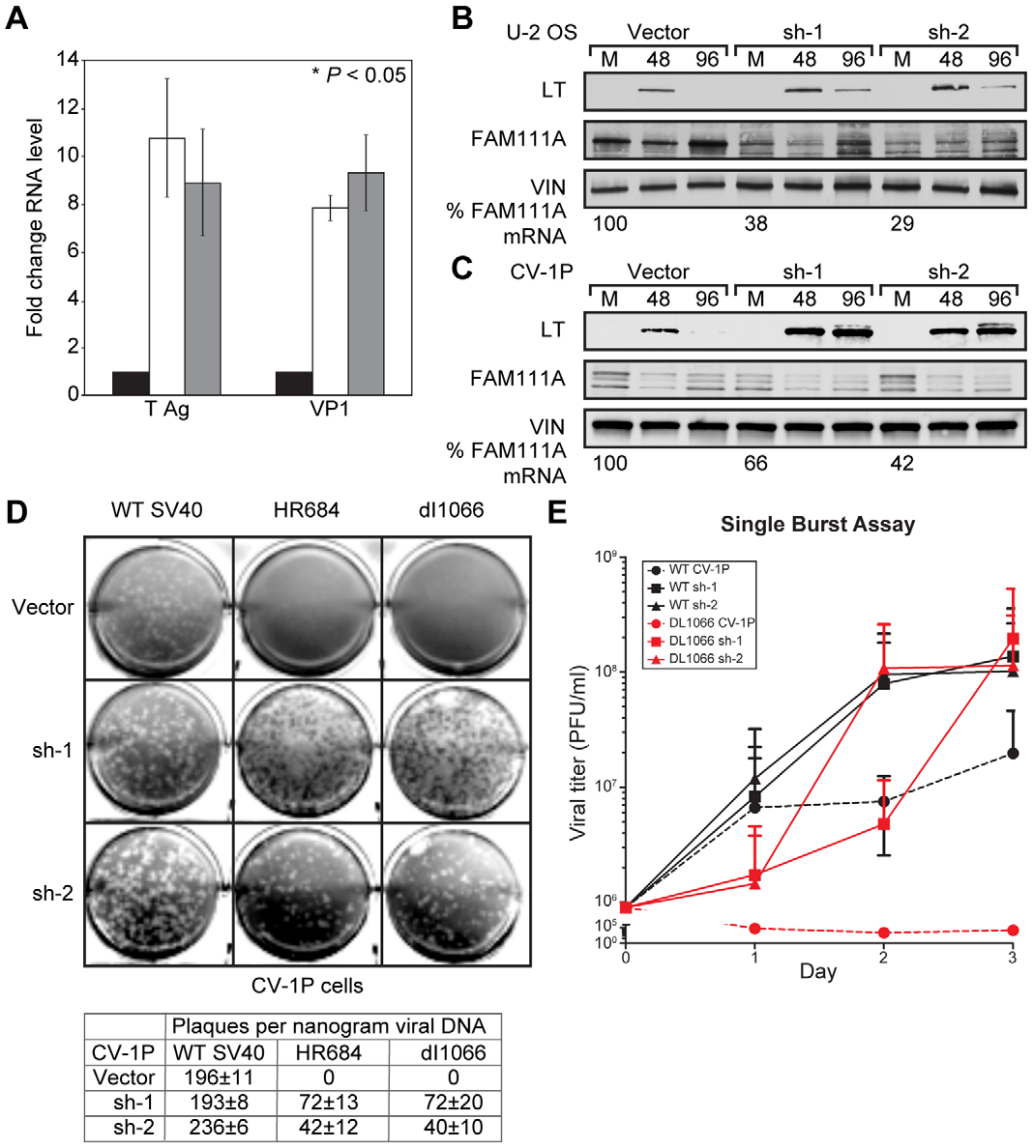
Depletion of FAM111A increases viral gene expression and renders CV-1P cells permissive for host range mutant viruses. (A) U-2 OS cells were co-transfected with host range viral DNA (HR684) and control siRNA (black bars), siRNA targeting FAM111A (white bars) or an expression vector for the C-terminus of LT (grey bars). Quantitative RT-PCR was performed 72 hours post-transfection to determine the expression levels of LT, VP1 and FAM111A (latter not shown) mRNA relative to actin. Error bars represent standard deviation from the mean. U-2 OS (B) and CV-1P (C) cell lines stably expressing two different shRNAs against FAM111A or vector control were generated and the amount of FAM111A RNA remaining (% FAM111A RNA) was confirmed by quantitative RT-PCR. Viral DNA encoding HR684 was transfected into the indicated cell lines and whole cell lysates were harvested at 48 and 96 hours post transfection. (D). CV-1P cells stably expressing two different shRNAs against FAM111A or vector control were transfected with viral DNA and assayed for lytic infection by plaque assay. Plaques were counted 8 days after transfection. Results shown are the average of three independent experiments with standard deviation from the mean denoted by +/−. (E) Control or FAM111A shRNA depleted CV-1P cells were infected at a multiplicity of infection of three with either wild-type SV40 virus or the host range mutant dl1066 virus. Cells were freeze thawed at the indicated time points to extract virus and the viral titer was determined in BSC40 cells. Results shown are the average of three independent experiments with standard deviation from the mean indicated.

Binding of LT to p53 and RB serves to inactivate their growth suppressing functions. By analogy, LT binding to FAM111A might serve to inactivate the host range restriction function of FAM111A, thereby promoting increased and sustained viral gene expression. If so, then expression of the SV40 LT C-terminal region should have the same effect on virus replication as reduced FAM111A expression. Cells expressing LT C-TERM showed eight-to-ten fold increases of early (LT) and late (VP1) viral transcripts from the HR684 viral DNA relative to cells without LT C-TERM (Figure 7A). Knockdown of FAM111A also resulted in an eight-to-ten-fold increase in early (LT) and late (VP1) viral mRNA expression compared to non-targeting siRNA controls (Figure 7A).

To evaluate longer-term effects of FAM111A knockdown on viral gene expression, we generated U-2 OS and CV-1P cell lines stably expressing two different shRNAs specific (sh-1 or sh-2) for FAM111A or vector control. The reduction in FAM111A mRNA and protein expression mediated by sh-2 was slightly more effective than sh-1 in both human and monkey cells (Figures 7B and 7C). In control cells transfected with HR684 viral DNA, LT expression was detectable 48 hours after transfection but was markedly reduced by 96 hours. In contrast, LT expression persisted for 96 hours after transfection in U-2 OS (Figure 7B) and CV-1P (Figure 7C) cells depleted of FAM111A by sh-1 or sh-2. Decreased FAM111A levels results in sustained host range mutant viral gene expression consistent with the effects of expression of the LT C-terminal region (Figure 1B).

We examined the effects of depletion of FAM111A on lytic infection by host range mutant virus. DNA corresponding to wild-type SV40 or host range mutants HR684 and dl1066 was transfected into CV-1P cells expressing shRNAs targeting FAM111A. Wild-type SV40 was capable of inducing plaque formation in control CV-1P cells and in cells containing sh-1 or sh-2 against FAM111A (Figure 7D). Although the relative number of plaques produced by wild-type SV40 was not markedly affected by depletion of FAM111A, the size of the plaques were consistently larger and appeared more rapidly than in the vector control cell line (Figure 7D and data not shown). The two host range mutant viruses could not induce lytic infection in the CV-1P vector control cell line (Figure 7D), but could form plaques in the two FAM111A-depleted CV-1P cell lines.

A single burst assay quantified the effect of FAM111A depletion on virus yield in restrictive CV-1P cells. Cells expressing shRNAs targeting FAM111A or vector control were infected with wild-type SV40 or host range mutant virus, dl1066, at a multiplicity of infection (MOI) of 3. Virions were harvested at several intervals and quantified by plaque assay in permissive BSC40 cells. The wild-type SV40 virus yield was similar in the presence or absence of FAM111A. In contrast, the host range virus yield was negligible in control CV-1P cells but was comparable to wild-type SV40 virus yield when FAM111A was depleted with either sh-1 or sh-2 (Figure 7E).

### FAM111A depletion rescues adenovirus growth in restrictive cells

**Figure 8:**
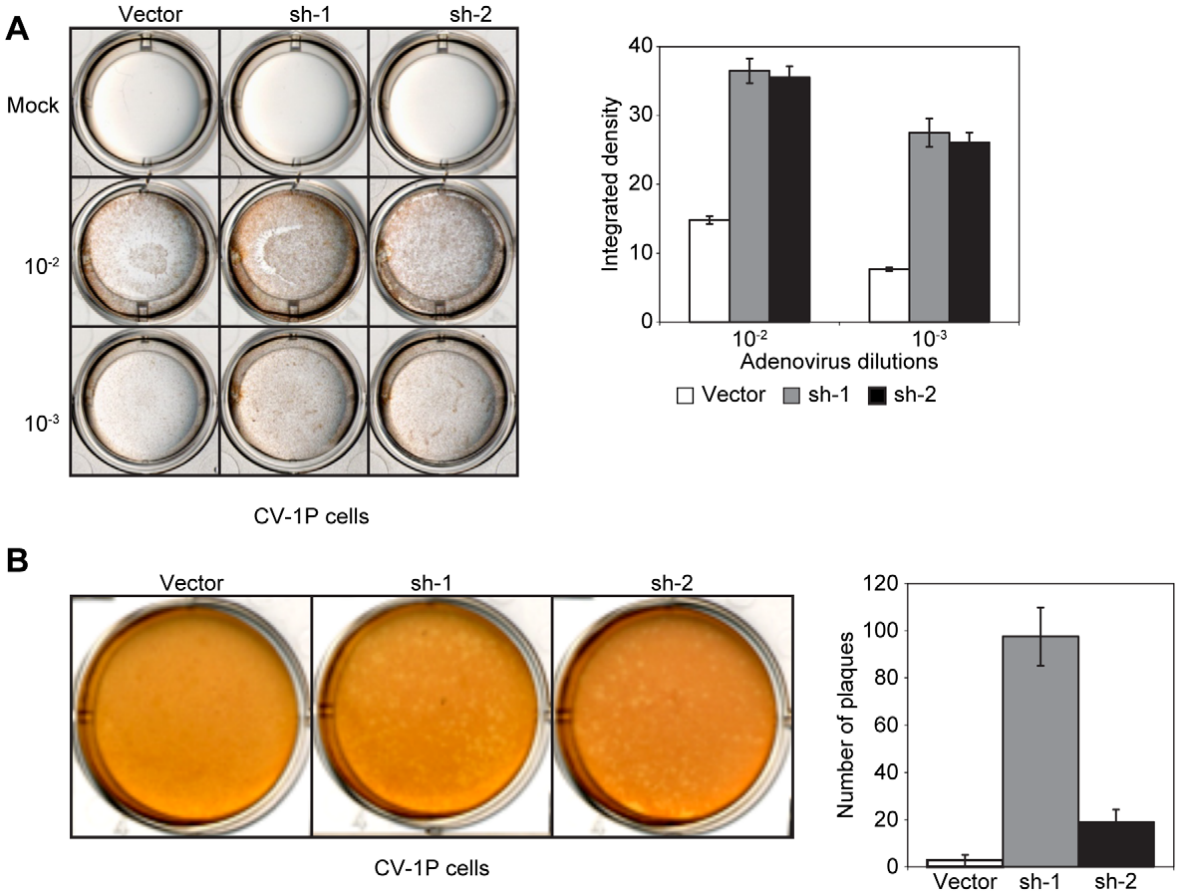
Depletion of FAM111A renders CV-1P cells permissive for Adenovirus 5 infection. (A) CV-1P cell lines stably expressing shRNA against FAM111A or vector control were mock infected or infected with Ad5 at the indicated dilutions. Ad5 infected cells were visualized using Adeno-X Rapid Titer (hexon protein, brown color). Results from a representative experiment, and quantification of integrated density across two biological replicates are shown (B). CV-1P cells stably expressing two different shRNAs against FAM111A or vector control were infected with Ad5 and assayed for lytic infection by plaque assay. Results from a representative experiment and a graph showing the average number of plaques in three biological replicates are shown.

AGMK cells can support human adenovirus replication only when co-infected with SV40 [28]. It has been long recognized that the C-terminal region of LT contains a helper function that permits human adenovirus infection of monkey cells [12], [29]. Since depletion of FAM111A or expression of the C-terminal region of LT can overcome the host range restriction in CV-1P cells, we investigated the role of FAM111A in adenovirus infection. Knockdown of FAM111A supported Adenovirus 5 (Ad5) replication as measured by increased amounts of the adenoviral hexon protein in FAM111A-depleted CV-1P cells (Figure 8A). Infection with Ad5 led to plaque formation in the FAM111A-depleted but not in the control CV-1P cell lines (Figure 8B). These results indicate a critical role for FAM111A in restriction of SV40 and adenovirus replication.

## Discussion

The propensity of viruses to replicate in host cells depends on their ability to manipulate key host defenses. The multifunctional SV40 LT viral protein encodes discrete domains required for viral replication including origin DNA binding, helicase activity, and the ability to hijack critical host proteins. The LT C-TERM domain is necessary for evading host range restriction in AGMK cells. We demonstrate that the host protein, FAM111A, plays a critical role in restricting viral replication, and that the LT C-terminal region binds to FAM111A to overcome this effect.

**Figure 9:**
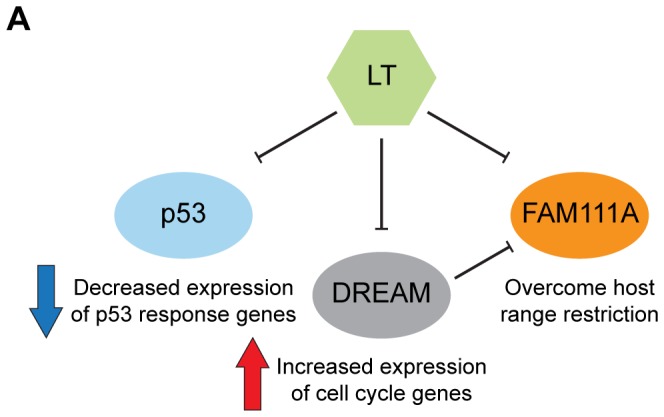
LT interactome perturbs host gene expression, overcomes host range restriction and enables viral gene expression. SV40 LT interaction with p130 (RBL2) disrupts the DREAM complex leading to cell cycle entry and increased FAM111A expression. LT reduces the expression of p53-responsive genes. LT binding to FAM111A overcomes SV40 host range restriction and enables the adenovirus helper effect.

Proteomic identification of LT associated proteins confirmed several known co-complex associations including p53 and RB (Figure 9). LT bound to all three members of the RB family of proteins. In contrast, LT was unable to bind to any of the MuvB subunit proteins (LIN9, LIN37, LIN54, LIN52, or RBBP4), indicating that LT can disrupt the p130-containing DREAM complex. In keeping with the ability to disrupt the DREAM complex, LT led to increased expression of DREAM target genes (Figure 2B).

Most intriguingly, we identified an interaction of the LT C-terminal region with FAM111A and provided several lines of evidence that this interaction contributes to the host range function of LT. FAM111A binds specifically to LT, as demonstrated by two mass spectrometry approaches, Y2H analysis, and reciprocal co-immunoprecipitation. Furthermore, we demonstrated that expression of the LT C-terminal domain or depletion of FAM111A in restrictive CV-1P cells led to sustained viral gene expression and infectious virion formation by host range mutant SV40 viruses. This data strongly supports the model that FAM111A functions as a host range restriction factor that is specifically counteracted by binding to the C-terminal region of LT. In addition, FAM111A depletion enabled human Ad5 to replicate in AGMK cells consistent with the model that FAM111A contributes to viral host restriction.

The observation that host range mutant viruses can productively infect permissive BSC40 cells but not restrictive CV-1P cells suggests that these cells differ in a factor that determines cellular susceptibility to viral infection. However, levels of FAM111A were not appreciably different between CV-1P and BSC40 cells, and LT could bind to FAM111A in both cell types. It is possible that small differences in FAM111A levels or activity could affect viral replication or the efficiency of host range restriction. For example, we observed that *FAM111A* mRNA and protein levels are regulated in a cell cycle dependent manner with the lowest expression during G0 or the quiescent phase with peak expression during G2/M phase. It is possible that differences in the proliferation rate or the cell cycle dependent expression of FAM111A in BSC40 and CV-1P cells could account for the restrictive phenotype.

LT-mediated inhibition of FAM111A activity to promote viral replication is consistent with our observations that loss of FAM111A expression by RNAi-mediated knockdown rescues the host range phenotype. FAM111A is predicted to contain a trypsin-like serine peptidase domain. The conservation of the catalytic triad in the FAM111A primary sequence suggests that the protein may act as a specific peptidase. In a simple model, LT binding could inhibit the FAM111A peptidase activity. Although LT binds to the peptidase domain, there is no evidence that LT itself undergoes proteolysis or is a substrate of FAM111A and the exact role of FAM111A remains to be elucidated. It should be noted that several known LT-interacting proteins, including RB, p53, FBXW7 and CDC73, are *bona fide* tumor suppressors. The *FAM111A* locus has been recently associated with prostate cancer susceptibility in a subset of the Japanese population [30] raising the possibility that FAM111A may play a role in tumorigenesis.

## Materials and Methods

### Cells

BSC40 (gift from J. Pipas, University of Pittsburgh), CV-1P [31], T98G [32] and U-2 OS [33] cells were cultured in Dulbecco's modified Eagle's medium (DMEM) (Cellgro) supplemented with 10% Fetal Clone-I serum (HyClone), penicillin and streptomycin. Cells were transfected using Lipofectamine 2000 transfection reagent (Invitrogen) according to the manufacturer's protocol.

### Plasmids and viral DNA

SV40 genomic DNA (strain 776) was cloned into the BamH1 site of pBluescript KS (Stratagene) for propagation in bacteria. Wild type LT cDNA was transiently expressed from the pSG5 vector. The C-terminal fragment of LT was transiently expressed from the pVAX1 expression vector (Invitrogen). The C-TERM construct contained LT residues 627–708 in frame with an N-terminal hemagglutinin (HA) epitope tag (YPYDVPDYA) and the SV40 nuclear localization signal (NLS) (SPKKKRKVED) cloned into the pWZL retroviral vector [10], [34]. Full-length and truncated LT containing N-terminal HA and FLAG epitope tags were expressed from the pMSCV retroviral vector (gift from Matthew Sowa and Wade Harper [35]).

### RNAi

siRNA oligonucleotides were purchased from Dharmacon. Lentiviral vectors (pGIPZ) with shRNA directed against FAM111A were obtained from Open Biosystems. The sequences of siRNA and shRNA are provided in Supplementary experimental procedures in Text S1.

### Antibodies

The following antibodies were used: LT mouse monoclonal antibodies PAb419 and PAb901 [36]; HA mouse monoclonal antibody HA-11 (Covance); VP1 rabbit polyclonal antibody supplied by N. Christensen (Pennsylvania State University); FAM111A antibodies BL8623 and BL8624, FAM111B antibodies BL8627 and BL8630 and SET antibodies were generated by Bethyl Labs. Antibodies to p107, Cyclin E, CDK2, Cyclin B1 and E2F1 were obtained from Santa-Cruz Biotechnology. Tubulin and Lamin A/C antibodies were obtained from Cell Signaling. For affinity purification followed by mass spectrometry, an anti-HA affinity matrix (Pierce) was used in combination with the HA eluting peptide (Roche) and anti-FLAG beads (Sigma) were used in combination with 3XFLAG peptide (Sigma).

### Western blots

Whole cell lysates were prepared in EBC buffer (50 mM Tris-HCl [pH 8.0], 150 mM NaCl, 0.5% Nonidet P-40) supplemented with protease inhibitor cocktail set I (Calbiochem) and phosphatase inhibitor cocktail (Sigma). The Subcellular Protein Fractionation Kit for Cultured Cells was used for nuclear/cytoplasmic fractionation was used (Thermo Scientific). Membranes were blocked and incubated with the appropriate primary antibody in TBS-T overnight at 4°C. Detection of proteins was performed with horseradish peroxidase-conjugated secondary goat antibody (Pierce) in TBS-T and enhanced chemiluminescence (Pierce).

For immunoprecipitations, whole cell lysate was incubated with antibodies and protein A-Sepharose beads overnight at 4°C. Immune complexes were washed four times with EBC and boiled in sample buffer.

### MudPIT, and iTRAQ

See Supplementary Data.

### Yeast two-hybrid

Yeast two-hybrid matrix-style experiment with LT and FAM111A as bait or prey was essentially as previously described [37]–[40].

### Viral infection

Cells at 80% confluency were infected with wild type SV40 diluted in DMEM supplemented with 2% Fetal Clone-I serum (HyClone), penicillin and streptomycin for two hours. BSC40 and CV-1P were infected at a multiplicity of infection (MOI) of 0.125 and U-2 OS at MOI of 0.5. SV40 plaque assays were as previously described [41], [42] with additional details in Supplemental Data. For Ad5 infection, cells were seeded at 400,000 per well on 6 well plates and infected with serial dilutions of Ad5 diluted in DMEM containing 2% FBS for 2 hours. Plaque assays were as described for SV40. For detection of the hexon protein, cells infected with Ad5 were stained 48 or 72 hours after infection (Adeno-X rapid titer kit, Clontech). Cell images were quantified using ImageJ software.

### RNA isolation and microarray analysis

U-2 OS cells were transfected with control siRNA and total RNA was extracted using TRIzol (Invitrogen) and purified in RNeasy columns (Qiagen). RNA integrity was determined using a Bioanalyzer (Agilent). Gene expression was assayed using Human Genome U133 Plus 2.0 arrays (Affymetrix) in a single batch. Microarray intensities were normalized using robust multi-array averaging (RMA) through the affy package in R/Bioconductor. Differential expression was determined using the limma package [43]. The complete set of expression profiling microarray data can be accessed from the Gene Expression Omnibus (GEO) repository GSE40567.

To select genes for clustering, differential expression was tested between all pairwise comparisons and all genes whose expression changes were statistically significant in two or more comparisons were retained (p<0.05 after Benjamini-Hochberg correction for multiple testing). Next, all genes that were differentially expressed in any T1, T6, T8 or T16-expressing cells compared to the vector control cells were adjoined to the previous set of genes. This resulted in a final set of 430 unique HUGO gene symbols. The expression profile of each gene was determined by taking the median expression levels of all probesets annotated to that gene. All the profiles were mean-centered and scaled by the standard deviation before using the mclust package to cluster the genes [44]. Reverting to the original RMA-normalized data, the gplots package was used to visualize the heatmap of fold changes for each gene relative to vector control.

Previous microarray profiling of IMR90 normal human fibroblasts transduced with either GFP or SV40 LT was incorporated into the heatmap in the following way. Data from Human Gene 1.0 ST arrays (Affymetrix) was preprocessed as described [16]. The genes in each of the fourteen clusters that also had a corresponding probeset on the Human Gene 1.0 ST array were included in the heatmap. The IMR90 column on the heatmap (Figure 2B) shows the log of the fold change of the SV40 LT-transduced IMR90 cell lines relative to GFP. The three columns of fold changes (T1, T16, and IMR90) were hierarchically clustered and the dendrogram was constructed by optimal leaf ordering using the seriation package in Bioconductor. Functional enrichment was determined using FuncAssociate 2.0. Enrichment for custom gene sets was computed using Fisher's exact test, and p-values were corrected for multiple testing using the Benjamini-Hochberg method.

GSEA was run using the Java-based desktop application. Probesets were collapsed to gene symbols using median levels. Four combinations of parameters were tried for each run of GSEA: genes were ranked by either signal-to-noise ratio or by t-test, and the p-value was estimated by permuting either sample or gene labels. Only GSEA runs that resulted in significant p-values across all four parameter sets were retained for further interpretation. Therefore, although the enrichment score traces and p-values depicted in the figures correspond specifically to t-test ranking and gene set permutation, these gene sets were significant among all parameter combinations tried.

The DREAM and B-MYB/MuvB gene sets were extracted from [14], [15] and the p53 target gene set corresponds to the “V$P53_02” gene set in the Molecular Signatures Database (MSigDB).

## Supporting Information

Text S1 This file contains supplementary figures, tables, experimental procedures, and references.
(PDF)
