# Supplementary material for: Identification of FAM111A as an SV40 Host Range Restriction and Adenovirus Helper Factor
Source: PLoS Pathog. 2012 Oct 18;8(10):e1002949. doi: 10.1371/journal.ppat.1002949 (PMC3475652; doi:10.1371/journal.ppat.1002949)
Supplement: Text S1 — This file contains supplementary figures, tables, experimental procedures, and references. (PDF) [file ppat.1002949.s001.pdf]

Identification of FAM111A as an SV40 host range restriction and adenovirus helper factor

Debrah A. Fine #, Orit Rozenblatt-Rosen #, Megha Padi, Anna Korkhin, Robert L. James, Guillaume Adelmant, Rosa Yoon, Luxuan Guo, Christian Berrios, Ying Zhang, Michael A. Calderwood, Soundarapandian Velmurgan, Jingwei Cheng, Jarrod A. Marto, David E. Hill, Michael E. Cusick, Marc Vidal, Laurence Florens, Michael P. Washburn, Larisa Litovchick, James A. DeCaprio*

# These authors contributed equally to this work.

* E-mail: james_decaprio@dfci.harvard.edu

Text S1

Supplementary Information

1. Supplementary Figure Legends pp 2-3
   - Figure S1 p 4
   - Figure S2 p 5
   - Figure S3 p 6
   - Figure S4 p 7
   - Figure S5 p 8
2. Supplementary Table Legends p 9
   - Table S1 pp 10-18
   - Table S2 pp 19-20
   - Table S3 p 21
   - Table S4 pp 22-25
3. Supplementary experimental procedures pp 26-31
4. Supplementary references pp 32-33

Supplementary Figure legends

Figure S1. The C-terminal region of SV40 LT does not increase early viral gene expression in T98G and HeLa cells when expressed in *trans*.
T98G or HeLa cells were co-transfected with viral DNA encoding wild type SV40 (wtSV40) or host range mutant HR684 and either vector alone (pVAX) or HA-tagged LT C-terminal region (pVAX-T Ag 627-708). Ninety-six hours post-transfection, cell lysates were harvested and western blotted for LT, LT C-TERM (HA) and tubulin (loading control).

Figure S2. Expression of LT fragments in U-2 OS cells. Cell lysates generated from stable U-2 OS cell lines expressing the indicated LT fragments were immunoblotted with an anti-HA antibody.

Figure S3. Sequence coverage of FAM111A in MudPIT analysis.
The FAM111A peptides identified in five independent affinity purification-MudPIT experiments against full length LT (Table S3 in Text S1) are highlighted in bold. The residues of a conserved catalytic triad in the trypsin-like peptidase domain of FAM111A are shown in blue. The total number of FAM111A unique peptides identified was 44 and the number of FAM111A residues that were detected by MudPIT totaled 381, yielding 62.4% sequence coverage.

Figure S4. FAM111A fragments tested for binding to full-length LT by pairwise yeast-two-hybrid. Fragments of FAM111A were generated and tested for binding to full-length LT by pairwise yeast-two-hybrid (Y2H) testing. Fragments that were positive

for binding to LT are shown in black, while those that failed to bind LT are shown in white. FAM111A residues are indicated at top.

Figure S5. Cell cycle profiles of cells subjected to array analysis. Asynchronous T98G cells were serum starved for one (SS 24h), two (SS 48h) or three days (G0), and then stimulated to re-enter the cell cycle by addition of serum for 10, 20 and 24h. Cells were pulse-labeled with BrdU, harvested at the indicated time points and subjected to FACS analyses to determine their cell cycle profile using PI labeling of DNA and anti-BrdU staining as described in [1].

Figure S1

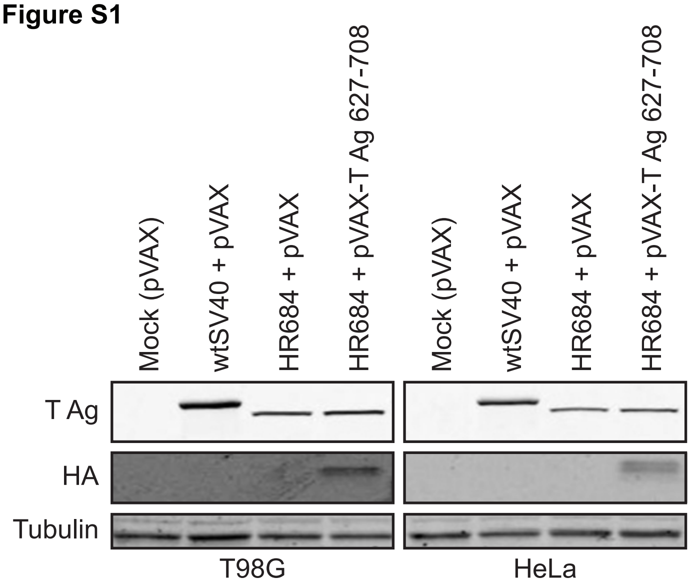
Mock (pVAX)
wtSV40 + pVAX
HR684 + pVAX
HR684 + pVAX-T Ag 627-708
Mock (pVAX)
wtSV40 + pVAX
HR684 + pVAX
HR684 + pVAX-T Ag 627-708
T Ag
HA
Tubulin
T98G
HeLa

Figure S2

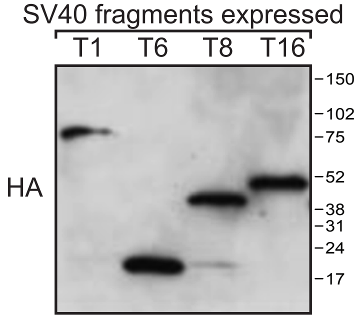
SV40 fragments expressed
T1
T6
T8
T16
HA
150
102
75
52
38
31
24
17

Figure S3

1 MSCKKQRSRK HSVNEKCNMK **IEHYFSPVSK EQQNNCSTSL MR**MESRGDPR ATTNTQAQRF
61 HSPK**KNPEDQ TMPQNR**TIYV TLKVNHRRNQ **DMKLKLTHSE NSSLYMALNT LQAVRKEIET**
121 **HQGQEMLVR**G TEGIKEYINL GMPLSCFPEG GQVVITFSQS KSKQKEDNHI FGRQDKASTE
181 CVK**FYIHAIG IGK**CKRRIVK CGKLHKKGR**K LCVYAFKGET IKDALCK**DGR **FLSFLENDDW**
241 **KLIENNDTIL ESTQPVDELE GRYFQVEVEK RMVPSAAASQ NPESEKRNTC VLREQIVAQY**
301 **PSLKR**ESEKI IENFKKKMK**V KNGETLFELH R**TTFGKVTKN SSSIKVVKLL VRLSDSVGYL
361 FWDSATTGYA TCFVFK**GLFI LTCRHVIDSI VGDGIEPSKW ATIIGQCVRV TFGYEELKDK**
421 **ETNYFFVEPW FEIHNEELDY AVLK**LK**ENGQ QVPMELYNGI TPVPLSGLIH IIGHPYGEKK**
481 **QIDACAVIPQ GQR**AKKCQER VQSK**KAESPE YVHMYTQR**SF QK**IVHNPDVI TYDTEFFFGA**
541 **SGSPVFDSKG SLVAMHAAGF AYTYQNETRS IIEFGSTMES ILLDIK**QRHK PWYEEVFVNQ
601 QDVEMMSDED L

Figure S4

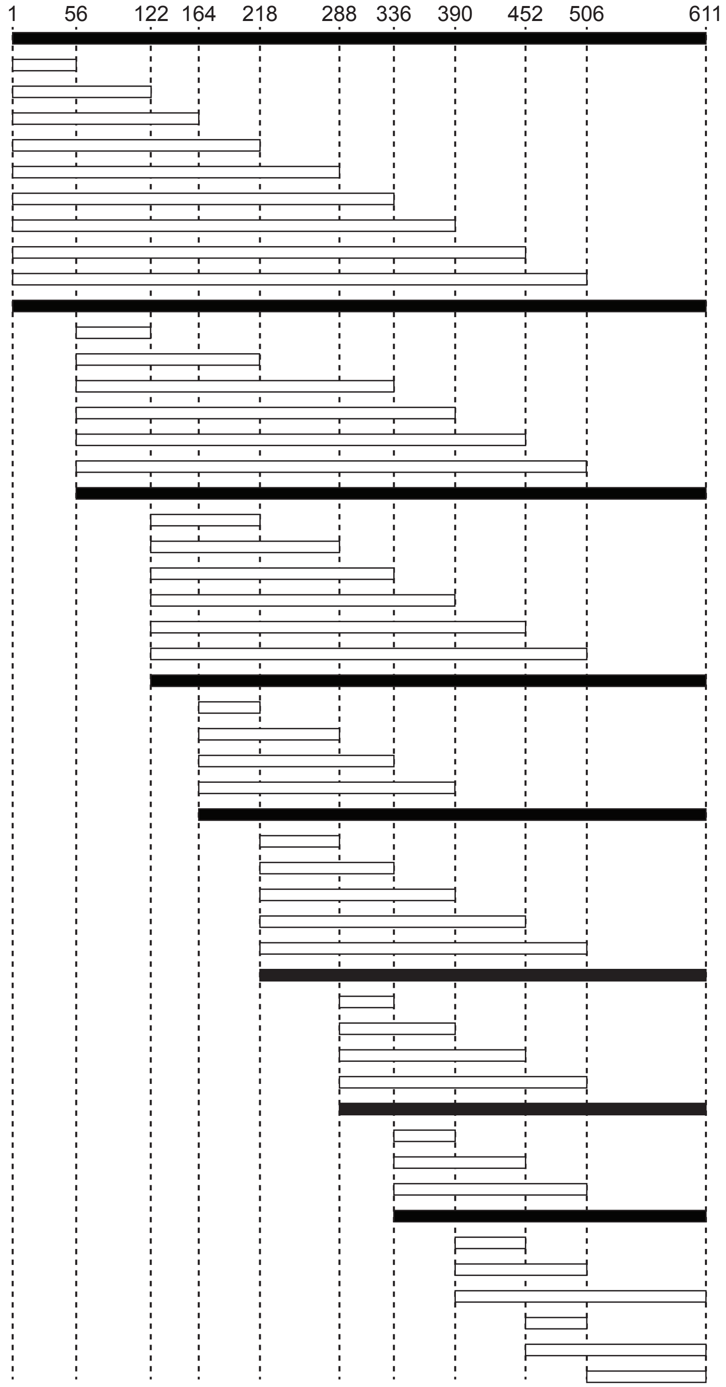

Figure S5

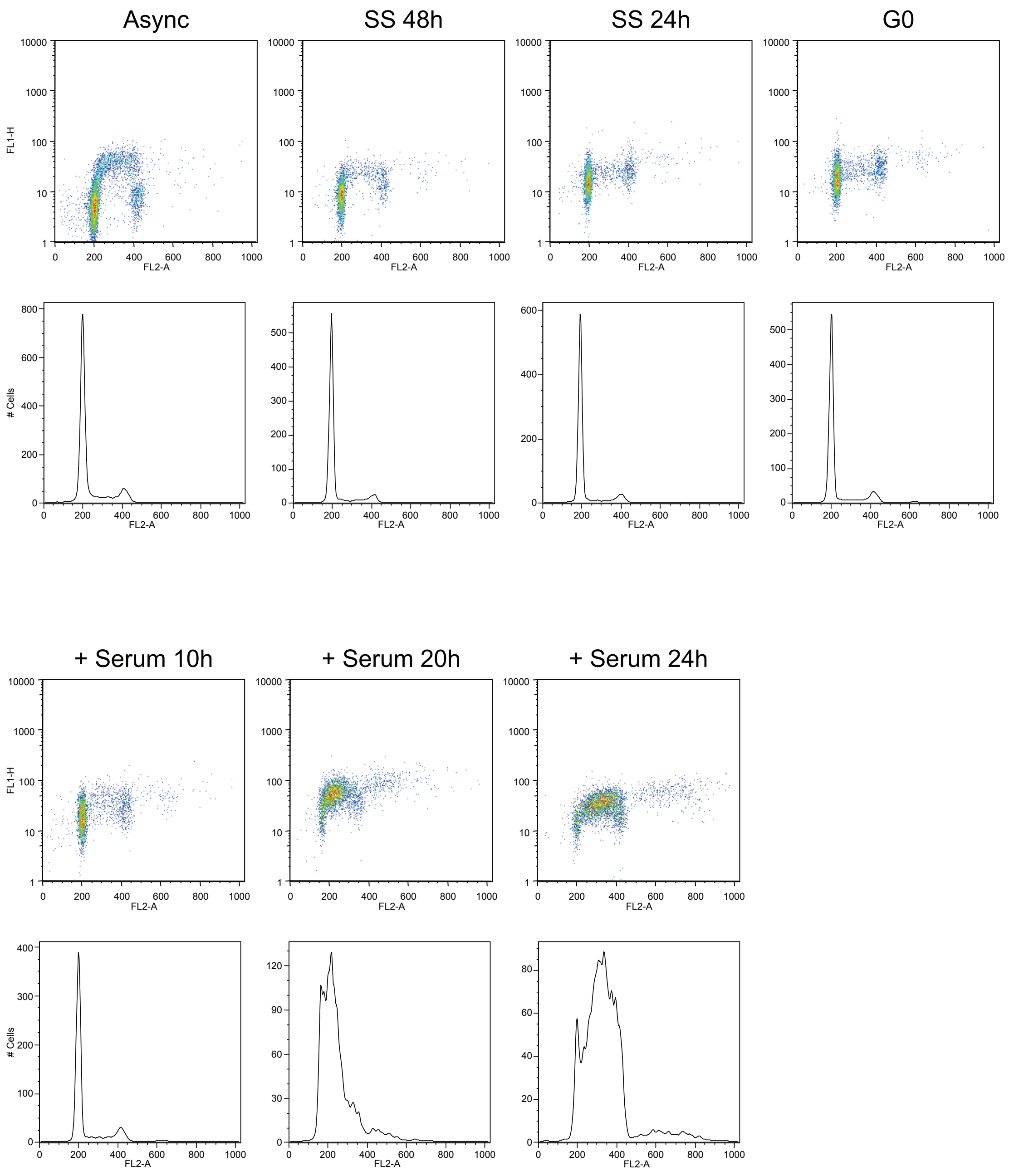
Async
SS 48h
SS 24h
G0
+ Serum 10h
+ Serum 20h
+ Serum 24h
FL1-H
FL2-A
Cells

Supplementary Table Legends

Table S1. List of all probesets (514), mapping to 430 unique genes, that were significant in two or more comparisons across conditions, or were differentially expressed by any fragment of SV40 LT. Cluster membership, adjusted p-values and fold changes are annotated for each probeset.

Table S2. List of all enriched GO terms for each cluster (Figure 2B), along with their adjusted p-values and log odds ratios.

Table S3. Summary of MudPIT proteomic analysis of SV40 LT-interacting proteins. Proteins are ranked according by the number of times detected in T1 IP (3 with FLAG antibody and 2 with HA antibody) samples and then by their relative enrichment over control in T1 Flag IP. The table shows proteins detected by more than 1 peptide and at least 2 times in T1 IP samples, with relative enrichment >25 over control IP samples. Viral proteins and fragments are highlighted in green. Known interactors (Viral Mint) are shown in blue color. dNSAF, Normalized Distributed Spectral Abundance Factor.

Table S4. iTRAQ-based quantitative analysis of full-length and sub-genomic fragments of SV40 LT. The numbers correspond to the relative enrichment factor measured for proteins detected in experimental and control immunoprecipitations.

Table S1. List of all probesets (514), mapping to 430 unique genes, that were significant in two or more comparisons across conditions, or were differentially expressed by any fragment of SV40 LT.

Probeset IDs	Gene symbols	Cluster number	T16 fold change	T16 adjusted p-value	T8 fold change	T8 adjusted p-value	T6 fold change	T6 adjusted p-value	T1 fold change	T1 adjusted p-value
1552658_a_at	NAV3	1	0.68786626	0.16302246	1.15503241	0.99994064	0.59030535	0.17604122	0.94041318	0.99129953
1553211_at	ANKFN1	1	0.82322955	0.64500336	1.31215062	0.99994064	0.7913384	0.75120643	1.01513153	0.99910253
201645_at	TNC	1	0.70171965	0.25071279	1.17731469	0.99994064	0.67425521	0.49520273	0.99757693	0.99916834
201862_s_at	LRRFIP1	1	0.70576836	0.18181035	1.14406313	0.99994064	0.71739189	0.52535502	1.2124848	0.86981297
202068_s_at	LDLR	1	0.83479855	0.56410899	1.27332439	0.99994064	0.78661703	0.63722448	1.16457213	0.89622263
202241_at	TRIB1	1	0.68420474	0.157134	1.21880444	0.99994064	0.74849608	0.6524608	1.14390454	0.95676743
202345_s_at	FABP5	1	0.79331551	0.19372646	1.14139647	0.99994064	0.81324766	0.6019607	1.11018492	0.92818473
202388_at	RGS2	1	0.87234255	0.73839645	1.43431974	0.99994064	0.72880259	0.50096101	1.18747735	0.8742763
202431_s_at	MYC	1	0.78512812	0.48359027	1.29887866	0.99994064	0.76040153	0.6740047	1.05809317	0.99129953
202679_at	NPC1	1	0.66748161	0.09967585	1.15599354	0.99994064	0.62409317	0.21905561	0.87887709	0.95676743
202819_s_at	TCEB3	1	0.84919434	0.57253511	1.05562689	0.99994064	0.70798956	0.29888769	1.02545677	0.99848066
203476_at	TPBG	1	0.77421169	0.3619362	1.17156202	0.99994064	0.74794288	0.5836006	0.99877619	0.99929199
203665_at	HMOX1	1	0.48136395	0.03538239	0.98878813	0.99994064	0.47515386	0.19645273	0.72294944	0.83581582
204420_at	FOSL1	1	0.86296064	0.85687936	1.2815887	0.99994064	0.58897025	0.49714557	1.13471763	0.98287233
205681_at	BCL2A1	1	0.61108007	0.54162275	1.0554074	0.99994064	0.28310321	0.110717	0.84764573	0.98882635
208394_x_at	ESM1	1	0.7927475	0.543812	1.45508154	0.99994064	0.54198819	0.110717	0.81411246	0.86981297
209101_at	CTGF	1	0.7728891	0.60566825	1.44356226	0.99994064	0.66557201	0.58806687	0.95698397	0.99758835
210090_at	ARC	1	0.82355296	0.51344643	1.36059089	0.99994064	0.89523189	0.88813484	1.01330372	0.99872331
210517_s_at	AKAP12	1	0.89885896	0.81941717	1.41784517	0.99994064	0.90751916	0.92440563	1.27647622	0.74574091
211509_s_at	RTN4	1	0.7713727	0.26384732	1.11414217	0.99994064	0.73310919	0.42282677	1.0180911	0.99854911
212445_s_at	NEDD4L	1	0.62852121	0.04338707	1.15164814	0.99994064	0.65894654	0.35997525	0.90467196	0.97742695
212724_at	RND3	1	0.72226492	0.06029324	1.22892766	0.99994064	0.78963983	0.57947918	1.07833093	0.97539088
213524_s_at	G0S2	1	0.59659895	0.4442177	1.3103934	0.99994064	0.4893256	0.50096101	1.22411011	0.97839333
214629_x_at	RTN4	1	0.80485455	0.32686572	1.13322421	0.99994064	0.74898047	0.41827506	1.07967717	0.97492246
217127_at	CTH	1	0.70453014	0.05217332	0.97472467	0.99994064	0.65786646	0.14586848	0.97063447	0.99562941
218001_at	MRPS2	1	0.8934961	0.77848806	1.25454713	0.99994064	0.74317129	0.50096101	1.07075865	0.9847175
218123_at	C21orf59	1	0.82322955	0.46080256	1.10310236	0.99994064	0.7040257	0.29888769	1.00518891	0.99910253
218140_x_at	SRPRB	1	0.88693483	0.74057687	1.05392095	0.99994064	0.70016491	0.29888769	1.12789544	0.93636054
218590_at	C10orf2	1	0.86222323	0.64704867	1.0824247	0.99994064	0.71279873	0.34401956	1.03686878	0.99418131
218647_s_at	YRDC	1	0.90389891	0.81556446	1.23179883	0.99994064	0.81086482	0.72697858	1.19609612	0.84717075
219557_s_at	NRIP3	1	0.62990231	0.0190581	1.11940596	0.99994064	0.57457482	0.08957542	0.89570775	0.96238473
219940_s_at	PCID2	1	0.79049779	0.25870099	1.10345924	0.99994064	0.77171141	0.50096101	0.94701991	0.98882635
221107_at	CHRNA9	1	0.79121042	0.69666303	1.50562936	0.99994064	0.73681108	0.75639549	1.38873051	0.80837584
222532_at	SRPRB	1	0.81443229	0.36686255	1.0027301	0.99994064	0.6882637	0.17604122	0.96466505	0.99361001
222900_at	NRIP3	1	0.64461068	0.14274157	1.12352586	0.99994064	0.62193397	0.37889109	0.83584075	0.93094177
223076_s_at	NSUN2	1	0.90500646	0.75259623	1.23399225	0.99994064	0.78480166	0.50096101	1.10442848	0.94399636
223687_s_at	LY6K	1	0.8191881	0.38607367	1.30631244	0.99994064	0.78480166	0.54173099	1.17002012	0.83580052
224480_s_at	AGPAT9	1	0.68750082	0.37171391	1.1048879	0.99994064	0.54228882	0.27971563	1.05574884	0.99469897
225524_at	ANTXR2	1	0.57164841	0.07601476	1.11414217	0.99994064	0.53835635	0.21905561	0.9015837	0.98814177
227638_at	EPG5	1	0.70280677	0.08977552	1.11154524	0.99994064	0.71453009	0.41827506	0.84483021	0.86700302
229257_at	TNRC18	1	0.75509671	0.40201379	1.04507031	0.99994064	0.59087847	0.19645273	0.9323434	0.99101785
229491_at	NHEDC2	1	0.85498242	0.49678386	1.16185766	0.99994064	0.84136284	0.69216094	1.02264116	0.99793329
229582_at	INO80C	1	0.76537216	0.27387553	1.20419303	0.99994064	0.796861	0.68379087	1.16869624	0.89391198
230112_at	MARCH4	1	0.61170162	0.11855545	1.10376522	0.99994064	0.52181127	0.17604122	0.81739198	0.9252345
230574_at	LOC1001309	1	0.63651511	0.06199824	1.07706103	0.99994064	0.66495717	0.40779008	0.91163823	0.9817172
231056_at	LOC339352	1	0.94540212	0.90174561	1.33687694	0.99994064	0.904066	0.88061722	1.06434559	0.9831982
1552682_a_at	CASC5	2	1.22032605	0.34939656	0.94936406	0.99994064	0.79527919	0.55155712	1.2562875	0.53308188
1556059_s_at	SPEN	2	1.17223894	0.66800855	1.06068774	0.99994064	0.73366837	0.50096101	1.21377415	0.82491264
201663_s_at	SMC4	2	1.36736651	0.0149264	1.00289229	0.99994064	0.90075084	0.8174319	1.25402548	0.30407719
201664_at	SMC4	2	1.20241368	0.37594999	0.97456704	0.99994064	0.86250218	0.75213142	1.19969416	0.71792788
201890_at	RRM2	2	1.29250214	0.23635339	0.94115223	0.99994064	0.77149747	0.53700513	1.17291625	0.85533473
202330_s_at	UNG	2	1.25538801	0.25065175	1.00884183	0.99994064	0.82492412	0.6740047	1.14633867	0.87045273
203418_at	CCNA2	2	1.15034505	0.5803117	1.03158784	0.99994064	0.84701925	0.72335067	1.17443484	0.80267533
203625_x_at	SKP2	2	1.20564068	0.49438895	0.99311544	0.99994064	0.82536262	0.73149453	1.18287695	0.83836884
203976_s_at	CHAF1A	2	1.19171011	0.36301909	1.10493895	0.99994064	0.77986699	0.40779008	1.2787492	0.28381348
204033_at	TRIP13	2	1.13393138	0.68362836	1.16883126	0.99994064	0.8055429	0.61548491	1.2614357	0.5530008
204817_at	ESPL1	2	1.16368453	0.56677644	1.093005	0.99994064	0.83514581	0.69997894	1.17843049	0.80915772
206109_at	FUT1	2	1.20305283	0.58807313	1.08917316	0.99994064	0.7494998	0.5738508	1.10867256	0.96797439
209408_at	KIF2C	2	1.33576542	0.05276442	1.13442927	0.99994064	0.95024186	0.95707007	1.34037189	0.13014897
213007_at	FANCI	2	1.26163973	0.22615742	0.95882095	0.99994064	0.91140656	0.88526989	1.24102599	0.58651993

Table S1. List of all probesets (514), mapping to 430 unique genes, that were significant in two or more comparisons across conditions, or were differentially expressed by any fragment of SV40 LT.

214988_s_at	SON	2	1.22566666	0.24980286	1.23091687	0.99994064	0.91912522	0.88480702	1.26590283	0.35053009
215942_s_at	GTSE1	2	1.31230221	0.1384509	1.04789924	0.99994064	0.8312379	0.69779279	1.18427161	0.80396464
217946_s_at	SAE1	2	1.25312761	0.15656251	1.12946012	0.99994064	0.89593543	0.81870906	1.22148262	0.53308188
218355_at	KIF4A	2	1.28395977	0.13430795	1.06051621	0.99994064	0.90770789	0.86695469	1.3246703	0.1948568
218662_s_at	NCAPG	2	1.19204057	0.44174629	0.97564847	0.99994064	0.86003464	0.75639549	1.29193486	0.36719317
218738_s_at	RNF138	2	1.14753116	0.64556544	1.17429917	0.99994064	0.8270807	0.69779279	1.19770007	0.78940222
218875_s_at	FBXO5	2	1.22793427	0.38469492	0.99840703	0.99994064	0.87308862	0.81594917	1.39348744	0.16243917
219004_s_at	MIS18A	2	1.12111427	0.65618942	1.08638339	0.99994064	0.83694226	0.63477584	1.21672236	0.56701162
219502_at	NEIL3	2	1.31096878	0.13954408	0.92840959	0.99994064	0.83291047	0.69779279	1.19507404	0.7842592
220651_s_at	MCM10	2	1.25538801	0.23082748	1.11411642	0.99994064	0.84436187	0.72335067	1.35256694	0.16585501
221520_s_at	CDCA8	2	1.13293624	0.6461209	1.00930812	0.99994064	0.82528634	0.63009894	1.22442126	0.61098324
221521_s_at	GINS2	2	1.28755435	0.09610297	0.948772	0.99994064	0.93865485	0.9306734	1.10170146	0.92818473
222039_at	KIF18B	2	1.14957453	0.60332916	1.06816412	0.99994064	0.75708822	0.40366842	1.20452694	0.73770186
222118_at	CENPN	2	1.24484547	0.31229852	0.95711665	0.99994064	0.88329464	0.83837504	1.23504762	0.66699811
223274_at	TCF19	2	1.32571133	0.05873283	0.99979208	0.99994064	0.89917054	0.8400929	1.05936518	0.9817172
223570_at	MCM10	2	1.21562647	0.28732558	0.9679023	0.99994064	0.86757878	0.75120643	1.16365764	0.80396464
224753_at	CDCA5	2	1.25701337	0.25763336	1.05482232	0.99994064	0.8225831	0.6740047	1.2635651	0.53308188
225777_at	C9orf140	2	1.45105278	0.00710495	1.16110625	0.99994064	1.02766259	0.98075082	1.29731906	0.242313
227165_at	SKA3	2	1.11391051	0.72314666	1.02685561	0.99994064	0.80569181	0.58501512	1.12893832	0.90041016
232291_at	MIR17HG	2	1.22736697	0.42326493	0.98445693	0.99994064	0.82364811	0.72375754	1.20280269	0.8083511
242939_at	TFDP1	2	1.12799969	0.72343175	0.97479224	0.99994064	0.74497642	0.41827506	1.16675367	0.86101918
1555609_a_at	ZMAT3	3	0.52329631	0.00564933	1.082925	0.99994064	1.04598827	0.98184097	0.5220042	0.01303608
1557765_at	LOC643401	3	0.28047939	0.00050995	0.67287014	0.99994064	1.17332282	0.93757504	0.33695628	0.0047244
1566147_a_at	LOC375010	3	0.49155792	0.00011668	0.87973038	0.99994064	0.91509917	0.91729106	0.42491122	1.51E-05
201235_s_at	BTG2	3	0.64400033	0.01055883	0.94356905	0.99994064	1.14290065	0.83446364	0.63454747	0.01902067
201236_s_at	BTG2	3	0.48956308	0.00361447	0.98039385	0.99994064	1.11326728	0.9394989	0.43745302	0.00165428
201939_at	PLK2	3	0.53589911	0.00075504	1.09086053	0.99994064	1.1695066	0.80940813	0.68910704	0.15639433
202284_s_at	CDKN1A	3	0.3430839	9.18E-07	1.17565655	0.99994064	1.0947489	0.91656777	0.37214412	2.91E-06
203409_at	DDB2	3	0.39068199	1.80E-06	1.00637408	0.99994064	1.30450276	0.50416832	0.30366797	1.21E-07
204566_at	PPM1D	3	0.60777111	0.02609775	1.0274964	0.99994064	1.12248798	0.91214032	0.62994597	0.11856564
204780_s_at	FAS	3	0.32391039	9.70E-05	1.12922528	0.99994064	1.10529643	0.95695498	0.2684577	1.63E-05
204781_s_at	FAS	3	0.52311498	0.01019474	1.10511768	0.99994064	1.13571433	0.91762799	0.39351758	0.00056209
205386_s_at	MDM2	3	0.76710714	0.20287493	1.03612639	0.99994064	1.14583555	0.82351062	0.78825446	0.59236026
207765_s_at	KIAA1539	3	0.74356632	0.05135767	0.98716741	0.99994064	1.0693247	0.93035352	0.79196029	0.41294141
207813_s_at	FDXR	3	0.46485951	0.00016372	1.05285007	0.99994064	1.19609612	0.78557149	0.34768836	4.41E-06
209375_at	XPC	3	0.67852161	0.04338707	1.00092462	0.99994064	0.98479818	0.99459195	0.64994971	0.05122457
209693_at	ASTN2	3	0.58761101	0.00775469	1.00770032	0.99994064	1.08869512	0.94292803	0.64517689	0.09364
213293_s_at	TRIM22	3	0.29658139	0.00033305	0.88425436	0.99994064	1.58092252	0.60419163	0.32326742	0.00140489
215407_s_at	ASTN2	3	0.64039473	0.00881885	0.87036956	0.99994064	1.11401346	0.87639373	0.66637214	0.04930218
215719_x_at	FAS	3	0.27071249	9.14E-05	1.0511972	0.99994064	1.05071155	0.98532298	0.20387169	9.87E-06
215785_s_at	CYFIP2	3	0.55997462	0.00032961	1.08515415	0.99994064	1.19430115	0.71532685	0.46580564	1.51E-05
216252_x_at	FAS	3	0.36514296	0.00074196	1.07561864	0.99994064	1.2004428	0.88111426	0.30042289	0.00013889
218007_s_at	RPS27L	3	0.34451372	7.77E-08	0.92564656	0.99994064	1.09000393	0.89279541	0.35386394	1.21E-07
218167_at	AMZ2	3	0.6799026	0.01096937	0.95612203	0.99994064	0.9821169	0.99067703	0.60809417	0.00202403
218288_s_at	CCDC90B	3	0.63881348	0.00436485	0.92325431	0.99994064	0.95398159	0.96523524	0.70066658	0.07427809
218346_s_at	SESN1	3	0.68373065	0.08406044	1.04666517	0.99994064	1.16529886	0.83837504	0.68640565	0.2348615
219099_at	C12orf5	3	0.5009019	3.85E-05	1.10379072	0.99994064	1.00688576	0.99808936	0.60965572	0.00254631
219628_at	ZMAT3	3	0.52811841	0.00033305	0.99663237	0.99994064	0.9255824	0.93736404	0.56864493	0.00207831
219655_at	C7orf10	3	0.42934223	0.01075968	1.18750479	0.99994064	1.41643723	0.7424078	0.41492812	0.01872288
220476_s_at	C1orf183	3	0.74960371	0.08211022	0.91545867	0.99994064	0.94158723	0.94762865	0.68119197	0.0255177
221577_x_at	GDF15	3	0.28216286	5.06E-06	1.36137702	0.99994064	1.11421939	0.93757056	0.20133455	2.56E-07
222487_s_at	RPS27L	3	0.37485382	7.50E-06	0.8320257	0.99994064	1.02996835	0.98636628	0.36960769	9.65E-06
223207_x_at	PHPT1	3	0.80949832	0.18386686	0.97119529	0.99994064	1.04591577	0.96108071	0.72940905	0.04482553
223272_s_at	NTPCR	3	0.59494711	0.00212897	0.93286055	0.99994064	1.03077777	0.98411725	0.6691029	0.05039434
223342_at	RRM2B	3	0.2516691	9.14E-05	0.90233393	0.99994064	1.21993138	0.88773889	0.23438194	5.41E-05
223443_s_at	AMZ2P1	3	0.70107142	0.09691905	0.9623943	0.99994064	1.02595444	0.98959636	0.61538734	0.02757496
225049_at	BLOC1S2	3	0.57257371	0.00109199	1.0109652	0.99994064	0.95266001	0.9688861	0.65648472	0.03363187
225160_x_at	MDM2	3	0.33547256	6.69E-05	1.0242018	0.99994064	1.00025419	0.99994393	0.38025347	0.00040466
225725_at	ZMAT3	3	0.73943744	0.27402111	0.98420676	0.99994064	1.20759219	0.79538955	0.70381427	0.38812756
225736_at	FBXO22	3	0.6126776	0.0024183	0.97319445	0.99994064	1.07100608	0.94323774	0.64048351	0.01319499
225737_s_at	FBXO22	3	0.58305343	0.00128562	0.92705917	0.99994064	1.06012423	0.95970439	0.64765616	0.02318917
226093_at	DCP1B	3	0.65152841	0.02441643	0.90955535	0.99994064	1.01522535	0.99508818	0.68715144	0.16585501
226813_at	NTPCR	3	0.67853729	0.00938955	0.90509011	0.99994064	1.08467788	0.90502788	0.75182389	0.22479956

Table S1. List of all probesets (514), mapping to 430 unique genes, that were significant in two or more comparisons across conditions, or were differentially expressed by any fragment of SV40 LT.

227221_at	ZMAT3	3	0.5593927	0.00724465	0.98318399	0.99994064	1.15178119	0.86985665	0.6032662	0.05555682
227522_at	CMBL	3	0.49493124	0.00710495	1.13736869	0.99994064	1.03821123	0.98724394	0.54186298	0.05402022
228315_at	ZMAT3	3	0.42631745	0.00027999	1.00837575	0.99994064	1.13073955	0.90505977	0.48727226	0.00267022
229711_s_at	MDM2	3	0.33431191	1.51E-06	1.06715273	0.99994064	1.15551287	0.84347422	0.29193788	3.32E-07
237737_at	LOC1002890	3	0.07888164	4.08E-11	0.98577707	0.99994064	1.06110444	0.97080669	0.07012633	1.78E-11
238097_at	FLJ41484	3	0.71651393	0.11855545	0.89705396	0.99994064	1.14591497	0.84885122	0.60169322	0.01458191
238127_at	FLJ41484	3	0.35451863	0.0007719	0.76723122	0.99994064	1.32988362	0.7840808	0.32474217	0.00057549
238935_at	RPS27L	3	0.29319519	1.26E-06	0.95793522	0.99994064	1.16680758	0.84453854	0.39813603	5.12E-05
241809_at	C1orf183	3	0.71861951	0.05135767	0.97576118	0.99994064	1.13511096	0.82840472	0.80414821	0.62771027
1552721_a_at	FGF1	4	0.6231422	0.03762752	1.02136604	0.99994064	0.89740637	0.91965715	0.9982917	0.99916834
1554667_s_at	METTL8	4	0.70470922	0.03369691	0.89500439	0.99994064	0.73160322	0.32424872	0.88709878	0.91493226
1557905_s_at	CD44	4	0.61148966	0.00245773	1.08809159	0.99994064	0.77369311	0.51148721	0.81428176	0.6976352
1564630_at	EDN1	4	0.56897349	0.00276306	0.90594791	0.99994064	0.77598464	0.62224015	0.77188973	0.62771027
201279_s_at	DAB2	4	0.63360985	0.00345822	0.97776974	0.99994064	0.74403033	0.36963292	0.91768229	0.96647351
201951_at	ALCAM	4	0.59975007	0.00566649	1.01715062	0.99994064	0.85676216	0.81585862	0.96810359	0.99615391
201952_at	ALCAM	4	0.62860835	0.00345822	1.07823128	0.99994064	0.91547983	0.90452792	1.0387871	0.99356659
202438_x_at	IDS	4	0.70355413	0.03672429	0.86934456	0.99994064	0.88349875	0.83764445	0.75425974	0.31782237
202439_s_at	IDS	4	0.71342483	0.01849495	0.93616409	0.99994064	0.75199762	0.29888769	0.88333546	0.87048307
203282_at	GBE1	4	0.73619848	0.04802943	0.99960729	0.99994064	0.84348442	0.69779279	0.88691433	0.89798574
203725_at	GADD45A	4	0.65718282	0.04293439	1.09469831	0.99994064	0.79594096	0.69870465	0.84418631	0.87512869
204490_s_at	CD44	4	0.58735311	0.0040887	1.1150435	0.99994064	0.78854591	0.65365784	0.84803752	0.86981297
204734_at	KRT15	4	0.50039294	0.01300898	1.11604871	0.99994064	0.63516354	0.49744506	0.91927389	0.99101785
204774_at	EVI2A	4	0.56974963	0.01075968	0.83767741	0.99994064	0.69081277	0.47406958	0.95598949	0.99418131
205082_s_at	AOX1	4	0.56658592	0.00110254	1.00702535	0.99994064	0.72988108	0.40779008	0.81761864	0.78136633
205083_at	AOX1	4	0.55586251	0.00152832	0.98671134	0.99994064	0.65715245	0.19645273	0.85947843	0.9004007
205542_at	STEAP1	4	0.61856624	0.00261966	1.06481293	0.99994064	0.8016809	0.60773706	0.98977099	0.99910253
205891_at	ADORA2B	4	0.64092761	0.00691206	0.98357025	0.99994064	0.76036639	0.48340148	0.85638613	0.85290017
206084_at	PTPRR	4	0.52311498	0.0016639	1.1226436	0.99994064	0.89044598	0.90060247	1.13398378	0.95590088
206248_at	PRKCE	4	0.6940605	0.0389236	1.02450948	0.99994064	0.78843661	0.6019607	0.97249765	0.99686393
206354_at	SLCO1B3	4	0.65195005	0.01849495	0.92048535	0.99994064	0.7460616	0.49303177	0.92220965	0.97795208
206421_s_at	SERPINB7	4	0.5558882	0.00014484	0.99034287	0.99994064	0.7073192	0.17604122	0.90991267	0.94832097
207303_at	PDE1C	4	0.69193096	0.01019474	0.91863692	0.99994064	0.77149747	0.40779008	0.8147899	0.57219548
207534_at	MAGEB1	4	0.64516198	0.03003241	0.99993069	0.99994064	0.70537711	0.41827506	0.81296586	0.80915772
208510_s_at	PPARG	4	0.71126874	0.02100802	0.99206049	0.99994064	0.86992725	0.7727152	0.92393718	0.96749566
209835_x_at	CD44	4	0.55650504	0.00467744	1.09081013	0.99994064	0.71638151	0.50096101	0.79981228	0.80396464
209890_at	TSPAN5	4	0.69916264	0.0489165	1.02207425	0.99994064	0.88834994	0.86059735	1.05789761	0.98882635
210675_s_at	PTPRR	4	0.51592503	0.0003275	1.10042946	0.99994064	0.84588494	0.78002719	0.96591401	0.99434695
210757_x_at	DAB2	4	0.7185697	0.04141319	0.94664801	0.99994064	0.76684133	0.42282677	0.91233359	0.95676743
210916_s_at	CD44	4	0.55543885	9.42E-05	1.02954008	0.99994064	0.77911057	0.41827506	0.73533149	0.09918882
210942_s_at	ST3GAL6	4	0.59556601	0.00467744	1.04736672	0.99994064	0.76233658	0.56985118	1.00178066	0.99916834
211203_s_at	CNTN1	4	0.52615751	9.70E-05	0.9428281	0.99994064	0.76073541	0.41827506	0.84498638	0.80396464
211668_s_at	PLAU	4	0.60355898	0.01790217	1.02906444	0.99994064	0.70306663	0.47406958	0.70568683	0.38812756
212014_x_at	CD44	4	0.53376132	0.00075504	1.07455054	0.99994064	0.72375166	0.41827506	0.7515981	0.48583782
214502_at	HIST1H2BJ	4	0.51014054	0.00128562	1.15420541	0.99994064	0.77794137	0.68476481	1.16115991	0.92818473
217790_s_at	SSR3	4	0.66880923	0.01669161	0.83512651	0.99994064	0.76856188	0.51126476	0.80264466	0.65695334
219553_at	NME7	4	0.6714724	0.00833384	0.92169841	0.99994064	0.84917472	0.73832423	0.88309058	0.89798574
219984_s_at	HRASLS	4	0.68031116	0.01538656	1.0087719	0.99994064	0.73933494	0.35997525	0.98511678	0.9986494
220663_at	IL1RAPL1	4	0.67820814	0.01961393	1.01891474	0.99994064	0.90285528	0.88061722	0.98157245	0.99848066
221211_s_at	C21orf7	4	0.42877717	0.00012637	0.94695427	0.99994064	0.71958316	0.52223646	0.82555334	0.86648081
221827_at	RBCK1	4	0.66825316	0.02966837	1.01288239	0.99994064	0.80794743	0.68954009	0.95367306	0.99129953
222745_s_at	C15orf29	4	0.52748428	0.01218007	1.44770401	0.99994064	0.78364202	0.77523075	1.03440417	0.99848066
222802_at	EDN1	4	0.7284154	0.1384509	1.2262615	0.99994064	0.88921242	0.87279104	1.1337218	0.93636054
222963_s_at	IL1RAPL1	4	0.6927948	0.02261801	1.13034774	0.99994064	0.96101665	0.97206523	0.91425383	0.96238473
223249_at	CLDN12	4	0.68317796	0.0489165	1.10035319	0.99994064	0.95738205	0.9732948	1.00261427	0.99910253
223952_x_at	DHRS9	4	0.72634825	0.01991279	1.03118272	0.99994064	0.83957629	0.64465978	0.90187538	0.91295879
225173_at	ARHGAP18	4	0.68374645	0.0438434	0.89856826	0.99994064	0.72988108	0.41827506	0.92231619	0.97839333
225387_at	TSPAN5	4	0.63560395	0.01960976	0.99833783	0.99994064	0.88765235	0.88062021	0.93232186	0.98589203
225647_s_at	CTSC	4	0.73006661	0.0424417	0.99755388	0.99994064	0.87288692	0.78429765	1.03161167	0.99418131
227209_at	CNTN1	4	0.43429081	0.00154067	0.78794491	0.99994064	0.63384412	0.42282677	0.81292829	0.90561205
227870_at	IGDCC4	4	0.49101307	0.00047589	0.95828941	0.99994064	0.78558176	0.68379087	0.96441991	0.99469897
233085_s_at	OBFC2A	4	0.60524871	0.00735511	1.0163519	0.99994064	0.7709629	0.60417944	0.98270707	0.99872331
1553055_a_at	SLFN5	5	0.47068485	0.0099936	0.82389554	0.99994064	0.7703041	0.79479193	0.48942736	0.03955441
1555339_at	RAP1A	5	0.62098629	0.02245441	0.56213946	0.10372894	0.6549394	0.27971563	0.63949279	0.0974251

Table S1. List of all probesets (514), mapping to 430 unique genes, that were significant in two or more comparisons across conditions, or were differentially expressed by any fragment of SV40 LT.

1555340_x_at	RAP1A	5	0.36493211	0.00033305	0.34251364	0.00194853	0.37746113	0.0209986	0.40381507	0.0017593
1557078_at	SLFN5	5	0.57938731	0.00431352	0.78333428	0.99994064	0.83447072	0.78002719	0.61779496	0.03072909
202180_s_at	MVP	5	0.66287098	0.02198092	0.91785193	0.99994064	1.00173437	0.99943583	0.69674372	0.14831327
202181_at	KIAA0247	5	0.79568354	0.32446418	1.05112434	0.99994064	0.95486367	0.97080669	0.67153446	0.05402022
202833_s_at	SERPINA1	5	0.77308556	0.43532996	0.80132904	0.99994064	0.93352895	0.96390387	0.54864062	0.01457555
202949_s_at	FHL2	5	0.57421649	0.05549729	0.89252635	0.99994064	1.0391952	0.98747912	0.56169804	0.11344308
203045_at	NINJ1	5	0.76570823	0.10920525	0.90100062	0.99994064	1.05175596	0.96108071	0.70534452	0.05039434
203331_s_at	INPP5D	5	0.75265815	0.17818442	0.9220179	0.99994064	1.19063675	0.75639549	0.7589447	0.46476315
204360_s_at	NAGLU	5	0.86196429	0.47182315	0.84576768	0.99994064	0.89314522	0.8106055	0.67466037	0.00659963
205219_s_at	GALK2	5	0.72777614	0.03121327	0.82243107	0.99994064	0.95134026	0.95907266	0.80385099	0.49389084
205383_s_at	ZBTB20	5	0.64623614	0.04349007	0.86727815	0.99994064	0.92267854	0.94986138	0.79888883	0.80267533
205563_at	KISS1	5	0.52528295	0.0394633	0.70849684	0.99994064	0.97344182	0.99406226	0.65651505	0.58651423
205756_s_at	F8	5	0.62025499	0.02250025	0.77645093	0.99994064	1.02299564	0.99165731	0.56189274	0.01095456
206665_s_at	BCL2L1	5	0.62495895	0.08581808	0.93465122	0.99994064	1.15479225	0.89227575	0.52747209	0.02285632
207065_at	KRT75	5	0.6232862	0.04293439	0.92926802	0.99994064	0.88767286	0.90714723	0.64782079	0.19255266
208478_s_at	BAX	5	0.39514845	0.00276306	0.84627591	0.99994064	0.80621321	0.86059735	0.35388029	0.00165428
208890_s_at	PLXNB2	5	0.76857964	0.28872346	1.10226161	0.99994064	0.99481489	0.99894139	0.60496909	0.0178457
209568_s_at	RGL1	5	0.70035906	0.04338707	0.72373494	0.99994064	0.72749034	0.36457601	0.71800543	0.18384229
210667_s_at	C21orf33	5	0.78911091	0.26588776	0.76763905	0.99994064	0.84629546	0.75213142	0.67313448	0.04482553
211810_s_at	GALC	5	0.68792984	0.03369691	0.79606971	0.99994064	0.86575657	0.80940813	0.72014866	0.1967672
211833_s_at	BAX	5	0.37278955	0.00279644	0.89287699	0.99994064	0.76891712	0.8296453	0.39145001	0.01009306
212312_at	BCL2L1	5	0.59843509	0.0125481	0.99548168	0.99994064	0.97632497	0.99092295	0.69277879	0.28849823
215037_s_at	BCL2L1	5	0.57486695	0.0489165	0.89065174	0.99994064	1.07455054	0.97080669	0.54155008	0.05761139
218309_at	CAMK2N1	5	0.61175816	0.04338707	0.81514766	0.99994064	0.77733048	0.73662468	0.60295964	0.09339883
218634_at	PHLDA3	5	0.60115128	0.00178027	0.92470601	0.99994064	0.98760086	0.99567542	0.51926156	0.00018239
222845_x_at	TMBIM4	5	0.79029691	0.13954408	0.78031759	0.99994064	0.86337945	0.73877098	0.70562162	0.02606408
223892_s_at	TMBIM4	5	0.82660309	0.3444135	0.76119254	0.99994064	0.87318949	0.7840808	0.69782314	0.03369634
228863_at	PCDH17	5	0.66965967	0.01880969	0.86110834	0.99994064	0.97621219	0.98731755	0.73473709	0.2572255
229163_at	CAMK2N1	5	0.56644194	0.02107353	0.7191344	0.99994064	0.77713295	0.74252312	0.7064699	0.56719061
232946_s_at	NADSYN1	5	0.68708794	0.04602249	0.85773268	0.99994064	1.06198741	0.96137944	0.63300991	0.02455428
241014_at	LOC339400	5	0.43253836	0.00961545	0.86555656	0.99994064	1.04090134	0.99044084	0.43165981	0.02285632
243999_at	SLFN5	5	0.55811462	0.00110254	0.7426735	0.99994064	0.82582042	0.73877098	0.62133074	0.0178457
1553151_at	ATP6V0D2	6	0.23080291	0.00305584	0.76406467	0.99994064	0.49749939	0.6019607	0.50642949	0.62771027
1553798_a_at	FBXL13	6	0.56587946	0.01297805	0.98096031	0.99994064	0.66986085	0.41827506	0.94820221	0.99361001
1555786_s_at	C14orf34	6	0.43400994	0.01297805	1.09421785	0.99994064	0.65380546	0.65965716	0.80234799	0.94358109
1556134_a_at	B3GNT5	6	0.60659268	0.01881862	1.04516689	0.99994064	0.85949829	0.85258586	1.00161865	0.99916834
1558846_at	PNLIPRP3	6	0.24934238	0.01297805	0.94413605	0.99994064	0.44331375	0.58229341	0.56484751	0.81339872
1568574_x_at	SPP1	6	0.46278051	0.02609775	0.77007276	0.99994064	0.48058605	0.21905561	0.67656479	0.78380439
203889_at	SCG5	6	0.42074946	0.01881862	1.16465286	0.99994064	0.72550963	0.80198493	0.78632629	0.94317383
204119_s_at	ADK	6	0.66187622	0.01888714	1.03996382	0.99994064	0.81654257	0.69779279	0.84966537	0.85330097
204298_s_at	LOX	6	0.55241842	0.00074196	0.93504001	0.99994064	0.74967299	0.48340148	0.80249631	0.69347287
204580_at	MMP12	6	0.54030028	0.00797215	1.27385406	0.99994064	0.85464667	0.86599454	0.99286307	0.99910253
205513_at	TCN1	6	0.51719014	0.00881885	0.95213189	0.99994064	0.87599832	0.91377244	0.79641925	0.86700302
205819_at	MARCO	6	0.51859013	0.00795317	1.03261324	0.99994064	0.7413534	0.68379087	0.8126466	0.88996314
206239_s_at	SPINK1	6	0.22627775	0.00298714	0.91535292	0.99994064	0.73825954	0.88766493	0.68360428	0.91547952
206561_s_at	AKR1B10	6	0.62104369	0.0190581	1.19355634	0.99994064	0.69582673	0.40779008	0.86569656	0.92618825
206569_at	IL24	6	0.3061902	0.00075504	1.30122159	0.99994064	0.65336753	0.68379087	0.6270562	0.63945472
206584_at	LY96	6	0.40584479	0.00305584	0.84027492	0.99994064	0.55947025	0.36601734	0.74523465	0.84626621
207777_s_at	SP140	6	0.49873084	0.00530551	0.98270707	0.99994064	0.71363915	0.61936934	0.74752825	0.78022398
208561_at	ABCC9	6	0.50543588	0.05722235	1.17307886	0.99994064	0.55223976	0.41827506	1.02082342	0.99910253
209875_s_at	SPP1	6	0.38800128	0.01055883	0.90283442	0.99994064	0.44995938	0.21905561	0.58833102	0.56953648
209949_at	NCF2	6	0.62602839	0.00993755	1.22711177	0.99994064	0.9210172	0.93452638	1.05716458	0.99101785
210118_s_at	IL1A	6	0.63447417	0.28732558	1.58165323	0.99994064	0.76436485	0.81585862	1.00097088	0.99950756
212325_at	LIMCH1	6	0.56969698	0.00110254	1.16416859	0.99994064	0.8164671	0.69779279	0.8918667	0.94204535
212328_at	LIMCH1	6	0.56162017	0.00550392	1.15215382	0.99994064	0.94219658	0.96894978	0.85882336	0.9252345
214257_s_at	SEC22B	6	0.72428697	0.0438434	1.00124844	0.99994064	0.7788586	0.49303177	1.01639886	0.99854069
216250_s_at	LPXN	6	0.46752	0.00128489	0.99015983	0.99994064	0.72561021	0.61548491	0.63797273	0.22701285
218503_at	KIAA1797	6	0.63723614	0.01552894	1.05396966	0.99994064	0.721381	0.41827506	0.86208379	0.90301016
219975_x_at	OLAH	6	0.26713977	0.02197142	1.00718823	0.99994064	0.45740161	0.60445637	0.98518507	0.99910253
220468_at	ARL14	6	0.43512445	0.00857209	0.9063457	0.99994064	0.63019345	0.5836006	0.73241505	0.8343613
220658_s_at	ARNTL2	6	0.69576242	0.01415673	0.9454458	0.99994064	0.77544695	0.42282677	0.80318264	0.53308188
222632_s_at	LZTFL1	6	0.72079788	0.03739485	0.91273418	0.99994064	0.85111934	0.73877098	0.94839941	0.98830037
222945_x_at	OLAH	6	0.42171298	0.01096117	1.12464265	0.99994064	0.61013483	0.5837945	0.86270148	0.98050935

Table S1. List of all probesets (514), mapping to 430 unique genes, that were significant in two or more comparisons across conditions, or were differentially expressed by any fragment of SV40 LT.

224204_x_at	ARNTL2	6	0.73492385	0.03532758	0.90504828	0.99994064	0.76300619	0.34806814	0.78570882	0.34580299
225314_at	OCIAD2	6	0.68848637	0.00898199	0.97761162	0.99994064	0.81742976	0.6019607	0.86096908	0.80999281
227699_at	C14orf149	6	0.65487888	0.00561982	0.96475421	0.99994064	0.71489338	0.21905561	0.90670177	0.94832097
231736_x_at	MGST1	6	0.69709797	0.03484985	0.99817638	0.99994064	0.79062565	0.59027794	0.90460926	0.95403463
233126_s_at	OLAH	6	0.37795857	0.04338707	1.06017322	0.99994064	0.48018647	0.50096101	0.93506161	0.99848066
240259_at	FLRT2	6	0.48883969	0.01019474	0.96070584	0.99994064	0.77247849	0.78429765	0.81071496	0.91851644
1561757_a_at	LOC283352	7	0.82362908	0.30844889	0.90398245	0.99994064	1.16626853	0.73832423	0.79874118	0.45811548
201286_at	SDC1	7	0.75225829	0.31481355	0.93900191	0.99994064	1.19176518	0.8156947	0.74649267	0.60103011
201301_s_at	ANXA4	7	0.61130601	0.05722235	0.83649762	0.99994064	1.17318728	0.86915034	0.60091521	0.1255934
201944_at	HEXB	7	0.55906968	0.02261801	0.46647338	0.04641559	1.15423208	0.89616308	0.48156418	0.007218
202074_s_at	OPTN	7	0.80305275	0.3659962	0.91870059	0.99994064	1.16869624	0.7873619	0.78838196	0.60545831
202307_s_at	TAP1	7	0.78743532	0.2780422	0.9228491	0.99994064	1.1859693	0.75213142	0.76735532	0.46510865
203827_at	WIPI1	7	0.68295701	0.16848754	0.88258064	0.99994064	1.39887463	0.58501512	0.71735874	0.57219548
203833_s_at	TGOLN2	7	0.89842294	0.76682479	1.01374865	0.99994064	1.48823257	0.17604122	0.97139727	0.99625173
204044_at	QPRT	7	0.82467638	0.46346173	0.89473561	0.99994064	1.19573691	0.7424078	0.82235506	0.7842592
205077_s_at	PIGF	7	0.39135054	1.65E-05	0.42990804	0.0012252	1.40886496	0.41827506	0.43491338	8.43E-05
205204_at	NMB	7	0.82463828	0.34226265	0.82501943	0.99994064	1.20608646	0.6524608	0.81443229	0.59729651
208191_x_at	PSG4	7	0.73972794	0.12059639	0.84627591	0.99994064	1.25049562	0.6251603	0.89568706	0.94427685
208796_s_at	CCNG1	7	0.72574435	0.20334512	0.91003882	0.99994064	1.26912426	0.69779279	0.71621601	0.41294141
209917_s_at	TP53TG1	7	0.72029843	0.33652541	0.87652471	0.99994064	1.23242513	0.81214927	0.73167084	0.69882411
210241_s_at	TP53TG1	7	0.72151435	0.21657762	0.87682854	0.99994064	1.23325118	0.76059627	0.6957785	0.34866536
210609_s_at	TP53I3	7	0.7287184	0.05718839	0.83965389	0.99994064	1.14596793	0.80405573	0.64049831	0.00885894
211272_s_at	DGKA	7	0.77797732	0.37594999	1.02394153	0.99994064	1.21156068	0.7727152	0.65938821	0.12945033
214366_s_at	ALOX5	7	0.741165	0.10960605	0.78943917	0.99994064	1.15468553	0.8056464	0.70014873	0.10229641
216705_s_at	ADA	7	0.89320713	0.71310473	0.93208494	0.99994064	1.23299476	0.61548491	0.89587333	0.92818473
221864_at	ORAI3	7	0.81262782	0.37171391	0.91825495	0.99994064	1.16309317	0.78429765	0.77281768	0.46496475
222020_s_at	NTM	7	0.71330945	0.2266294	0.73155251	0.99994064	1.2466588	0.7565444	0.72712065	0.56719061
223482_at	TMEM120A	7	0.84890009	0.58687048	0.94210951	0.99994064	1.36295066	0.41827506	0.79675054	0.6735439
225327_at	KIAA1370	7	0.77673803	0.50509445	0.83014389	0.99994064	1.31646272	0.70522809	0.81223363	0.87416107
225912_at	TP53INP1	7	0.46573031	0.00530551	0.63191394	0.99994064	1.58249397	0.48229992	0.28127112	2.98E-05
227020_at	YPEL2	7	0.74296526	0.55423483	0.79276582	0.99994064	1.5240744	0.6019607	0.88960286	0.98283075
227204_at	PARD6G	7	0.77451585	0.43532996	1.0687566	0.99994064	1.45582136	0.43246256	0.65182955	0.1948568
227566_at	NTM	7	0.72550963	0.19552155	0.77887659	0.99994064	1.14525326	0.86885497	0.72408619	0.44848517
227711_at	GTSF1	7	0.52849681	0.0125481	0.37145688	0.00194853	1.51701288	0.49520273	0.41949727	0.00140489
228989_at	C18orf56	7	0.75936566	0.44536318	0.89885896	0.99994064	1.27706622	0.75120643	0.78158066	0.81211107
232132_at	PARD6G	7	0.92858121	0.8903261	0.9952287	0.99994064	1.42046833	0.40779008	0.90385714	0.96797439
238432_at	FLJ35776	7	0.72873524	0.0565369	0.88358041	0.99994064	1.07378116	0.93262906	0.75572504	0.27964686
1552690_a_at	CACNA2D4	8	0.63286367	0.00877713	0.9864606	0.99994064	0.94485619	0.96353808	0.71866932	0.2348615
1558930_at	LOC728192	8	0.65471246	0.00560043	1.0067927	0.99994064	0.99290895	0.99789046	0.99277131	0.99910253
200730_s_at	PTP4A1	8	0.62627434	0.00288844	1.09875267	0.99994064	0.93460803	0.94179379	0.68194786	0.04162502
202363_at	SPOCK1	8	0.60645255	0.00223288	1.04760874	0.99994064	0.94737007	0.96281548	0.82884065	0.7842592
202555_s_at	MYLK	8	0.60523472	0.00276306	0.94487802	0.99994064	0.88484705	0.85016325	0.78501928	0.56701162
202627_s_at	SERPINE1	8	0.61027582	0.01167286	0.99405667	0.99994064	0.79329718	0.69779279	0.65580251	0.10229641
202628_s_at	SERPINE1	8	0.60744822	0.03739485	1.12147697	0.99994064	0.82195615	0.80940813	0.67553387	0.34804618
202672_s_at	ATF3	8	0.68673878	0.03538239	1.25922259	0.99994064	0.98746396	0.99585508	0.92472737	0.97742695
202693_s_at	STK17A	8	0.65356381	0.00200762	1.11039014	0.99994064	0.90369009	0.84681968	0.80647404	0.4732872
202862_at	FAH	8	0.72811253	0.05606233	1.04753613	0.99994064	1.10432642	0.87298028	0.84527929	0.80396464
203060_s_at	PAPSS2	8	0.62309901	0.03764964	1.01323349	0.99994064	1.00802634	0.99808936	0.89875513	0.97265147
204140_at	TPST1	8	0.61273422	0.03719865	0.94278454	0.99994064	0.80735029	0.77699108	0.79938736	0.82491264
204337_at	RGS4	8	0.50322192	3.85E-05	1.00621133	0.99994064	0.84114903	0.72335067	0.78024548	0.42911489
204338_s_at	RGS4	8	0.43639304	3.54E-06	0.93452165	0.99994064	0.77706113	0.49714557	0.71775663	0.10558273
204339_s_at	RGS4	8	0.58235334	0.00110254	1.10939003	0.99994064	0.98602764	0.99459195	0.82450492	0.77730276
204388_s_at	MAOA	8	0.54337999	0.00361447	1.36310812	0.99994064	0.89430158	0.91195336	0.9968857	0.99910253
204389_at	MAOA	8	0.58644457	0.01096117	1.25090018	0.99994064	0.91702523	0.94565929	0.95026382	0.99287355
204855_at	SERPINB5	8	0.55007491	0.00537026	1.02676071	0.99994064	0.87157698	0.874341	0.67164308	0.25673942
204955_at	SRPX	8	0.56495192	0.00246771	1.02242853	0.99994064	0.82142457	0.74299078	0.75743815	0.54436327
205428_s_at	CALB2	8	0.62102934	0.00200762	1.04311629	0.99994064	0.80876922	0.61548491	0.75777074	0.28749191
205499_at	SRPX2	8	0.64254378	0.01297805	0.85529855	0.99994064	0.85860511	0.80940813	0.75432946	0.45481923
205850_s_at	GABRB3	8	0.62707069	0.00724465	0.87535088	0.99994064	0.95142818	0.96884526	0.79772681	0.67836126
206029_at	ANKRD1	8	0.47188264	0.03755953	1.04906204	0.99994064	0.75347593	0.81664618	0.65383567	0.72738792
206060_s_at	PTPN22	8	0.65911404	0.0424872	1.12469462	0.99994064	0.91024911	0.92440563	0.77947068	0.68883745
206099_at	PRKCH	8	0.71653049	0.02272444	0.87630197	0.99994064	0.94618881	0.95166116	0.87347198	0.85290017
206343_s_at	NRG1	8	0.56530447	0.00904918	1.0339024	0.99994064	0.83381544	0.81585862	0.74827129	0.65386431

Table S1. List of all probesets (514), mapping to 430 unique genes, that were significant in two or more comparisons across conditions, or were differentially expressed by any fragment of SV40 LT.

206382_s_at	BDNF	8	0.69372382	0.07251979	1.37481103	0.99994064	1.17592822	0.80641561	0.94927632	0.99129953
207148_x_at	MYOZ2	8	0.60653662	0.02407543	1.11028753	0.99994064	0.98541272	0.99602346	0.94807077	0.99233905
208012_x_at	SP110	8	0.56420838	0.00080143	0.90488101	0.99994064	0.95762541	0.97206523	0.68215272	0.07342718
208392_x_at	SP110	8	0.78789029	0.14887791	0.98995396	0.99994064	1.12033744	0.82261432	0.72168108	0.05761139
209276_s_at	GLRX	8	0.60585033	0.02467619	0.93012724	0.99994064	0.90869413	0.93897519	0.72331702	0.53473829
209295_at	TNFRSF10B	8	0.68191634	0.06247481	1.12982553	0.99994064	1.0161171	0.99459195	0.81351076	0.80396464
209386_at	TM4SF1	8	0.626448	0.04243286	1.11247018	0.99994064	0.88759083	0.90502788	0.88691433	0.96494586
209387_s_at	TM4SF1	8	0.63897586	0.00574535	0.98965666	0.99994064	0.82941536	0.70522809	0.80641814	0.66069464
209761_s_at	SP110	8	0.43928628	3.34E-05	0.92148548	0.99994064	0.9643085	0.98044795	0.57266632	0.00404559
209762_x_at	SP110	8	0.55326146	0.00032961	0.84950833	0.99994064	0.81300342	0.64276017	0.6256958	0.00681617
209906_at	C3AR1	8	0.63531031	0.00969662	0.96870771	0.99994064	0.90277185	0.89267817	0.76099911	0.47360341
210405_x_at	TNFRSF10B	8	0.73611343	0.04338707	1.14210873	0.99994064	1.03905114	0.97128455	0.83572488	0.71431949
210510_s_at	NRP1	8	0.67715906	0.02198092	0.88084902	0.99994064	0.91482435	0.90958416	0.86154616	0.86981297
210511_s_at	INHBA	8	0.64486392	0.05139732	1.16929045	0.99994064	1.04080515	0.98236846	1.01480322	0.99890077
210788_s_at	DHRS7	8	0.70828407	0.0164833	0.98746396	0.99994064	0.91512031	0.88061722	0.77995709	0.32442485
212298_at	NRP1	8	0.5592247	0.00590408	0.76968142	0.99994064	0.68987171	0.41827506	0.7282303	0.53308188
212741_at	MAOA	8	0.68101886	0.01724755	1.19060924	0.99994064	0.97326191	0.98494853	0.92594602	0.97448747
213782_s_at	MYOZ2	8	0.61829475	0.02261801	1.30107128	0.99994064	1.08962623	0.94565929	0.96148305	0.99434695
215034_s_at	TM4SF1	8	0.63484076	0.03532758	1.13039997	0.99994064	0.78006523	0.68411508	0.83402739	0.87045273
216064_s_at	AGA	8	0.61339997	0.00033305	0.87510821	0.99994064	0.87185895	0.74829401	0.75076502	0.10229641
218403_at	TRIAP1	8	0.63822337	0.00122781	1.05760434	0.99994064	0.9379611	0.93184711	0.73070788	0.07189075
218764_at	PRKCH	8	0.61030402	0.00472673	0.83208338	0.99994064	0.78718065	0.61548491	0.82922374	0.80837584
219936_s_at	GPR87	8	0.42057451	0.00158733	0.95579072	0.99994064	0.86035263	0.90502788	0.50264091	0.0312314
220217_x_at	SPANXC	8	0.44304751	1.96E-05	1.23305174	0.99994064	0.98441144	0.99406226	0.86080995	0.8742763
220979_s_at	ST6GALNAC5	8	0.61303159	0.00797215	0.94710744	0.99994064	0.94960537	0.96998575	0.80946092	0.7842592
221504_s_at	ATP6V1H	8	0.7319752	0.04063152	1.01915018	0.99994064	0.90139624	0.85374854	0.85239852	0.79832722
222530_s_at	MKKS	8	0.70738458	0.04142548	0.92076187	0.99994064	0.94439785	0.9579479	0.83818077	0.80267533
222838_at	SLAMF7	8	0.55049448	0.0005481	1.03077777	0.99994064	0.87038967	0.81731633	0.86691753	0.88990704
222862_s_at	AK5	8	0.68886825	0.02441643	0.91899781	0.99994064	0.92425745	0.92367195	0.85267429	0.83393233
224823_at	MYLK	8	0.4546516	0.00392744	0.9300198	0.99994064	0.93889344	0.97469929	0.66086767	0.52070598
225538_at	ZCCHC9	8	0.71080874	0.03408886	0.91929513	0.99994064	0.88775491	0.83690925	0.84231592	0.79116578
225944_at	NLN	8	0.72178113	0.02250025	1.07882934	0.99994064	1.01073164	0.99567542	0.99674751	0.99910253
225967_s_at	C17orf89	8	0.69885578	0.02429104	1.03201695	0.99994064	0.93396043	0.93897519	0.80032987	0.57219548
226218_at	IL7R	8	0.65082128	0.03178412	1.21846657	0.99994064	0.90371097	0.91299775	0.90525742	0.96881546
226248_s_at	KIAA1324	8	0.65459145	0.0376611	0.98150442	0.99994064	0.82100714	0.75213142	0.75071298	0.54185783
226606_s_at	GTPBP5	8	0.73187373	0.08722087	1.03270868	0.99994064	1.03363966	0.98003235	0.89655666	0.94204535
226767_s_at	FAHD1	8	0.65076114	0.04602249	0.94104351	0.99994064	0.883805	0.88549993	0.89001404	0.96074256
228080_at	LAYN	8	0.68452098	0.04783399	0.91615694	0.99994064	0.96326189	0.97868467	0.88097114	0.93094177
228083_at	CACNA2D4	8	0.73304144	0.04465174	1.02505406	0.99994064	0.94942986	0.95808782	0.78501928	0.39280392
229406_at	RBFOX3	8	0.54042513	0.03719865	1.02337389	0.99994064	0.88323342	0.93834013	0.59844891	0.26887106
231504_at	CCDC148	8	0.26758454	1.38E-08	0.97740835	0.99994064	0.74582032	0.40779008	0.59438379	0.00254631
234306_s_at	SLAMF7	8	0.57134471	0.00074196	0.93869822	0.99994064	0.78293621	0.55155712	0.76916588	0.4393986
236249_at	IKBIP	8	0.6084877	0.02096333	1.10098896	0.99994064	1.0081428	0.99808936	0.79121042	0.79510349
241994_at	XDH	8	0.56338771	0.00128553	1.10782756	0.99994064	0.97189116	0.98532298	0.90106307	0.95852091
243357_at	NEGR1	8	0.69122789	0.04051229	0.95420203	0.99994064	0.87403725	0.83148903	0.88064553	0.92189574
1554628_at	ZNF57	9	0.79452618	0.19833184	1.01175969	0.99994064	0.89434291	0.83973657	1.22168019	0.6228274
1554797_at	SYT16	9	0.68510642	0.02578472	0.7511988	0.99994064	0.8376387	0.73883918	0.85965717	0.86648081
1555007_s_at	WDR66	9	0.72273233	0.04338707	0.96023982	0.99994064	0.93445688	0.93805307	0.93525607	0.98017072
1557137_at	TMEM17	9	0.64864454	0.01096117	0.85462692	0.99994064	0.89176367	0.86783119	0.9180216	0.97142586
1559060_a_at	FNIP1	9	1.0295163	0.96060182	0.82490506	0.99994064	0.91096445	0.91507628	1.37496987	0.30375459
200799_at	HSPA1A	9	1.05044454	0.94873007	1.25518499	0.99994064	0.9242788	0.96246514	2.26639606	0.00131237
202708_s_at	HIST2H2BE	9	0.69827473	0.0494851	1.00254477	0.99994064	1.03399796	0.98158407	1.14467126	0.90561205
203548_s_at	LPL	9	1.18531184	0.37171391	1.25359095	0.99994064	1.1289644	0.78877484	1.53354092	0.00260936
203973_s_at	CEBPD	9	0.81920703	0.39948722	1.28960866	0.99994064	1.16977684	0.7727152	1.20960276	0.77781309
205553_s_at	CSRP3	9	0.79121042	0.64410081	0.91806402	0.99994064	2.00282079	0.1196617	1.64136944	0.27631388
205625_s_at	CALB1	9	0.825191	0.5910332	1.19082934	0.99994064	1.00832916	0.99808936	2.47643828	5.12E-05
206025_s_at	TNFAIP6	9	0.66494181	0.04175417	0.82953035	0.99994064	0.94422331	0.96523524	0.94588279	0.99118251
206300_s_at	PTHLH	9	0.66120368	0.03495718	0.91950755	0.99994064	0.80613871	0.70522809	1.02479357	0.99848066
207156_at	HIST1H2AG	9	0.62659276	0.08351028	0.9147398	0.99994064	1.07606607	0.96523524	1.14970734	0.96109593
209146_at	SC4MOL	9	0.62772301	0.0125481	1.0060486	0.99994064	0.98557211	0.99567542	1.18813601	0.85813253
209218_at	SQLE	9	0.76629227	0.157134	1.08239969	0.99994064	0.94258851	0.95601658	1.17649892	0.81568517
209398_at	HIST1H1C	9	0.46942503	0.00074196	0.92257195	0.99994064	1.18134744	0.84347422	1.09436955	0.9817172
214321_at	NOV	9	0.64015803	0.07188551	1.05926728	0.99994064	1.20360889	0.81870906	1.38255161	0.57346777

Table S1. List of all probesets (514), mapping to 430 unique genes, that were significant in two or more comparisons across conditions, or were differentially expressed by any fragment of SV40 LT.

219279_at	DOCK10	9	0.6507461	0.02085578	0.93744113	0.99994064	0.92530443	0.94292803	1.09862574	0.96921031
221750_at	HMGCS1	9	0.67609599	0.08956111	1.27241269	0.99994064	0.95499605	0.97570425	1.37952028	0.47852898
226066_at	MITF	9	0.60879708	0.16480418	1.15666146	0.99994064	1.22490229	0.86658866	1.0233266	0.99872331
227896_at	BCCIP	9	0.82410496	0.44095857	1.19933387	0.99994064	0.94686676	0.96349406	1.24151354	0.68068949
229657_at	THRB	9	0.70578467	0.04684815	0.8002744	0.99994064	0.99683963	0.99895464	0.97919403	0.99848066
235035_at	SLC35E1	9	0.71082517	0.14093522	1.07427747	0.99994064	0.78883748	0.68526247	1.198669	0.86648081
239835_at	KBTBD8	9	0.65394143	0.14527849	1.29851859	0.99994064	0.89357868	0.93513884	1.22889927	0.89576661
1570542_a_at	PMPCB	10	1.32816403	0.04602249	0.99261076	0.99994064	1.09781376	0.86059735	1.05165877	0.9847175
208729_x_at	HLA-B	10	1.41143889	0.01332292	1.05801983	0.99994064	1.24102599	0.5250086	0.88513331	0.87048307
209008_x_at	KRT8	10	1.50066297	0.06547784	0.95090075	0.99994064	1.29121866	0.66853748	0.90214631	0.97113514
209140_x_at	HLA-B	10	1.32690646	0.04766263	1.04809295	0.99994064	1.24925385	0.49303177	0.87193954	0.81706236
211799_x_at	HLA-C	10	1.13521587	0.56147234	0.92832379	0.99994064	1.09436955	0.86059735	0.81578827	0.46476315
211911_x_at	HLA-B	10	1.36081096	0.03197633	1.03189773	0.99994064	1.24220218	0.51188104	0.85697994	0.78940222
213932_x_at	HLA-A	10	1.37487456	0.02100802	1.07808181	0.99994064	1.14745163	0.75120643	1.02590703	0.99434695
215313_x_at	HLA-A	10	1.45387174	0.01453627	1.10356122	0.99994064	1.22677159	0.62203517	1.0132569	0.99872331
226086_at	SYT13	10	1.32629344	0.04202641	1.25898986	0.99994064	1.14753116	0.7424078	1.20792705	0.56701162
227440_at	ANKS1B	10	1.45693179	0.04777131	1.24447162	0.99994064	1.18260368	0.78291207	1.2040261	0.80999281
231366_at	FDPSL2A	10	1.41307039	0.03538239	1.22334671	0.99994064	1.24527698	0.60308797	1.17701551	0.80999281
200790_at	ODC1	11	0.96475421	0.94853217	1.14158108	0.99994064	0.75906745	0.50096101	1.112573	0.95090949
201377_at	UBAP2L	11	1.08665954	0.79672305	1.11961289	0.99994064	0.79953513	0.5250086	1.08465282	0.96238473
201498_at	USP7	11	1.03595883	0.94994433	1.13526833	0.99994064	0.77616395	0.59397682	1.29399616	0.5530008
201925_s_at	CD55	11	0.99718517	0.99812763	1.32580322	0.99994064	0.75109467	0.68379087	1.37173329	0.63418928
202147_s_at	IFRD1	11	0.90793862	0.81156472	1.0751714	0.99994064	0.69036601	0.2714868	1.15058428	0.90351332
202153_s_at	NUP62	11	1.06081028	0.89333498	1.22875731	0.99994064	0.75847139	0.41827506	1.24125541	0.62771027
203349_s_at	ETV5	11	0.86331961	0.70640605	1.06656114	0.99994064	0.66475748	0.27971563	0.99384998	0.99910253
205135_s_at	NUFIP1	11	0.91100655	0.83685428	1.19022418	0.99994064	0.77360374	0.61747846	1.20792705	0.82491264
205239_at	AREG	11	0.83319919	0.80466061	1.45676349	0.99994064	0.65738024	0.65080625	1.28835782	0.90957217
206157_at	PTX3	11	0.70405823	0.42048061	1.2526355	0.99994064	0.52060702	0.20249817	1.32289632	0.84911189
206729_at	TNFRSF8	11	0.9510106	0.91402318	1.18145663	0.99994064	0.76388816	0.47406958	1.09505247	0.96238473
208777_s_at	PSMD11	11	0.99187714	0.9878554	1.08879574	0.99994064	0.79011433	0.50096101	1.06242917	0.9817172
209332_s_at	MAX	11	0.91279745	0.77141122	1.13055669	0.99994064	0.78665338	0.49303177	1.11301009	0.92401476
209336_at	PWP2	11	1.02954008	0.95726128	1.20199703	0.99994064	0.7835515	0.56985118	1.14234625	0.90260264
212378_at	GART	11	1.02273568	0.9618116	1.19148985	0.99994064	0.86405796	0.75120643	1.16726598	0.80396464
212434_at	GRPEL1	11	1.08410162	0.84305517	1.1859145	0.99994064	0.82786456	0.72335067	1.1863804	0.81878618
212846_at	RRP1B	11	1.01087177	0.98669927	1.11829437	0.99994064	0.65800327	0.16136551	1.12199533	0.94204535
213302_at	PFAS	11	0.98034854	0.9783118	1.10106528	0.99994064	0.70681276	0.46916089	1.17527633	0.9042471
217834_s_at	SYNCRIP	11	0.85886304	0.52633526	1.06826285	0.99994064	0.77044649	0.41827506	1.05845994	0.98330741
218398_at	MRPS30	11	0.88861681	0.6835247	1.13264833	0.99994064	0.81103346	0.6019607	1.14356101	0.86722484
218593_at	RBM28	11	0.95103258	0.90648022	1.14702752	0.99994064	0.82721447	0.65965716	1.17102077	0.80915772
219177_at	BRIX1	11	1.03447587	0.93593418	1.14901689	0.99994064	0.85709875	0.71028425	1.22609152	0.52070598
219437_s_at	ANKRD11	11	0.98059773	0.97992396	1.13487495	0.99994064	0.6786	0.41827506	1.08052566	0.98814177
223124_s_at	PITHD1	11	0.91273418	0.82056797	1.17248272	0.99994064	0.73314307	0.41827506	1.11956115	0.94427685
223296_at	SLC25A33	11	0.94575168	0.89736623	1.18031069	0.99994064	0.8351651	0.69846214	1.16223354	0.83836884
223308_s_at	WDR5	11	1.00614158	0.99261119	1.1320989	0.99994064	0.78385932	0.5250086	1.09669828	0.95852091
225099_at	FBXO45	11	1.01150258	0.98244071	1.16215299	0.99994064	0.83926598	0.70522809	1.22436468	0.65386431
225100_at	FBXO45	11	0.92304102	0.85013947	1.17560223	0.99994064	0.80212557	0.64750459	1.18418953	0.82845816
225458_at	LOC25845	11	1.13183736	0.76991711	1.13718476	0.99994064	0.73361752	0.51148721	1.05692036	0.99121149
227093_at	USP36	11	0.93348581	0.89333498	1.22679994	0.99994064	0.72710385	0.42282677	1.16764361	0.88990704
227538_at	MED26	11	1.04848048	0.90898484	1.17973814	0.99994064	0.86635687	0.75397004	1.20519506	0.67775876
227856_at	C4orf32	11	0.95004428	0.93078366	1.13400998	0.99994064	0.68769146	0.34806814	1.13807844	0.94138329
232027_at	SYNE1	11	0.93885006	0.9363549	1.27470788	0.99994064	0.71023417	0.61747846	1.32470091	0.79253254
234985_at	LDLRAD3	11	0.99476892	0.99341835	1.23959313	0.99994064	0.84588494	0.72335067	1.17118312	0.8087995
242963_at	SGMS2	11	0.99316133	0.9904217	1.21762228	0.99994064	0.78932973	0.50096101	1.12238425	0.90674599
200965_s_at	ABLIM1	12	0.90008511	0.82735081	0.87964908	0.99994064	1.49267492	0.34401956	0.95420203	0.99361001
201447_at	TIA1	12	1.19855823	0.49030575	0.98209421	0.99994064	1.47706502	0.17604122	1.06005075	0.98882635
201506_at	TGFBI	12	1.49233007	0.17416055	0.96920024	0.99994064	1.66743825	0.27971563	0.86077018	0.95546666
202262_x_at	DDAH2	12	1.18627076	0.4825497	0.86292077	0.99994064	1.46581192	0.14586848	0.98463892	0.99854911
203136_at	RABAC1	12	0.89935753	0.78877212	0.79916575	0.99994064	1.27470788	0.61548491	0.7668059	0.53308188
203828_s_at	IL32	12	1.08214963	0.85484057	0.99559669	0.99994064	1.5153664	0.16136551	1.02391787	0.99848066
203989_x_at	F2R	12	0.94813649	0.92061967	0.85630699	0.99994064	1.32868581	0.5250086	0.91069087	0.96987498
204174_at	ALOX5AP	12	0.81994554	0.17898664	0.72742311	0.19782777	1.12355182	0.77882186	0.8920934	0.85533473
205157_s_at	KRT17	12	1.38092344	0.4862201	0.82157641	0.99994064	1.9022849	0.21905561	0.87857255	0.98017072
205347_s_at	TMSB15A	12	1.16607992	0.64594236	0.76001511	0.99994064	1.24392543	0.68379087	1.24711975	0.73553278

Table S1. List of all probesets (514), mapping to 430 unique genes, that were significant in two or more comparisons across conditions, or were differentially expressed by any fragment of SV40 LT.

206116_s_at	TPM1	12	0.86994735	0.8159694	0.75507926	0.99994064	1.50796192	0.51126476	0.80658585	0.89518287
207379_at	EDIL3	12	0.87377476	0.88619247	0.69483067	0.99994064	1.7586479	0.49303177	0.77121232	0.91577945
209459_s_at	ABAT	12	0.89560428	0.90338828	0.92312633	0.99994064	1.74831685	0.41827506	0.88280498	0.98305897
209460_at	ABAT	12	0.83314143	0.65118896	0.74747643	0.99994064	1.52168176	0.34401956	0.68138086	0.29918092
209904_at	TNNC1	12	1.60562316	0.25396203	0.53232037	0.99994064	2.66001307	0.0670455	0.76652248	0.89798574
210987_x_at	TPM1	12	0.86822046	0.80048981	0.74668242	0.99994064	1.44326211	0.57377671	0.82461922	0.91176273
212236_x_at	KRT17	12	1.14652409	0.79901149	0.84903739	0.99994064	1.48411203	0.49303177	0.85094237	0.94014069
218002_s_at	CXCL14	12	1.32865512	0.64392134	0.46577335	0.51936658	2.08753444	0.20148592	0.90668082	0.99110384
219014_at	PLAC8	12	1.03564771	0.96654746	0.99412557	0.99994064	1.97037014	0.110717	1.38335043	0.68883745
220382_s_at	ARHGAP28	12	0.91524718	0.86115441	0.71291402	0.99994064	1.29624043	0.63840491	0.81150206	0.80999281
221011_s_at	LBH	12	1.09171782	0.87811491	1.00556058	0.99994064	1.62702152	0.19645273	1.04164716	0.99434695
222016_s_at	ZNF323	12	0.83719368	0.70082454	0.75132031	0.99994064	1.30074065	0.73138299	0.80556152	0.86648081
222484_s_at	CXCL14	12	1.24257534	0.68895805	0.52725277	0.51936658	1.84719136	0.2102969	0.95651975	0.99725814
222846_at	RAB8B	12	0.92459919	0.90958851	0.76811807	0.99994064	1.47498471	0.50096101	0.88546059	0.96987498
222996_s_at	CXXC5	12	1.10004815	0.7845283	0.90798057	0.99994064	1.36755608	0.32424872	0.87973038	0.90086164
225275_at	EDIL3	12	0.86162579	0.84953447	0.63739812	0.99994064	1.60829642	0.55155712	0.84717583	0.96797439
225604_s_at	GLIPR2	12	0.98965666	0.98962515	0.82627848	0.99994064	1.36443153	0.54835265	0.90682747	0.97492246
226460_at	FNIP2	12	0.86679736	0.56566433	0.86667721	0.99994064	1.23219735	0.59666768	1.02242853	0.99848066
226884_at	LRRN1	12	0.71330945	0.69974032	0.70604563	0.99994064	1.80637822	0.6251603	0.67100713	0.86981297
227046_at	SLC39A11	12	0.9702533	0.95291674	0.851198	0.99994064	1.26590283	0.56985118	0.84068273	0.80396464
227145_at	LOXL4	12	0.85194567	0.68726541	0.78752629	0.99994064	1.40740089	0.48118484	0.78047987	0.74789057
235048_at	FAM169A	12	1.01227411	0.98971578	0.70928303	0.99994064	1.2084016	0.83585882	0.70039143	0.5530008
236529_at	SRCRB4D	12	1.06900357	0.90965111	0.88941789	0.99994064	1.52178724	0.28291447	0.81313493	0.81956392
243521_at	ZXDA	12	1.13728986	0.79714434	0.71747477	0.99994064	1.38741559	0.57543814	1.15976567	0.94210633
49306_at	RASSF4	12	0.95219789	0.93661106	0.85565433	0.99994064	1.42836696	0.41756041	0.87026901	0.93094177
1554195_a_at	C5orf46	13	0.5138313	0.00064622	0.86495681	0.99994064	1.48126867	0.29055558	0.80584075	0.78584443
1555579_s_at	PTPRM	13	0.61139077	0.0494851	1.05789761	0.99994064	1.06098187	0.97206523	0.92159194	0.98882635
1564837_at	LOC151760	13	0.58131821	0.00315711	0.98320671	0.99994064	0.95378324	0.97206523	0.69491094	0.1907335
1564838_a_at	LOC151760	13	0.51757267	0.00134983	0.95813444	0.99994064	0.93462962	0.96328349	0.69780701	0.32442485
201069_at	MMP2	13	0.62817278	0.00467744	0.86729819	0.99994064	1.03218388	0.98167965	0.7740686	0.47852898
201798_s_at	MYOF	13	0.53212361	0.01484806	0.90316824	0.99994064	1.00365725	0.99919584	0.67102263	0.47852898
203640_at	MBNL2	13	0.67509698	0.05718839	0.87820724	0.99994064	1.32819472	0.57377671	0.94058702	0.98917858
203695_s_at	DFNA5	13	0.65941868	0.04839571	0.85462692	0.99994064	1.0030545	0.99912908	0.71182771	0.34833564
204093_at	CCNH	13	0.70926664	0.01552894	0.87214102	0.99994064	0.99481489	0.99808936	0.8485079	0.77858017
205919_at	HBE1	13	0.72954388	0.11291044	1.07244228	0.99994064	1.1344817	0.85258586	0.9794203	0.99848066
208055_s_at	HERC4	13	0.65142305	0.01123718	0.82269714	0.99994064	0.90387802	0.8879806	0.72601269	0.2348615
208965_s_at	IFI16	13	0.6651262	0.0051616	0.89026084	0.99994064	0.98917659	0.99585508	0.94476887	0.98305897
209758_s_at	MFAP5	13	0.37808085	0.00109199	0.72812935	0.99994064	0.8906929	0.95009652	0.6650801	0.61692629
211864_s_at	MYOF	13	0.58733954	0.01718147	0.86057132	0.99994064	1.05521233	0.97262467	0.63168038	0.13014897
212240_s_at	PIK3R1	13	0.71312819	0.02410652	0.8397509	0.99994064	1.00495669	0.99809955	0.93980499	0.9817172
213139_at	SNAI2	13	0.4289258	0.00169241	0.78818161	0.99994064	1.08575606	0.96510141	0.72346744	0.7608512
213923_at	RAP2B	13	0.71989912	0.04338707	0.91497232	0.99994064	0.95226389	0.96353808	0.87290708	0.87045273
214074_s_at	CTTN	13	0.69624486	0.034571	0.88945899	0.99994064	0.93122391	0.9391773	0.83694226	0.80396464
214657_s_at	NEAT1	13	0.60557043	0.01341026	0.95175798	0.99994064	1.07221929	0.96058153	0.71847009	0.41008294
216218_s_at	PLCL2	13	0.71261759	0.04755625	1.07001671	0.99994064	1.21366198	0.68411508	0.88060483	0.9042471
217974_at	TM7SF3	13	0.64586296	0.05226904	1.07745927	0.99994064	1.03749185	0.98423422	0.77469482	0.75160899
217995_at	SQRDL	13	0.65433439	0.03866866	0.79068045	0.99994064	1.01581194	0.99516526	0.80086631	0.7842592
218109_s_at	MFSD1	13	0.62998964	0.02198592	0.85383744	0.99994064	0.97706966	0.99092295	0.79346216	0.77730276
218718_at	PDGFC	13	0.56907867	0.00331064	0.7560394	0.99994064	0.91208067	0.92781643	0.78300857	0.69563604
218943_s_at	DDX58	13	0.59923758	0.04338707	1.03063489	0.99994064	1.15992645	0.88250442	0.8165803	0.88918515
220014_at	PRR16	13	0.50331494	0.00732854	0.76879277	0.99994064	1.03340087	0.99039879	0.78687152	0.85533473
222127_s_at	EXOC1	13	0.70218999	0.03638322	0.93029918	0.99994064	0.95682921	0.97080669	0.81605219	0.69882411
222408_s_at	YPEL5	13	0.66209035	0.03539946	0.88231558	0.99994064	1.10869817	0.90502788	0.76632769	0.58271189
222477_s_at	TM7SF3	13	0.52956022	0.00048936	1.03473882	0.99994064	1.08040084	0.93983075	0.53267716	0.00097867
222719_s_at	PDGFC	13	0.59035991	0.00094262	0.77948869	0.99994064	0.99380405	0.99808936	0.68117623	0.04147211
223204_at	FAM198B	13	0.51441335	0.01667898	0.86258189	0.99994064	1.26836209	0.80732068	0.73227968	0.7842592
224558_s_at	MALAT1	13	0.64571376	0.03585723	0.97119529	0.99994064	1.19819828	0.7873619	0.80064429	0.78940222
224565_at	NEAT1	13	0.69129178	0.07965877	1.03821123	0.99994064	1.17419064	0.81233634	0.80985377	0.79832722
225496_s_at	SYTL2	13	0.62166101	0.04908365	0.81673125	0.99994064	0.93806947	0.9699485	0.6732278	0.331729
226478_at	TM7SF3	13	0.64948435	0.04051229	1.13115764	0.99994064	1.23641809	0.74133465	0.62481457	0.05339533
226997_at	ADAMTS12	13	0.67310338	0.03178412	0.93307611	0.99994064	1.13508474	0.85388769	0.94605765	0.98975404
230147_at	F2RL2	13	0.61906666	0.03538239	0.95755903	0.99994064	1.20480528	0.80940813	0.81803435	0.86101918
235230_at	PLCXD2	13	0.57502636	0.00993755	0.94426694	0.99994064	0.87981168	0.88813484	0.74270782	0.60705361

Table S1. List of all probesets (514), mapping to 430 unique genes, that were significant in two or more comparisons across conditions, or were differentially expressed by any fragment of SV40 LT.

236984_at	C4orf26	13	0.46248121	0.00021198	0.95191192	0.99994064	1.12378548	0.89279541	0.68976014	0.25673942
240228_at	CSMD3	13	0.54307876	0.03885254	1.17150789	0.99994064	1.15132888	0.91840501	0.64880942	0.49389084
208180_s_at	HIST1H4H	14	0.48064157	0.00530551	1.04560166	0.99994064	0.9671199	0.99044084	0.96372939	0.99848066
208490_x_at	HIST1H2BF	14	0.58201705	0.02107353	1.00094775	0.99994064	1.00073963	0.99973577	1.12708787	0.96881546
208523_x_at	HIST1H2BI	14	0.59029172	0.01851068	0.95508431	0.99994064	0.87458267	0.88526989	1.06964594	0.99101785
208527_x_at	HIST1H2BE	14	0.56474311	0.01218007	0.98901662	0.99994064	1.00714169	0.99809955	1.07075865	0.99101785
208546_x_at	HIST1H2BH	14	0.5749068	0.00802965	0.98607321	0.99994064	0.94302418	0.9699485	1.13981525	0.95090949
208579_x_at	H2BFS	14	0.58818151	0.0190581	1.05165877	0.99994064	1.00581618	0.99895464	1.10221067	0.98017072
209806_at	HIST1H2BK	14	0.65055067	0.02245441	1.03821123	0.99994064	0.98851402	0.99740584	1.1514885	0.91316827
209911_x_at	HIST1H2BD	14	0.52306664	0.00103798	0.96092784	0.99994064	0.88607456	0.88250442	1.08262479	0.9817172
214455_at	HIST1H2BC	14	0.37595538	0.00049555	1.0603202	0.99994064	0.8476849	0.88773889	1.43385586	0.66666677
215071_s_at	HIST1H2AC	14	0.44684096	0.00127199	0.91859447	0.99994064	0.89258822	0.93323547	0.95455485	0.99469897
222067_x_at	HIST1H2BD	14	0.58727169	0.00560043	0.93419783	0.99994064	1.03023015	0.98590897	1.15029189	0.9252345

Table S2. List of all enriched GO terms for each cluster (Figure 2B), along with their adjusted p-values and log odds ratios.

Cluster number	Log Odds Ratio	Adjusted p-value	GO term ID	GO term
1	2.67759596	0.05	GO:0060452	positive regulation of cardiac muscle contraction
2	2.7975533	0.041	GO:0000212	meiotic spindle organization
2	2.37775461	0.001	GO:0007076	mitotic chromosome condensation
2	2.11226427	0.016	GO:0051983	regulation of chromosome segregation
2	2.02277589	0.027	GO:0030261	chromosome condensation
2	1.93185848	0.03	GO:0006323	DNA packaging
2	1.93185848	0.03	GO:0006336	DNA replication-independent nucleosome assembly
2	1.93185848	0.03	GO:0034080	CenH3-containing nucleosome assembly at centromere
2	1.93185848	0.03	GO:0034724	DNA replication-independent nucleosome organization
2	1.90021549	0.049	GO:0031055	chromatin remodeling at centromere
2	1.82029702	0.001	GO:0006333	chromatin assembly or disassembly
2	1.65502406	0.016	GO:0007059	chromosome segregation
2	1.64900967	<0.001	GO:0022402	cell cycle process
2	1.64758784	0.001	GO:0000087	M phase of mitotic cell cycle
2	1.63750177	0.001	GO:0000279	M phase
2	1.59689732	<0.001	GO:0022403	cell cycle phase
2	1.5923339	<0.001	GO:0000278	mitotic cell cycle
2	1.58271141	0.029	GO:0000236	mitotic prometaphase
2	1.58119181	<0.001	GO:0007049	cell cycle
2	1.53781186	0.001	GO:0000082	G1/S transition of mitotic cell cycle
2	1.5148242	<0.001	GO:0000280	nuclear division
2	1.5148242	<0.001	GO:0007067	mitosis
2	1.498227	<0.001	GO:0051301	cell division
2	1.48485125	<0.001	GO:0048285	organelle fission
2	1.44426916	<0.001	GO:0005694	chromosome
2	1.38064585	<0.001	GO:0007017	microtubule-based process
2	1.37515722	0.017	GO:0000226	microtubule cytoskeleton organization
2	1.23377762	<0.001	GO:0044427	chromosomal part
2	1.21777335	<0.001	GO:0006259	DNA metabolic process
2	1.20807048	0.001	GO:0010564	regulation of cell cycle process
2	1.17083647	<0.001	GO:0051276	chromosome organization
2	1.14434331	0.045	GO:0006281	DNA repair
2	1.13042197	<0.001	GO:0006996	organelle organization
2	1.11452964	0.001	GO:0006974	response to DNA damage stimulus
2	1.07643951	0.001	GO:0051726	regulation of cell cycle
2	1.05114504	0.001	GO:0009987	cellular process
2	1.0393985	0.001	GO:0005654	nucleoplasm
2	1.02507153	<0.001	GO:0005634	nucleus
2	1.02295734	0.001	GO:0044424	intracellular part
2	0.93170518	<0.001	GO:0071842	cellular component organization at cellular level
2	0.92569804	<0.001	GO:0071841	cellular component organization or biogenesis at cellular level
2	0.92219565	0.001	GO:0043229	intracellular organelle
2	0.92085801	0.001	GO:0043226	organelle
2	0.90319606	0.001	GO:0044428	nuclear part
2	0.86025614	0.001	GO:0016043	cellular component organization
2	0.85538873	0.001	GO:0071840	cellular component organization or biogenesis
2	0.85206066	0.001	GO:0044446	intracellular organelle part
2	0.84332577	0.001	GO:0044422	organelle part
2	0.8067168	0.001	GO:0043231	intracellular membrane-bounded organelle
2	0.80634992	0.001	GO:0043227	membrane-bounded organelle
2	0.80054976	0.005	GO:0043228	non-membrane-bounded organelle
2	0.80054976	0.005	GO:0043232	intracellular non-membrane-bounded organelle
2	0.79775842	0.005	GO:0090304	nucleic acid metabolic process
2	0.68037088	0.03	GO:0044260	cellular macromolecule metabolic process
2	0.66639715	0.03	GO:0043170	macromolecule metabolic process
3	1.16371524	<0.001	GO:0006974	response to DNA damage stimulus
3	1.07739737	<0.001	GO:0033554	cellular response to stress
4	1.5552561	0.019	GO:0045834	positive regulation of lipid metabolic process
4	1.04123568	0.024	GO:0044464	cell part
5	2.90685157	0.019	GO:0051434	BH3 domain binding
5	2.90685157	0.019	GO:0097136	Bcl-2 family protein complex
8	2.79711359	0.043	GO:0016822	hydrolase activity, acting on acid carbon-carbon bonds
8	2.79711359	0.043	GO:0016823	hydrolase activity, acting on acid carbon-carbon bonds, in ketonic substances

Table S2. List of all enriched GO terms for each cluster (Figure 2B), along with their adjusted p-values and log odds ratios.

9	2.00300983	0.017	GO:0006695	cholesterol biosynthetic process
9	1.9248848	0.027	GO:0016126	sterol biosynthetic process
10	3.22494722	<0.001	GO:0071556	integral to lumenal side of endoplasmic reticulum membrane
10	2.96954298	<0.001	GO:0032393	MHC class I receptor activity
10	2.88232692	<0.001	GO:0042612	MHC class I protein complex
10	2.80971036	<0.001	GO:0012507	ER to Golgi transport vesicle membrane
10	2.66023652	<0.001	GO:0042611	MHC protein complex
10	2.46072822	0.002	GO:0030658	transport vesicle membrane
10	2.34688303	0.004	GO:0030176	integral to endoplasmic reticulum membrane
10	2.30994632	0.004	GO:0060333	interferon-gamma-mediated signaling pathway
10	2.3029194	0.004	GO:0060337	type I interferon-mediated signaling pathway
10	2.3029194	0.004	GO:0071357	cellular response to type I interferon
10	2.29600368	0.004	GO:0034340	response to type I interferon
10	2.23824122	0.006	GO:0071346	cellular response to interferon-gamma
10	2.23226987	0.008	GO:0031227	intrinsic to endoplasmic reticulum membrane
10	2.2035774	0.008	GO:0002474	antigen processing and presentation of peptide antigen via MHC class I
10	2.17145814	0.008	GO:0048002	antigen processing and presentation of peptide antigen
10	2.15624068	0.008	GO:0030662	coated vesicle membrane
10	2.15624068	0.008	GO:0034341	response to interferon-gamma
10	2.03128313	0.014	GO:0019882	antigen processing and presentation
10	1.94940264	0.019	GO:0031301	integral to organelle membrane
10	1.8857391	0.024	GO:0031300	intrinsic to organelle membrane
10	1.80922506	0.033	GO:0019221	cytokine-mediated signaling pathway
10	1.75165222	0.011	GO:0044419	interspecies interaction between organisms
10	1.72485364	0.048	GO:0071345	cellular response to cytokine stimulus
11	3.00582925	0.016	GO:0006188	IMP biosynthetic process
11	3.00582925	0.016	GO:0006189	'de novo' IMP biosynthetic process
11	2.78395852	0.025	GO:0046040	IMP metabolic process
11	0.77787393	0.006	GO:0044428	nuclear part
14	3.28037503	<0.001	GO:0000786	nucleosome
14	3.15913757	<0.001	GO:0032993	protein-DNA complex
14	3.11140589	<0.001	GO:0006334	nucleosome assembly
14	3.068363	<0.001	GO:0065004	protein-DNA complex assembly
14	3.03293199	<0.001	GO:0034728	nucleosome organization
14	3.01080523	<0.001	GO:0071824	protein-DNA complex subunit organization
14	2.59488342	<0.001	GO:0005694	chromosome
14	2.53934377	<0.001	GO:0034622	cellular macromolecular complex assembly
14	2.44736892	<0.001	GO:0044427	chromosomal part
14	2.42653609	<0.001	GO:0006325	chromatin organization
14	2.40754392	<0.001	GO:0034621	cellular macromolecular complex subunit organization
14	2.32209497	<0.001	GO:0051276	chromosome organization
14	2.28772453	<0.001	GO:0042742	defense response to bacterium
14	2.25069221	<0.001	GO:0065003	macromolecular complex assembly
14	2.21933708	<0.001	GO:0071844	cellular component assembly at cellular level
14	2.20917288	<0.001	GO:0009617	response to bacterium
14	2.16780072	<0.001	GO:0043933	macromolecular complex subunit organization
14	2.06178246	<0.001	GO:0022607	cellular component assembly
14	1.90444862	<0.001	GO:0006996	organelle organization
14	1.81046881	<0.001	GO:0051707	response to other organism
14	1.73024856	<0.001	GO:0043228	non-membrane-bounded organelle
14	1.73024856	<0.001	GO:0043232	intracellular non-membrane-bounded organelle
14	1.7290864	<0.001	GO:0003677	DNA binding
14	1.70656474	<0.001	GO:0071842	cellular component organization at cellular level
14	1.70057755	<0.001	GO:0071841	cellular component organization or biogenesis at cellular level
14	1.63174257	0.003	GO:0009607	response to biotic stimulus
14	1.58401012	<0.001	GO:0016043	cellular component organization
14	1.57915662	<0.001	GO:0071840	cellular component organization or biogenesis
14	1.5547621	0.001	GO:0003676	nucleic acid binding
14	1.50993924	0.003	GO:0032991	macromolecular complex
14	1.44379477	0.013	GO:0006952	defense response
14	1.42519143	0.015	GO:0051704	multi-organism process
14	1.27720411	0.03	GO:0005634	nucleus
14	1.24125313	0.044	GO:0044446	intracellular organelle part

Table S3. Summary of MudPIT proteomic analysis of SV40 LT-interacting proteins

T1 Detected # Out of 5	T1-flag Average dNSAF	T1-flag Detected # Out of 3	T1-HA Average dNSAF	T1-HA Detected # Out of 2	T8 Average dNSAF	T8 Detected # Out of 1	T16 Average dNSAF	T16 Detected # Out of 1	C-TERM-HA Average dNSAF	C-TERM-HA Detected # Out of 2	Control Average dNSAF	Control Detected # Out of 7	Relative Enrichment T1-flag:Control	Relative Enrichment T1-HA:Control	Relative Enrichment C-TERM-HA:Control	NCBI_Gene	Description	Length	MW
5	0.11533	3	0.10054	2	0	0	0	0	0	0	0	0	INF	INF	0	SV40gp6	N-terminal FLAG-HA tagged SV40 large T antigen [Simian virus 40]	708	81624
5	0.07574	3	0.01005	2	0.01333	1	0	0	0	0	0	0	INF	INF	0	BAG2	BAG family molecular chaperone regulator 2 [Homo sapiens]	211	23772
5	0.01641	3	0.00419	2	0	0	0.03171	1	0	0	0	0	INF	INF	0	TP53	cellular tumor antigen p53 isoform a [Homo sapiens]	393	43653
5	0.01166	3	0.00447	2	0	0	0.01712	1	0.00258	1	0	0	INF	INF	INF	FAM111A	hypothetical protein LOC63901 [Homo sapiens]	611	70196
5	0.00752	3	0.00977	2	0	0	0	0	0	0	0	0	INF	INF	0	BAG5	BAG family molecular chaperone regulator 5 isoform b [Homo sapiens] ;E	447	51200
5	0.00381	3	0.00263	2	0.00118	1	0	0	0	0	0	0	INF	INF	0	HSPH1	heat shock protein 105 kDa [Homo sapiens]	858	96865
5	0.00345	3	0.0028	2	0.00037	1	0	0	0	0	0	0	INF	INF	0	STUB1	E3 ubiquitin-protein ligase CHIP [Homo sapiens]	303	34856
5	0.00182	3	0.00254	2	0.00364	1	0	0	0	0	0	0	INF	INF	0	RB1	retinoblastoma-associated protein [Homo sapiens]	928	106159
5	0.00157	3	0.00106	2	0	0	0	0	0	0	0	0	INF	INF	0	FBXW7	F-box/WD repeat-containing protein 7 isoform 1 [Homo sapiens]	707	79663
5	0.00135	3	0.00058	2	0	0	0	0	0	0	0	0	INF	INF	0	KPNA3	importin subunit alpha-3 [Homo sapiens]	521	57811
5	0.00126	3	0.00121	2	0.00254	1	0	0	0	0	0	0	INF	INF	0	BAG3	BAG family molecular chaperone regulator 3 [Homo sapiens]	575	61595
5	0.0003	3	0.00232	2	0	0	0.00571	1	0	0	0	0	INF	INF	0	LGALS3BP	galectin-3-binding protein [Homo sapiens]	585	65331
5	0.00163	3	0.00035	2	0.00116	1	0	0	0	0	2.5E-05	1	65.16	13.96	0	RBL1	retinoblastoma-like protein 1 isoform a [Homo sapiens]	1068	120847
5	0.00401	3	0.00303	2	0.00308	1	0	0	0	0	9.5E-05	1	42.23	31.91	0	HSPA4	heat shock 70 kDa protein 4 [Homo sapiens]	840	94331
4	0.0021	2	0.00039	2	0	0	0	0	0	0	0	0	INF	INF	0	WDR61	WD repeat-containing protein 61 [Homo sapiens]	305	33581
4	0.00202	3	0.00173	1	0	0	0	0	0	0	0	0	INF	INF	0	SRP14	signal recognition particle 14 kDa protein [Homo sapiens]	136	14570
4	0.00123	3	0.00012	1	0	0	0	0	0	0	0	0	INF	INF	0	CCDC77	coiled-coil domain-containing protein 77 isoform a [Homo sapiens]	488	57486
4	0.00091	2	0.00014	2	0	0	0	0	0	0	0	0	INF	INF	0	PAF1	RNA polymerase II-associated factor 1 homolog [Homo sapiens]	531	59976
4	0.00089	3	0.00087	1	0	0	0	0	0	0	0	0	INF	INF	0	DNAJB4	dnaJ homolog subfamily B member 4 [Homo sapiens]	337	37807
4	0.00069	2	0.00116	2	0	0	0	0	0	0	0	0	INF	INF	0	RPL19	60S ribosomal protein L19 [Homo sapiens]	196	23466
4	0.00057	2	0.00206	2	0	0	0	0	0	0	0	0	INF	INF	0	NPM1	nucleophosmin isoform 1 [Homo sapiens] ;nucleophosmin isoform 2 [Hon	294	32575
4	0.00056	2	0.00148	2	0	0	0	0	0	0	0	0	INF	INF	0	TOM1	target of Myb protein 1 isoform 1 [Homo sapiens]	492	53818
4	0.00038	2	0.00056	2	0	0	0	0	0	0	0	0	INF	INF	0	CDK2	cell division protein kinase 2 isoform 2 [Homo sapiens] ;cell division prote	264	30035
4	0.00035	2	0.00042	2	0.00079	1	0	0	0	0	0	0	INF	INF	0	SSR1	translocon-associated protein subunit alpha precursor [Homo sapiens]	286	32235
4	0.00033	3	7.1E-05	1	0.00055	1	0	0	0	0	0	0	INF	INF	0	E2F4	transcription factor E2F4 [Homo sapiens]	413	43960
4	0.00033	3	0.00012	1	0	0	0	0	0	0	0	0	INF	INF	0	CUL1	cullin-1 [Homo sapiens]	776	89678
4	0.0003	2	0.00545	2	0	0	0.0014	1	0	0	0	0	INF	INF	0	RPLP0	60S acidic ribosomal protein P0 [Homo sapiens]	317	34274
4	0.00027	2	0.00026	2	0.00093	1	0	0	0	0	0	0	INF	INF	0	YBX2	Y-box-binding protein 2 [Homo sapiens]	364	38518
4	0.00026	2	0.00055	2	0	0	0	0	0	0	0	0	INF	INF	0	RBM14	RNA-binding protein 14 [Homo sapiens]	669	69492
4	0.00015	2	0.00083	2	0	0	0	0	0	0	0	0	INF	INF	0	PPP1R12A	protein phosphatase 1 regulatory subunit 12A isoform a [Homo sapiens]	1030	115281
4	0.00144	3	0.00071	1	0	0	0	0	0	0	5.1E-05	1	28.1960784	13.941176	0	KPNA4	importin subunit alpha-4 [Homo sapiens]	521	57887
3	0.00347	1	0.00147	2	0	0	0	0	0	0	0	0	INF	INF	0	RPS21	PREDICTED: 40S ribosomal protein S21-like isoform 1 [Homo sapiens] ;4	81	8850
3	0.00267	1	0.00057	2	0.00053	1	0	0	0	0	0	0	INF	INF	0	SDF2	stromal cell-derived factor 2 precursor [Homo sapiens]	211	23026
3	0.00194	1	0.00111	2	0	0	0	0	0	0	0	0	INF	INF	0	C14orf156	SRA stem-loop-interacting RNA-binding protein, mitochondrial precursor	109	12349
3	0.00174	1	0.00131	2	0	0	0	0	0	0	0	0	INF	INF	0	NDUFA4	NADH dehydrogenase [ubiquinone] 1 alpha subcomplex subunit 4 [Homo	81	9370
3	0.00165	1	0.00044	2	0	0	0	0	0	0	0	0	INF	INF	0	PRDX5	peroxiredoxin-5, mitochondrial isoform b precursor [Homo sapiens] ;perox	170	17394
3	0.00156	2	0.00007	1	0	0	0	0	0	0	0	0	INF	INF	0	TRMU	mitochondrial tRNA-specific 2-thiouridylase 1 [Homo sapiens]	421	47745
3	0.00131	2	0.00082	1	0	0	0	0	0	0	0	0	INF	INF	0	HSPBP1	hsp70-binding protein 1 [Homo sapiens]	359	39303
3	0.00121	2	0.00061	1	0	0	0	0	0	0	0	0	INF	INF	0	CDC73	parafibromin [Homo sapiens]	531	60577
3	0.00111	1	0.00053	2	0	0	0	0	0	0	0	0	INF	INF	0	GNAI2	guanine nucleotide-binding protein G(i) subunit alpha-2 isoform 2 [Homo	318	36465
3	0.001	2	0.00099	1	0	0	0	0	0	0	0	0	INF	INF	0	CUEDC1	CUE domain-containing protein 1 [Homo sapiens]	386	42258
3	0.00097	2	5.2E-05	1	0	0	0	0	0	0	0	0	INF	INF	0	RBL2	retinoblastoma-like protein 2 [Homo sapiens]	1139	128367
3	0.00094	2	0.00202	1	0	0	0	0	0	0	0	0	INF	INF	0	TRPT1	tRNA 2'-phosphotransferase 1 isoform 2 [Homo sapiens] ;tRNA 2'-phosph	204	22427
3	0.00081	3	0	0	0	0	0	0	0	0	0	0	INF	0	0	RAE1	mRNA export factor [Homo sapiens]	368	40968
3	0.0008	2	0.0002	1	0	0	0	0	0	0	0	0	INF	INF	0	BRAP	BRCA1-associated protein [Homo sapiens]	592	67305
3	0.00078	2	0.00041	1	0	0	0	0	0	0	0	0	INF	INF	0	SLC25A10	mitochondrial dicarboxylate carrier [Homo sapiens]	287	31282
3	0.0007	2	0.00044	1	0	0	0.00069	1	0.00033	1	0	0	INF	INF	INF	HSPA1L	heat shock 70 kDa protein 1-like [Homo sapiens]	641	70375
3	0.0007	3	0	0	0	0	0	0	0	0	0	0	INF	0	0	TFDP1	transcription factor Dp-1 [Homo sapiens]	410	45070
3	0.00057	2	0.00005	1	0	0	0	0	0	0	0	0	INF	INF	0	ZBED4	zinc finger BED domain-containing protein 4 [Homo sapiens]	1171	130322
3	0.00055	3	0	0	0	0	0	0	0	0	0	0	INF	0	0	NSMCE4A	non-structural maintenance of chromosomes element 4 homolog A isofor	385	44301
3	0.00055	2	3.7E-05	1	0	0	0	0	0	0	0	0	INF	INF	0	DIP2B	disco-interacting protein 2 homolog B [Homo sapiens]	1576	171491
3	0.00052	2	0.00044	1	0	0	0	0	0	0	0	0	INF	INF	0	GPX8	probable glutathione peroxidase 8 [Homo sapiens]	209	23881
3	0.0005	1	7.1E-05	2	0	0	0	0	0	0	0	0	INF	INF	0	PFKM	6-phosphofructokinase, muscle type isoform 1 [Homo sapiens] ;6-phosph	851	93254
3	0.00049	2	0.00011	1	0	0	0	0	0	0	0	0	INF	INF	0	MCM6	DNA replication licensing factor MCM6 [Homo sapiens]	821	92889
3	0.00049	2	0.00043	1	0	0	0	0	0	0	0	0	INF	INF	0	USP7	ubiquitin carboxyl-terminal hydrolase 7 [Homo sapiens]	1102	128302
3	0.00048	2	0.00013	1	0	0	0	0	0	0	0	0	INF	INF	0	E2F3	transcription factor E2F3 [Homo sapiens]	465	49162
3	0.00045	2	0.00014	1	0	0	0	0	0	0	0	0	INF	INF	0	CCNA2	cyclin-A2 [Homo sapiens]	432	48537
3	0.00043	2	0.00016	1	0	0	0	0	0	0	0	0	INF	INF	0	CALD1	caldesmon isoform 4 [Homo sapiens] ;caldesmon isoform 5 [Homo sapie	558	64256
3	0.00038	1	0.00136	2	0	0	0	0	0	0	0	0	INF	INF	0	DYNLL2	dynein light chain 2, cytoplasmic [Homo sapiens]	89	10350
3	0.00038	1	0.00072	2	0	0	0	0	0	0	0	0	INF	INF	0	CTTN	src substrate cortactin isoform a [Homo sapiens] ;src substrate cortactin i	550	61586
3	0.00034	2	7.8E-05	1	0	0	0	0	0	0	0	0	INF	INF	0	CTR9	RNA polymerase-associated protein CTR9 homolog [Homo sapiens]	1173	133502
3	0.00032	1	0.00149	2	0	0	0	0	0	0	0	0	INF	INF	0	RPL13	60S ribosomal protein L13 [Homo sapiens]	211	24261
3	0.00031	2	0.00055	1	0	0	0	0	0	0	0	0	INF	INF	0	OSGEP	probable O-sialoglycoprotein endopeptidase [Homo sapiens]	335	36427
3	0.0003	3	0	0	0	0	0	0	0	0	0	0	INF	0	0	SMC6	structural maintenance of chromosomes protein 6 [Homo sapiens]	1091	126325
3	0.00027	2	0.00051	1	0	0	0	0	0	0	0	0	INF	INF	0	RFC4	replication factor C subunit 4 [Homo sapiens]	363	39682
3	0.00026	2	0.00015	1	0	0	0	0	0	0	0	0	INF	INF	0	IDH3B	isocitrate dehydrogenase [NAD] subunit beta, mitochondrial isoform a pre	385	42184
3	0.00021	1	0.00147	2	0	0	0	0	0	0	0	0	INF	INF	0	RPL12	60S ribosomal protein L12 [Homo sapiens]	165	17819
3	0.00019	3	0	0	0	0	0	0	0	0	0	0	INF	0	0	NDUFS2	NADH dehydrogenase [ubiquinone] iron-sulfur protein 2, mitochondrial is	457	51852
3	0.00018	2	0.0055	1	0	0	0	0	0	0	0	0	INF	INF	0	TWF1	twinfilin-1 [Homo sapiens]	384	43918
3	0.00018	2	0.00075	1	0	0	0	0	0	0	0	0	INF	INF	0	HNRNPL	heterogeneous nuclear ribonucleoprotein L isoform a [Homo sapiens]	589	64133
3	0.00015	2	0.00033	1	0	0	0	0	0	0	0	0	INF	INF	0	NUP50	nuclear pore complex protein Nup50 isoform a [Homo sapiens] ;nuclear p	440	46863
3	0.00013	1	0.00027	2	0	0	0	0	0	0	0	0	INF	INF	0	MYO6	myosin-VI [Homo sapiens]	1285	148713
3	0.00013	2	0.0014	1	0	0	0	0	0	0	0	0	INF	INF	0	SEC23B	protein transport protein Sec23B isoform 1 [Homo sapiens] ;protein trans	767	86479
3	0.00012	2	0.00011	1	0.00026	1	0	0	0	0	0	0	INF	INF	0	MCM4	DNA replication licensing factor MCM4 [Homo sapiens]	863	96558
3	0.00011	2	6.4E-05	1	0	0	0	0	0	0	0	0	INF	INF	0	EIF3CL	eukaryotic translation initiation factor 3 subunit C [Homo sapiens];eukaryo	914	105473
3	0.00011	2	0.00019	1	0	0	0	0	0	0	0	0	INF	INF	0	ZC3H18	zinc finger CCCH domain-containing protein 18 [Homo sapiens]	953	106379
3	0.0001	3	0	0	0	0	0	0	0	0	0	0	INF	0	0	LPCAT1	lysophosphatidylcholine acyltransferase 1 [Homo sapiens]	534	59151
3	9.9E-05	1	0.00011	2	0	0	0	0	0	0	0	0	INF	INF	0	KHSRP	far upstream element-binding protein 2 [Homo sapiens]	711	73115
3	9.4E-05	2	1.4E-05	1	5.3E-05	1	0	0	0	0	0	0	INF	INF	0	DOCK7	dedicator of cytokinesis protein 7 [Homo sapiens]	2109	239420
3	8.3E-05	2	0.0001	1	0	0	0	0	0	0	0	0	INF	INF	0	NOL6	nucleolar protein 6 alpha isoform [Homo sapiens]	1146	127593
3	8.3E-05	1	0.00018	2	0	0	0	0	0	0	0	0	INF	INF	0	PLOD3	procollagen-lysine,2-oxoglutarate 5-dioxygenase 3 precursor [Homo sapi	738	84785
3	8.2E-05	2	0.00034	1	0	0	0	0	0	0	0	0	INF	INF	0	XRN2	5'-3' exoribonuclease 2 [Homo sapiens]	950	108583
3	8.1E-05	1	0.00013	2	0	0	0	0	0	0	0	0	INF	INF	0	TNKS1BP1	182 kDa tankyrase-1-binding protein [Homo sapiens]	1729	181795
3	7.2E-05	2	8.2E-05	1	0	0	8.8E-05	1	0	0	0	0	INF	INF	0	CUL9	cullin-9 [Homo sapiens]	2517	281227
3	6.6E-05	2	0.00118	1	0	0	0	0	0	0	0	0	INF	INF	0	DHX15	putative pre-mRNA-splicing factor ATP-dependent RNA helicase DHX15	795	90933
3	6.3E-05	2	0.00012	1	0	0	0	0	0	0	0	0	INF	INF	0	POP1	ribonucleases P/MRP protein subunit POP1 [Homo sapiens]	1024	114709
3	3.8E-05	2	2.8E-05	1	0	0	0	0	0	0	0	0	INF	INF	0	RANBP2	E3 SUMO-protein ligase RanBP2 [Homo sapiens]	3224	358201
3	3.7E-05	1	9.1E-05	2	0	0	0	0	0	0	0	0	INF	INF	0	TJP1	tight junction protein ZO-1 isoform b [Homo sapiens] ;tight junction protei	1668	186965
3	2.7E-05	1	0.00057	2	0	0	0	0	0	0	0	0	INF	INF	0	DHX9	ATP-dependent RNA helicase A [Homo sapiens]	1270	140958
3	0.00194	1	0.00131	2	0	0	0	0	0	0	4.3E-05	1	45.1162791	30.372093	0	RPA1	replication protein A 70 kDa DNA-binding subunit [Homo sapiens]	616	68138

Table S4. iTRAQ-based quantitative analysis of full-length and sub-genomic fragments of SV40 LT. The numbers correspond to the relative enrichment factor measured for proteins detected in experimental and control IPs.

Protein Description	Gene Symbol	Gene IDs	T1_Exp1	T1_Exp2	T8_Exp1	T16_Exp1	T16_Exp2
retinoblastoma-like protein 1 isoform a	RBL1	5933	44.2	13.5	3.5	-	-
heat shock cognate 71 kDa protein isoform 1	HSPA8	3312	37.0	38.5	34.5	-	-
cellular tumor antigen p53 isoform b	TP53	7157	28.8	22.3	-	14.7	13.0
retinoblastoma-associated protein	RB1	5925	28.0	37.0	-	-	-
hypothetical protein LOC63901	FAM111A	63901	25.5	27.9	-	6.5	3.7
heat shock 70 kDa protein 6	HSPA6	3310	22.4	-	-	-	-
large T antigen	SV40-LT	1489531	20.7	34.0	32.2	39.6	38.4
BAG family molecular chaperone regulator 2	BAG2	9532	18.5	35.8	25.9	-	-
BAG family molecular chaperone regulator 5 isoform b	BAG5	9529	13.7	61.0	-	-	-
coiled-coil domain-containing protein 77 isoform a	CCDC77	84318	11.1	-	-	-	-
E3 ubiquitin-protein ligase CHIP	STUB1	10273	8.7	17.5	8.6	-	-
dnaJ homolog subfamily C member 7 isoform 1	DNAJC7	7266	6.8	6.5	-	-	-
dnaJ homolog subfamily B member 4	DNAJB4	11080	3.9	-	11.8	-	-
periphilin-1 isoform 1	PPHLN1	51535	3.5	5.0	0.6	-	-
signal recognition particle 9 kDa protein isoform 2	SRP9	6726	3.4	2.1	2.5	-	-
U5 small nuclear ribonucleoprotein 200 kDa helicase	SNRNP200	23020	3.3	3.2	1.6	-	-
40S ribosomal protein S2	RPS2	6187	3.0	2.7	1.6	-	-
pre-mRNA-processing factor 19	PRPF19	27339	2.9	6.5	-	-	-
splicing factor, arginine/serine-rich 1 isoform 1	SRSF1	6426	2.9	2.5	1.4	-	0.9
lamin-A/C isoform 3	LMNA	4000	2.6	2.9	2.1	-	-
peptidyl-prolyl cis-trans isomerase B precursor	PPIB	5479	2.5	0.7	-	-	-
rRNA 2'-O-methyltransferase fibrillarin	FBL	2091	2.3	2.2	2.1	-	-
heterogeneous nuclear ribonucleoprotein H	HNRNPH1	3187	2.2	1.9	1.8	-	-
cleavage and polyadenylation specificity factor subunit 6	CPSF6	11052	2.1	1.9	-	-	-
hypothetical protein LOC144097	C11orf84	144097	2.1	-	1.2	-	-
40S ribosomal protein S6	RPS6	6194	1.9	1.2	1.6	-	-
probable ATP-dependent RNA helicase DDX5	DDX5	1655	1.9	1.4	2.0	0.8	1.2
60S ribosomal protein L18	RPL18	6141	1.9	1.2	1.7	-	-
serine/threonine-protein phosphatase PP1-gamma catalytic subunit	PPP1CC	5501	1.8	-	-	-	-
40S ribosomal protein S29 isoform 2	RPS29	6235	1.8	1.4	-	-	-
splicing factor U2AF 65 kDa subunit isoform b	U2AF2	11338	1.8	1.8	1.9	1.8	-
U4/U6 small nuclear ribonucleoprotein Prp31	PRPF31	26121	1.8	-	1.5	-	-
ADP/ATP translocase 2	SLC25A5	292	1.7	2.4	3.0	-	-
tubulin alpha-8 chain isoform 1	TUBA8	51807	1.7	-	-	-	-
heterogeneous nuclear ribonucleoprotein A/B isoform b	HNRNPAB	3182	1.7	-	2.7	-	-
heterogeneous nuclear ribonucleoprotein U isoform a	HNRNPU	3192	1.7	-	1.8	-	-
rho-related GTP-binding protein RhoG precursor	RHOG	391	1.7	-	-	-	-
tubulin alpha-1B chain	TUBA1B	10376	1.6	3.4	2.1	4.1	1.8
tubulin alpha-1C chain	TUBA1C	84790	1.6	3.5	2.4	-	-
40S ribosomal protein S4, X isoform X isoform	RPS4X	6191	1.6	1.7	1.3	1.6	-
ADP/ATP translocase 3	SLC25A6	293	1.6	-	3.3	-	-
galectin-1	LGALS1	3956	1.6	1.6	2.5	0.5	0.4
ribosomal L1 domain-containing protein 1	RSL1D1	26156	1.5	1.8	2.6	2.2	1.4
ATP-dependent RNA helicase DDX3X isoform 1	DDX3X	1654	1.5	1.5	2.1	-	-
tubulin beta-2A chain	TUBB2A	7280	1.5	-	-	-	-
60S ribosomal protein L23	RPL23	9349	1.5	1.6	1.8	-	3.3
40S ribosomal protein S27-like	RPS27L	51065	1.5	-	-	-	-
heterogeneous nuclear ribonucleoprotein A1 isoform a	HNRNPA1	3178	1.5	-	-	-	-
60S ribosomal protein L10	RPL10	6134	1.5	1.4	1.7	-	0.7
60S ribosomal protein L27a	RPL27A	6157	1.5	1.2	1.7	-	-
extended synaptotagmin-1 isoform 1	ESYT1	23344	1.5	-	-	-	-
nexilin isoform 2	NEXN	91624	1.4	1.8	3.3	-	-
OCIA domain-containing protein 2 isoform 1	OCIAD2	132299	1.4	3.0	-	-	-
heterogeneous nuclear ribonucleoprotein F	HNRNPF	3185	1.4	2.5	3.4	-	-
alpha-actinin-4	ACTN4	81	1.4	2.2	3.0	1.2	0.8
40S ribosomal protein S3a	RPS3A	6189	1.4	1.3	1.6	-	-
DNA excision repair protein ERCC-6	ERCC6	2074	1.3	-	-	-	-
alpha-actinin-1 isoform b	ACTN1	87	1.3	2.5	3.6	1.0	0.9
40S ribosomal protein S5	RPS5	6193	1.3	1.4	2.2	2.8	0.8
60S ribosomal protein L32	RPL32	6161	1.3	1.0	1.9	-	-
filamin-A isoform 2	FLNA	2316	1.2	2.6	1.0	1.7	0.6
tricarboxylate transport protein, mitochondrial precursor	SLC25A1	6576	1.2	3.9	1.8	-	-
kinesin-like protein KIF11	KIF11	3832	1.2	1.6	0.5	1.3	1.1
60S ribosomal protein L5	RPL5	6125	1.2	1.0	1.7	-	1.1
60S ribosomal protein L36	RPL36	25873	1.2	1.0	1.6	-	-
40S ribosomal protein S11	RPS11	6205	1.2	1.2	1.4	-	-
60S ribosomal protein L14	RPL14	9045	1.2	1.0	1.9	-	-
60S ribosomal protein L4	RPL4	6124	1.2	1.1	1.6	-	-
40S ribosomal protein S8	RPS8	6202	1.2	0.9	1.4	-	-
ATP-dependent RNA helicase A	DHX9	1660	1.1	1.3	2.1	-	-
60S ribosomal protein L30	RPL30	6156	1.1	0.8	1.8	-	1.4
60S ribosomal protein L10a	RPL10A	4736	1.1	1.1	1.9	-	1.4
serine/threonine-protein kinase 38	STK38	11329	1.1	1.7	0.8	1.3	1.3
trinucleotide repeat-containing gene 6A protein	TNRC6A	27327	1.1	0.7	-	-	-
myosin-Ic isoform b	MYO1C	4641	1.1	1.8	1.6	-	-
plectin isoform 1c	PLEC	5339	1.1	1.0	2.4	-	-
40S ribosomal protein S20 isoform 2	RPS20	6224	1.0	0.7	-	-	-
tyrosine-protein kinase JAK1	JAK1	3716	1.0	-	-	-	-
POTE ankyrin domain family member E	POTEE	445582	1.0	-	-	-	-
60S ribosomal protein L12	RPL12	6136	1.0	0.8	1.8	1.1	-
60S ribosomal protein L3 isoform b	RPL3	6122	0.9	1.1	1.8	-	-
cellular nucleic acid-binding protein isoform 3	CNBP	7555	0.9	-	-	-	-
F-actin-capping protein subunit beta	CAPZB	832	0.9	1.4	1.8	-	0.6
60S ribosomal protein L18a	RPL18A	6142	0.9	0.7	1.6	-	-
40S ribosomal protein SA	RPSA	3921	0.9	-	-	-	-
pyruvate dehydrogenase E1 component subunit beta, mitochondrial isoform 2 precursor	PDHB	5162	0.9	0.4	-	-	-

Table S4. iTRAQ-based quantitative analysis of full-length and sub-genomic fragments of SV40 LT. The numbers correspond to the relative enrichment factor measured for proteins detected in experimental and control IPs.

40S ribosomal protein S3	RPS3	6188	0.8	0.7	2.4	-	-
transitional endoplasmic reticulum ATPase	VCP	7415	0.8	1.0	1.9	0.9	-
dehydrogenase/reductase SDR family member 2 isoform 2	DHRS2	10202	0.7	2.1	2.4	0.9	1.0
BTB/POZ domain-containing protein KCTD5	KCTD5	54442	0.7	1.5	0.5	2.0	1.0
methylosome protein 50	WDR77	79084	0.7	1.0	0.6	-	-
drebrin isoform a	DBN1	1627	0.7	2.2	2.9	-	1.5
myosin-Ib isoform 2	MYO1B	4430	0.7	1.1	2.8	-	-
spectrin beta chain, erythrocyte isoform a	SPTB	6710	0.7	-	-	-	-
RNA-binding protein 10 isoform 2	RBM10	8241	0.7	1.6	1.6	-	1.0
vimentin	VIM	7431	0.7	1.0	3.0	-	1.8
ATP synthase subunit d, mitochondrial isoform a	ATP5H	10476	0.7	1.1	-	-	-
T-complex protein 1 subunit epsilon	CCT5	22948	0.7	-	2.1	-	-
thy-1 membrane glycoprotein preproprotein	THY1	7070	0.7	-	2.1	-	-
NADH dehydrogenase	NDUFB9	4715	0.7	-	-	-	-
T-complex protein 1 subunit eta isoform b	CCT7	10574	0.6	-	-	-	-
3-hydroxyacyl-CoA dehydrogenase type-2 isoform 2	HSD17B10	3028	0.6	0.5	-	-	-
annexin A2 isoform 1	ANXA2	302	0.6	0.7	2.9	1.9	0.8
leucine-rich repeat-containing protein 59	LRRC59	55379	0.6	0.7	1.9	-	-
coronin-1C isoform 1	CORO1C	23603	0.6	2.2	8.5	1.0	1.1
protein arginine N-methyltransferase 5 isoform a	PRMT5	10419	0.6	1.0	0.7	-	-
guanine nucleotide-binding protein G(I)/G(S)/G(T) subunit beta-2	GNB2	2783	0.6	1.2	1.9	-	0.7
myosin-9	MYH9	4627	0.6	1.3	1.0	-	-
elongation factor 1-gamma	EEF1G	1937	0.5	0.7	-	-	-
pyrroline-5-carboxylate reductase 1, mitochondrial isoform 1	PYCR1	5831	0.5	-	-	-	-
citrate synthase, mitochondrial precursor	CS	1431	0.5	-	-	-	-
malate dehydrogenase, mitochondrial precursor	MDH2	4191	0.5	0.1	-	-	-
protein ETHE1, mitochondrial precursor	ETHE1	23474	0.5	6.5	-	-	-
peptidyl-prolyl cis-trans isomerase A	PPIA	5478	0.4	0.2	-	-	-
myosin light chain 6B	MYL6B	140465	0.4	-	-	-	-
serine hydroxymethyltransferase, mitochondrial isoform 3	SHMT2	6472	0.4	0.4	-	-	-
60 kDa heat shock protein, mitochondrial	HSPD1	3329	0.3	0.3	3.6	-	1.4
flotillin-1	FLOT1	10211	-	-	2.0	-	-
heterogeneous nuclear ribonucleoprotein D0 isoform d	HNRNPD	3184	-	1.9	-	-	-
F-actin-capping protein subunit alpha-1	CAPZA1	829	-	1.2	1.8	-	0.4
14-3-3 protein beta/alpha	YWHAB	7529	-	-	3.6	-	-
BTB/POZ domain-containing protein KCTD17	KCTD17	79734	-	1.7	0.5	2.3	0.9
WD40 repeat-containing protein SMU1	SMU1	55234	-	2.7	-	-	-
spectrin beta chain, brain 2	SPTBN2	6712	-	-	3.3	-	-
V-type proton ATPase subunit d 1	ATP6V0D1	9114	-	-	3.2	-	-
mRNA turnover protein 4 homolog	MRTO4	51154	-	-	2.7	-	-
FACT complex subunit SPT16	SUPT16H	11198	-	-	2.2	-	-
X-ray repair cross-complementing protein 5	XRCC5	7520	-	1.6	4.2	-	-
14-3-3 protein zeta/delta	YWHAZ	7534	-	1.1	2.9	-	-
LIM domain only protein 7 isoform 1	LMO7	4008	-	2.0	-	-	-
CD44 antigen isoform 4 precursor	CD44	960	-	-	1.6	-	-
14-3-3 protein theta	YWHAQ	10971	-	-	3.3	-	-
PREDICTED: similar to hCG2040270	SNRPD2	6633	-	2.3	1.7	-	-
heterogeneous nuclear ribonucleoprotein A0	HNRNPA0	10949	-	-	1.3	-	-
clathrin light chain B isoform b	CLTB	1212	-	-	3.4	-	-
zyxin	ZYX	7791	-	2.5	-	-	-
heterogeneous nuclear ribonucleoprotein A3	HNRNPA3	220988	-	1.5	-	-	-
PREDICTED: similar to protein expressed in prostate, ovary, testis, and placenta 2	POTEJ	653781	-	-	3.0	-	-
116 kDa U5 small nuclear ribonucleoprotein component isoform b	EFTUD2	9343	-	-	1.7	-	-
spectrin alpha chain, brain isoform 3	SPTAN1	6709	-	2.5	3.1	-	-
small nuclear ribonucleoprotein E	SNRPE	6635	-	-	1.8	-	-
protein phosphatase 1B isoform 5	PPM1B	5495	-	-	0.4	-	-
guanine nucleotide-binding protein G(I)/G(S)/G(O) subunit gamma-12 precursor	GNG12	55970	-	1.2	2.0	-	-
T-complex protein 1 subunit alpha isoform a	TCP1	6950	-	-	1.8	-	-
actin-binding LIM protein 1 isoform c	ABLIM1	3983	-	-	1.1	-	-
elongation factor 1-alpha 1	EEF1A1	1915	-	1.4	1.9	-	1.3
probable ATP-dependent RNA helicase DDX17 isoform 1	DDX17	10521	-	1.5	-	-	-
proliferating cell nuclear antigen	PCNA	5111	-	-	5.7	-	-
tubulin alpha-1A chain	TUBA1A	7846	-	-	-	5.6	-
hypoxia up-regulated protein 1 precursor	HYOU1	10525	-	-	3.9	-	-
semaphorin-7A isoform 1 preproprotein	SEMA7A	8482	-	-	2.1	-	-
urokinase plasminogen activator surface receptor isoform 3 precursor	PLAUR	5329	-	-	2.9	-	-
actin-related protein 2/3 complex subunit 4 isoform a	ARPC4	10093	-	-	4.0	-	-
desmoglein-2 preproprotein	DSG2	1829	-	-	3.1	-	-
retinoic acid-induced protein 3	GPRC5A	9052	-	-	2.4	-	-
histone deacetylase complex subunit SAP18	SAP18	10284	-	9.6	-	-	-
splicing factor 3A subunit 3	SF3A3	10946	-	2.4	1.4	-	-
luc7-like protein 3	LUC7L3	51747	-	2.0	-	-	-
protein phosphatase 1 regulatory subunit 12A isoform a	PPP1R12A	4659	-	2.1	-	-	-
cell division protein kinase 2 isoform 1	CDK2	1017	-	30.7	-	-	-
charged multivesicular body protein 5 isoform 2	CHMP5	51510	-	-	2.6	-	-
60S ribosomal protein L28 isoform 5	RPL28	6158	-	1.4	-	-	-
highly divergent homeobox isoform 1	HDX	139324	-	-	1.6	-	-
PREDICTED: similar to myosin regulatory light chain MRCL2	LOC391722	391722	-	1.4	1.2	-	-
interleukin enhancer-binding factor 3 isoform c	ILF3	3609	-	-	2.5	-	-
serine/threonine-protein phosphatase PP1-alpha catalytic subunit isoform 3	PPP1CA	5499	-	1.6	3.3	-	-
cofilin-1	CFL1	1072	-	2.8	9.8	-	-
transient receptor potential cation channel subfamily M member 2	TRPM2	7226	-	-	-	0.9	-
nucleolar protein 56	NOP56	10528	-	-	2.7	-	-
spectrin beta chain, brain 1 isoform 2	SPTBN1	6711	-	2.1	3.1	-	0.8
replication protein A 70 kDa DNA-binding subunit	RPA1	6117	-	-	3.0	-	-
14-3-3 protein epsilon	YWHAE	7531	-	-	3.0	-	-
voltage-dependent calcium channel subunit alpha-2/delta-1	CACNA2D1	781	-	-	2.1	-	-

Table S4. iTRAQ-based quantitative analysis of full-length and sub-genomic fragments of SV40 LT. The numbers correspond to the relative enrichment factor measured for proteins detected in experimental and control IPs.

poly(rC)-binding protein 3 isoform 2	PCBP3	54039	-	1.0	2.6	-	-
catenin beta-1	CTNNB1	1499	-	-	2.5	-	-
actin-related protein 3	ACTR3	10096	-	1.1	-	-	-
coronin-2A	CORO2A	7464	-	-	3.3	-	-
casein kinase II subunit beta	CSNK2B	1460	-	-	2.8	-	-
clathrin heavy chain 1	CLTC	1213	-	2.2	2.3	-	-
LIM domain and actin-binding protein 1 isoform b	LIMA1	51474	-	1.4	2.5	-	-
rho-related GTP-binding protein RhoC precursor	RHOC	389	-	1.2	-	-	-
heat shock 70 kDa protein 1A/1B	HSPA1A	3303	-	17.1	11.6	-	-
60S acidic ribosomal protein P0	RPLP0	6175	-	0.9	1.5	1.7	1.1
putative pre-mRNA-splicing factor ATP-dependent RNA helicase DHX15	DHX15	1665	-	-	1.1	-	-
heterogeneous nuclear ribonucleoprotein A1-like 2	HNRNPA1L2	144983	-	-	2.2	-	-
78 kDa glucose-regulated protein precursor	HSPA5	3309	-	-	1.9	-	-
guanine nucleotide-binding protein G(I)/G(S)/G(T) subunit beta-1	GNB1	2782	-	1.2	2.0	-	-
melanotransferrin isoform 1 precursor	MFI2	4241	-	-	2.9	-	-
splicing factor 3B subunit 3	SF3B3	23450	-	-	1.5	-	-
small nuclear ribonucleoprotein Sm D3	SNRPD3	6634	-	2.5	1.0	-	0.9
major vault protein	MVP	9961	-	-	2.1	-	-
heterogeneous nuclear ribonucleoprotein L isoform b	HNRNPL	3191	-	-	2.3	-	-
methylosome subunit pICln	CLNS1A	1207	-	-	0.8	-	-
T-complex protein 1 subunit delta	CCT4	10575	-	-	2.1	-	-
filamin-B isoform 4	FLNB	2317	-	1.7	1.5	-	0.8
trifunctional enzyme subunit alpha, mitochondrial precursor	HADHA	3030	-	-	3.2	-	-
RING finger protein 219	RNF219	79596	-	-	1.7	-	-
protein flightless-1 homolog	FLII	2314	-	1.6	1.6	-	-
heat shock 70 kDa protein 4	HSPA4	3308	-	-	11.4	-	-
60S ribosomal protein L37a	RPL37A	6168	-	0.8	-	-	-
splicing factor U2AF 35 kDa subunit isoform c	U2AF1	7307	-	2.0	2.0	-	-
glyceraldehyde-3-phosphate dehydrogenase	GAPDH	2597	-	1.0	1.8	-	-
actin-related protein 2 isoform a	ACTR2	10097	-	-	3.0	-	-
dolichyl-diphosphooligosaccharide--protein glycosyltransferase 48 kDa subunit precursor	DDOST	1650	-	1.4	-	-	-
guanine nucleotide-binding protein G(i) subunit alpha-2 isoform 1	GNAI2	2771	-	-	2.2	-	-
heterogeneous nuclear ribonucleoprotein R isoform 2	HNRNPR	10236	-	1.4	2.4	-	-
60S ribosomal protein L7	RPL7	6129	-	1.2	-	-	-
endothelial protein C receptor precursor	PROCR	10544	-	-	2.9	-	-
glypican-1 precursor	GPC1	2817	-	-	1.8	-	-
heterogeneous nuclear ribonucleoprotein U-like protein 1 isoform a	HNRNPUL1	11100	-	-	2.7	-	-
flotillin-2	FLOT2	2319	-	-	2.0	-	-
cytospin-A	SPECC1L	23384	-	-	1.2	-	-
WD repeat-containing protein 1 isoform 1	WDR1	9948	-	-	6.4	-	-
U2-associated protein SR140	SR140	23350	-	-	-	-	0.9
T-complex protein 1 subunit gamma isoform a	CCT3	7203	-	-	1.7	-	-
latent-transforming growth factor beta-binding protein 1 isoform 5 precursor	LTBP1	4052	-	1.7	1.2	-	-
nucleolar RNA helicase 2	DDX21	9188	-	-	2.5	-	-
Golgi-associated plant pathogenesis-related protein 1	GLIPR2	152007	-	-	1.3	-	-
ornithine aminotransferase, mitochondrial isoform 1 precursor	OAT	4942	-	0.2	-	-	-
DNA-dependent protein kinase catalytic subunit isoform 1	PRKDC	5591	-	2.4	3.5	-	-
galectin-3-binding protein	LGALS3BP	3959	-	-	7.4	-	-
40S ribosomal protein S12	RPS12	6206	-	0.8	3.7	-	-
splicing factor, arginine/serine-rich 9	SRSF9	8683	-	4.6	-	-	-
eukaryotic translation initiation factor 5A-1 isoform B	EIF5A	1984	-	-	2.3	-	-
stress-70 protein, mitochondrial precursor	HSPA9	3313	-	1.2	8.3	-	-
elongation factor Tu, mitochondrial precursor	TUFM	7284	-	1.2	2.7	-	-
40S ribosomal protein S17	RPS17	6218	-	1.1	2.1	-	-
heterogeneous nuclear ribonucleoprotein U-like protein 2	HNRNPUL2	221092	-	-	1.8	-	-
enhancer of rudimentary homolog	ERH	2079	-	3.2	0.5	4.8	1.1
NADH dehydrogenase	NDUFA4	4697	-	1.9	3.6	-	-
nucleosome assembly protein 1-like 1	NAP1L1	4673	-	-	3.1	-	-
tubulin alpha-3E chain	TUBA3E	112714	-	-	-	-	2.7
transcription factor Dp-1	TFDP1	7027	-	18.2	-	-	-
dynactin subunit 5	DCTN5	84516	-	3.4	-	-	-
splicing factor 3B subunit 5	SF3B5	83443	-	-	1.3	-	-
guanine nucleotide-binding protein subunit beta-4	GNB4	59345	-	-	2.1	-	-
actin, cytoplasmic 2	ACTG1	71	-	1.6	-	0.9	0.5
TATA-binding protein-associated factor 2N isoform 2	TAF15	8148	-	-	-	1.4	1.2
fragile X mental retardation syndrome-related protein 1 isoform b	FXR1	8087	-	1.0	-	-	-
60S ribosomal protein L9	RPL9	6133	-	1.0	1.8	-	-
metalloreductase STEAP3 isoform b	STEAP3	55240	-	10.1	-	-	-
ribose-phosphate pyrophosphokinase 1	PRPS1	5631	-	-	0.3	-	-
tubulin beta-2C chain	TUBB2C	10383	-	3.8	2.0	4.5	4.8
60S ribosomal protein L22 proprotein	RPL22	6146	-	0.8	-	-	0.4
bifunctional aminoacyl-tRNA synthetase	EPRS	2058	-	-	3.0	-	-
LIM and calponin homology domains-containing protein 1 isoform e	LIMCH1	22998	-	2.0	-	-	-
60S acidic ribosomal protein P1 isoform 2	RPLP1	6176	-	-	1.5	-	-
myosin-Va isoform 2	MYO5A	4644	-	-	1.9	-	-
tropomyosin alpha-4 chain isoform 2	TPM4	7171	-	1.5	1.1	-	-
fibronectin isoform 1 preproprotein	FN1	2335	-	-	3.8	-	-
FACT complex subunit SSRP1	SSRP1	6749	-	-	2.1	-	-
ribosomal RNA processing protein 1 homolog B	RRP1B	23076	-	-	3.2	-	-
E3 SUMO-protein ligase NSE2	NSMCE2	286053	-	3.2	-	-	-
barrier-to-autointegration factor	BANF1	8815	-	2.7	-	-	-
T-complex protein 1 subunit theta	CCT8	10694	-	-	2.3	-	-
inosine-5'-monophosphate dehydrogenase 2	IMPDH2	3615	-	-	3.4	-	-
60S ribosomal protein L7a	RPL7A	6130	-	1.4	2.3	-	-
calcium homeostasis endoplasmic reticulum protein	CHERP	10523	-	-	1.4	-	-
ras-related C3 botulinum toxin substrate 1 isoform Rac1	RAC1	5879	-	-	3.2	-	-
E3 ubiquitin-protein ligase TRIM21	TRIM21	6737	-	1.9	1.1	-	-

Table S4. iTRAQ-based quantitative analysis of full-length and sub-genomic fragments of SV40 LT. The numbers correspond to the relative enrichment factor measured for proteins detected in experimental and control IPs.

putative ribosomal RNA methyltransferase NOP2	NOP2	4839	-	-	2.6	-	-
elongation factor 1-delta isoform 4	EEF1D	1936	-	-	1.2	-	-
AP-2 complex subunit alpha-1 isoform 2	AP2A1	160	-	2.1	1.8	-	-
heterogeneous nuclear ribonucleoprotein M isoform b	HNRNPM	4670	-	1.7	2.1	-	-
D-3-phosphoglycerate dehydrogenase	PHGDH	26227	-	-	2.4	-	-
neuroblast differentiation-associated protein AHNAK isoform 1	AHNAK	79026	-	-	3.0	-	-
F-box/WD repeat-containing protein 7 isoform 3	FBXW7	55294	-	27.0	-	-	-
fascin	FSCN1	6624	-	-	-	-	0.2
mitochondrial ribonuclease P protein 1	RG9MTD1	54931	-	0.8	-	-	-
filamin-C isoform b	FLNC	2318	-	1.5	1.6	-	-
guanine nucleotide-binding protein G(k) subunit alpha	GNAI3	2773	-	0.7	-	-	-
gelsolin isoform a precursor	GSN	2934	-	3.1	2.1	-	-
DNA topoisomerase 1	TOP1	7150	-	-	2.7	-	-
dnaJ homolog subfamily B member 1	DNAJB1	3337	-	10.1	-	-	-
neurotrimin isoform 1	NTM	50863	-	-	2.3	-	-
ruvB-like 1	RUVBL1	8607	-	-	4.3	-	-
coronin-2B isoform 2	CORO2B	10391	-	1.4	3.2	-	-
heat shock protein HSP 90-beta	HSP90AB1	3326	-	-	3.8	-	-
structural maintenance of chromosomes protein 5	SMC5	23137	-	16.0	-	-	-
CD59 glycoprotein preproprotein	CD59	966	-	1.7	2.3	-	-
cofilin-2	CFL2	1073	-	-	4.8	-	-
pyruvate kinase isozymes M1/M2 isoform M1	PKM2	5315	-	-	2.2	-	-
myosin light polypeptide 6 isoform 2	MYL6	4637	-	1.4	1.1	-	0.8
60S ribosomal protein L24	RPL24	6152	-	1.8	1.6	-	-
actin, cytoplasmic 1	ACTB	60	-	-	1.7	-	-
splicing factor 3B subunit 1 isoform 1	SF3B1	23451	-	-	1.3	-	-

3. Supplementary experimental procedures

Viral DNA. Viral DNA was prepared by excision of the viral genome from the pBluescript vector by BamHI digestion; linearized DNA was re-circularized by overnight ligation with T4 ligase at 16°C. The SV40 point mutant T701A cannot be phosphorylated at threonine 701 and fails to associate with FBXW7, while the SV40 mutant K697R cannot be acetylated by CBP/p300 [1-3]. The host range SV40 mutant HR684 [2] contains a single nucleotide point substitution of G to A at nucleotide 2765 resulting in the replacement of glutamine 685 with a stop codon. The host range SV40 mutant dl1066 contains a frameshift mutation that results in the loss of the C-terminal 38 residues of LT and the addition of 8 missense residues [4].

Quantitative RT-PCR. Cells were harvested in TriZOL (Invitrogen) and RNA was extracted. The RNA was then purified using an RNEasy kit (Qiagen) and cDNA was made using the SuperScript III kit (Invitrogen), both according to the manufacturers' protocols. Real-time PCR quantification of FAM111A depletion was performed in triplicate using a TaqMan probe/primer set specific to FAM111A (Applied Biosystems) and a Stratagene Mx3005P instrument. Beta-actin was used as an internal reference standard (Applied Biosystems product number 4310881E).

Plaque assays. 6-well plates of CV-1P cells were plated the previous day to be 95% confluent on the day of transfection. Cells were transfected with 1 ng of wild type SV40 and either 3 ng or 6 ng of the host range mutant virus re-circularized viral DNA using Lipofectamine 2000 (Invitrogen) according to the manufacturer's protocol. Five hours post-transfection, the transfection mixture was aspirated and the cells were overlaid with

2 mL of a 1:1 mixture of melted 1.8% BactoAgar (BD Biosciences) cooled to 45°C and 2x modified Eagle's medium (MEM) without phenol red containing 10% serum and 200 U of penicillin per mL, and 200 µg of streptomycin per mL at 37°C. The cells were overlaid every 3 days with 2 mL 1:1 BactoAgar:MEM mixture until day 6 when neutral red was added to the this mixture at a concentration of 75 µg/mL. Plaques were visualized and counted on days 7-10. All plaque assays were carried out at 37°C.

Single burst assay. The single burst assay was modified from [5]). CV-1P cells stably expressing empty vector or FAM111A shRNAs were seeded onto 35 mm dishes (3 x 10^5 cells/plate) and infected with wild type or host range dl1066 viral stocks that were previously tittered on BSC40 cells at a multiplicity of infection of 3. At various time points after infection (day 0, 1, 2, and 3) the infected cells (including their media) were frozen and thawed three times, and the virus titer was determined by plaque assay on BSC40 cells at 37°C.

siRNA and shRNA. The sequences of siRNA and shRNA targeting FAM111A used were: FAM111A siRNA: GGUCAAUGUGUAAGGGUGA; FAM111A shRNA1: TGCTGTTGACAGTGAGCGCGGTATGAAGAAGTATTTGTAATAGTGAAGCCACAGAT GTATTACAAATACTTCTTCATACCATGCCTACTGCCTCGGA; FAM111A shRNA 2: TGCTGTTGACAGTGAGCGCCCAGAGTATGTCCATATGTATTAGTGA AGCCACAGATGTAATACATATGGACATACTCTGGATGCCTACTGCCTCGGA.

Large Scale Immunoprecipitation. Cells were rinsed with PBS containing protease and phosphatase inhibitors (Calbiochem) and collected by scraping from the plates followed by centrifugation at 1,000 x *g* for 5 minutes. Cell pellets were frozen on dry ice and stored at -80°C until analysis. For MudPIT analysis cell pellets were thawed on ice

and extracted using ice-cold extracting buffer (50 mM TRIS-HCl pH 8.0, 150 mM NaCl, 0.5% NP-40, 0.5 mM EDTA) supplemented with protease and phosphatase inhibitors. Extracts were clarified by centrifugation at 20,000 x *g* for 30 minutes and used for large scale immunoprecipitations as described [6,7]. For iTRAQ analysis cells were processed as previously described [8].

Quantitative LC/MS methods. Mock and LT IP were processed in parallel as follow: Proteins in the FLAG elutions were denatured with 0.1% RapiGest (WATERS), cysteines were reduced with 10 mM DTT for 30 minutes at 56°C and proteins subsequently digested overnight at 37°C using 5 µg of trypsin. Tryptic peptides were desalted on C18 reverse phase chromatography in a batch mode, eluted with 50 µl of 40% acetonitrile / 0.1% TFA and dried down by vacuum centrifugation. Cysteine-containing peptides were captured on thiol-activated sepharose 4B beads (20 µl packed volume) for 1 hour at RT in a volume of 40 µl of 0.5 M triethyl-ammonium bicarbonate (TEAB). After two washes using 100 µl of 0.5 M TEAB, beads were re-suspended in 40 µl of 0.5 M TEAB and labeled using 70 µl of iTRAQ reagent for 1 hour at RT with agitation. After two washes using 100 µl of 0.5 M TEAB, beads from the mock and LT samples were combined, captured peptides were eluted using 40 µl of 10 mM DTT and immediately alkylated with 20 mM iodoacetamide. Peptides were separated using a 0 to 40% acetonitrile gradient on an inline nano-acquity system (WATERS) and analyzed on a QSTAR Elite mass spectrometer (Applied Biosystems) using a dual scan method [9]. A Digital PicoView ESI source (New Objective, Woburn, MA) was used to facilitate positioning of the emitter tip at the orifice of the mass spectrometer during each analysis. Data files were extracted using ProteinPilot software (Applied Biosystems). Peptide sequences and quantitative information (iTRAQ reporter ion peak area) were independently retrieved and merged using in-house multiplierz script. iTRAQ ratio for all peptides with a score above 10 were

summed for each protein and the log2 of the resulting value calculated.

Multidimensional protein identification technology (MudPIT) analysis. TCA-precipitated proteins were resuspended in 30 µl of 100 mM Tris-HCl, pH 8.5, 8 M urea, reduced with 5 mM TCEP (Tris(2-Carboxylethyl)-Phosphine Hydrochloride, Pierce), and alkylated with 10 mM IAM (Iodoacetamide, Sigma). As described in [10], a two-step digestion procedure was used. Endoproteinase Lys-C (Roche) was added to 0.5 µg for at least 6 hours at 37°C, then the sample was diluted to 2 M urea with 100 mM Tris-HCl, pH 8.5. Calcium chloride was added to 2 mM and the digestion with 0.5 µg trypsin (Promega) was let to proceed overnight at 37°C while shaking. The reaction was quenched by adding formic acid to 5% and the peptide mixture was loaded onto a 100 µm fused silica microcapillary column packed with 8 cm of reverse phase material (Aqua, Phenomenex), followed with 3 cm of 5-µm Strong Cation Exchange material (Partisphere SCX, Whatman), followed by 2 cm of 5-µm C_{18} reverse phase [11]. The loaded microcapillary column was placed in-line with a Quaternary Agilent 1100 series HPLC pump. Overflow tubing was used to decrease the flow rate from 0.1 ml/min to about 200–300 nl/min. Fully automated 10 step chromatography runs were carried out [12]. Three different elution buffers were used: 5% acetonitrile, 0.1% formic acid (Buffer A); 80% acetonitrile, 0.1% formic acid (Buffer B); and 0.5 M ammonium acetate, 5% acetonitrile, 0.1% formic acid (Buffer C). Peptides were sequentially eluted from the SCX resin to the reverse phase resin by increasing salt steps, followed by an organic gradient. The last two chromatography steps consisted in a high salt wash with 100% Buffer C followed by the acetonitrile gradient. The application of a 2.5 kV distal voltage electrosprayed the eluting peptides directly into a LTQ linear ion trap mass spectrometer equipped with a nano-LC electrospray ionization source (ThermoFinnigan). Full MS spectra were recorded on the peptides over a 400 to 1,600 *m*/*z* range, followed by five

tandem mass (MS/MS) events sequentially generated in a data-dependent manner on the first to fifth most intense ions selected from the full MS spectrum (at 35% collision energy). Mass spectrometer scan functions and HPLC solvent gradients were controlled by the Xcalibur data system (ThermoFinnigan). SEQUEST [13] was used to match MS/MS spectra to peptides in a database of 61437 amino acid sequences, consisting of 35742 *Human* proteins (non-redundant entries from NCBI 2008-03-04 release), 177 usual contaminants such as human keratins, IgGs, and proteolytic enzymes and to estimate false discovery rates (FDR), 30723 randomized sequences (keeping the same amino acid composition and length) for each non-redundant protein entry. The validity of peptide/spectrum matches was assessed using the SEQUEST-defined parameters, cross-correlation score (XCorr) and normalized difference in cross-correlation scores (DeltCn). Spectra/peptide matches were only retained if they had a DeltCn of at least 0.08 and, minimum XCorr of 1.8 for singly-, 2.5 for doubly-, and 3.5 for triply charged spectra. In addition, the peptides had to be fully-tryptic and at least 7 amino acids long. Combining all runs, proteins had to be detected by at least 2 such peptides, or 1 peptide with 2 independent spectra. Proteins that were subset of others were removed. DTASelect/CONTRAST [14] was used to select, sort and compare peptide/spectrum matches passing this criteria set. Under these criteria, the %FDR ranges from 0 to 1.81. To estimate relative protein levels, spectral counts were normalized [15,16]: for each non-redundant protein *k* detected in a particular MudPIT analysis, Normalized Spectral Abundance Factors (NSAFs) were calculated as follow :

$$(NSAF)_k = \frac{(SpC/Length)_k}{\sum_{i=1}^{N}(SpC/Length)_i}$$

To further refine spectral counting, a new algorithm was implemented on this dataset to deal with peptides shared between multiple proteins. Spectral counts for peptides

shared between proteins are counted only once, and distributed according to the spectral count contribution of peptides unique to each isoform (as a way to estimate the relative proportion between isoforms). NSAF are then calculated based on distributed spectral counts (*dSpC*) with shared spectral counts distributed amongst protein isoforms [17].

Yeast two hybrid analysis. Full length or fragments of LT and FAM111A were tested against each other in a yeast two-hybrid matrix-style experiment in both directions as either GAL4 DNA-binding domain or GAL4 transactivation domain fusion proteins and performed as previously described [18-21].

4. Supplementary References

1. Welcker M, Clurman BE (2005) The SV40 large T antigen contains a decoy phosphodegron that mediates its interactions with Fbw7/hCdc4. J Biol Chem 280: 7654-7658.
2. Poulin DL, DeCaprio JA (2006) The carboxyl-terminal domain of large T antigen rescues SV40 host range activity in trans independent of acetylation. Virology 349: 212-221.
3. Poulin DL, Kung AL, DeCaprio JA (2004) p53 targets simian virus 40 large T antigen for acetylation by CBP. J Virol 78: 8245-8253.
4. Pipas JM, Peden KW, Nathans D (1983) Mutational analysis of simian virus 40 T antigen: isolation and characterization of mutants with deletions in the T-antigen gene. Mol Cell Biol 3: 203-213.
5. Pipas JM (1985) Mutations near the carboxyl terminus of the simian virus 40 large tumor antigen alter viral host range. J Virol 54: 569-575.
6. Litovchick L, Sadasivam S, Florens L, Zhu X, Swanson SK, et al. (2007) Evolutionarily conserved multisubunit RBL2/p130 and E2F4 protein complex represses human cell cycle-dependent genes in quiescence. Mol Cell 26: 539-551.
7. Skaar JR, Florens L, Tsutsumi T, Arai T, Tron A, et al. (2007) PARC and CUL7 form atypical cullin RING ligase complexes. Cancer Res 67: 2006-2014.
8. Adelmant G, Cardoza J, Ficarro S, Sikorski T, Zhang Y, et al. (2011) Affinity and Chemical Enrichment for Mass Spectrometry-Based Proteomics Analyses. In: Ivanov AR, Lazarev AV, editors. Sample Preparation in Biological Mass Spectrometry: Springer Netherlands. pp. 437-486.
9. Ficarro SB, Zhang Y, Carrasco-Alfonso MJ, Garg B, Adelmant G, et al. (2011) Online nanoflow multidimensional fractionation for high efficiency phosphopeptide analysis. Mol Cell Proteomics 10: O111 011064.
10. Washburn MP, Wolters D, Yates JR, 3rd (2001) Large-scale analysis of the yeast proteome by multidimensional protein identification technology. Nat Biotechnol 19: 242-247.
11. McDonald WH, Ohi R, Miyamoto DT, Mitchison TJ, Yates Iii JR (2002) Comparison of three directly coupled HPLC MS/MS strategies for identification of proteins from complex mixtures: single-dimension LC-MS/MS, 2-phase MudPIT, and 3-phase MudPIT. International Journal of Mass Spectrometry 219: 245-251.
12. Florens L, Washburn MP (2006) Proteomic analysis by multidimensional protein identification technology. Methods Mol Biol 328: 159-175.
13. Eng JK, McCormack AL, Yates III JR (1994) An approach to correlate tandem mass spectral data of peptides with amino acid sequences in a protein database. Journal of the American Society for Mass Spectrometry 5: 976-986.
14. Tabb DL, McDonald WH, Yates JR, 3rd (2002) DTASelect and Contrast: tools for assembling and comparing protein identifications from shotgun proteomics. J Proteome Res 1: 21-26.
15. Zybailov B, Mosley AL, Sardiu ME, Coleman MK, Florens L, et al. (2006) Statistical analysis of membrane proteome expression changes in Saccharomyces cerevisiae. J Proteome Res 5: 2339-2347.
16. Paoletti AC, Parmely TJ, Tomomori-Sato C, Sato S, Zhu D, et al. (2006) Quantitative proteomic analysis of distinct mammalian Mediator complexes using normalized spectral abundance factors. Proc Natl Acad Sci U S A 103: 18928-18933.
17. Zhang Y, Wen Z, Washburn MP, Florens L (2010) Refinements to label free proteome quantitation: how to deal with peptides shared by multiple proteins. Anal Chem 82: 2272-2281.

18. Rual JF, Hill DE, Vidal M (2004) ORFeome projects: gateway between genomics and omics. Curr Opin Chem Biol 8: 20-25.
19. Rual JF, Venkatesan K, Hao T, Hirozane-Kishikawa T, Dricot A, et al. (2005) Towards a proteome-scale map of the human protein-protein interaction network. Nature 437: 1173-1178.
20. Yu H, Braun P, Yildirim MA, Lemmens I, Venkatesan K, et al. (2008) High-quality binary protein interaction map of the yeast interactome network. Science 322: 104-110.
21. Boxem M, Maliga Z, Klitgord N, Li N, Lemmens I, et al. (2008) A protein domain-based interactome network for C. elegans early embryogenesis. Cell 134: 534-545.
